# Foundation Models Meet Medical Image Interpretation

**DOI:** 10.34133/research.1024

**Published:** 2026-02-27

**Authors:** Licheng Jiao, Jiayao Hao, Ruiyang Li, Lingling Li, Xu Liu, Fang Liu, Wenping Ma, Puhua Chen, Zhongjian Huang, Jingyi Yang, Jiaxuan Zhao, Qigong Sun

**Affiliations:** ^1^ School of Artificial Intelligence, Xidian University, Xi’an, China.; ^2^ Sense Time, Shanghai, China.

## Abstract

Facing challenges such as limited annotated data and insufficient model generalization in medical deep learning, foundation models (FMs) are reshaping the paradigm of medical image interpretation through large-scale pretraining and efficient fine-tuning. Unlike traditional models focused on single modality and task, FMs enable multi-modal representation and task-agnostic transfer, adapting to various downstream applications without extensive annotation or retraining. This paper systematically reviews the research progress on medical FMs, focusing on medical tasks, datasets, and evaluation metrics. It covers key interpretation tasks such as classification, segmentation, generation, and prognosis prediction. At the data level, it integrates multi-source data including 2-dimensional (2D)/3D medical imaging, vision-language data, electronic health records (EHRs), physiological signals, and bioinformatics data, and summarizes the evaluation metrics for each task. On this basis, the paper categorizes and analyzes mainstream medical FMs, including pretrained models, vision FMs, vision-language FMs, and extended multi-modal FMs, providing a systematic comparison of their performance and characteristics. Furthermore, we innovatively proposes the IPIU medical FM platform, which integrates large-scale medical data, universal vision models, medical vision-language models, and medical large language models, and verifies its effectiveness in typical clinical tasks. In addition, this work is the first to systematically analyze the key challenges and emerging trends of medical FMs across 12 critical dimensions, including data, modeling, security, and computational resources, filling the gaps in the existing reviews in systematic sorting and forward-looking analysis. Our aim is to provide theoretical support and practical reference for the sustainable development of medical FMs. Related resources and literature lists will be open sourced on https://github.com/JYAOii/Foundation-Models-meet-Medical-Image-Interpretation.

## Introduction

### Background

**Fig. 1.**
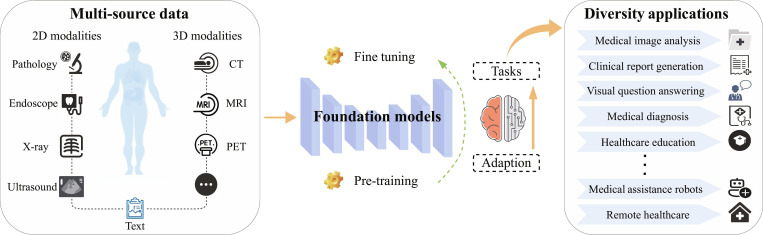
Medical foundation model is pretrained on large-scale multi-modal medical data to learn generalizable representations and subsequently fine-tuned for diverse clinical downstream tasks.

Foundation models (FMs), as a significant paradigm innovation in artificial intelligence (AI) [1], were systematically introduced by Stanford University in 2021 [2]. Its core concept is to learn universal representation through pretraining on large-scale multi-modal data. Combined with parameter-efficient fine-tuning (PEFT) strategies, FMs enable rapid adaptation to various downstream tasks, as illustrated in Fig. 1. Representative models such as GPT-3 [3] and MedCLIP [4], based on the Transformer architecture, use self-attention mechanisms for context-aware modeling. These models have demonstrated superior generalization capabilities in natural language processing, computer vision, and biomedical domains [5,6].

In contrast, traditional deep learning methods in the medical domain have long been constrained by scarce annotations data, weak cross-modal semantic correlation, and insufficient generalization capabilities. FMs can effectively alleviate these issues by extracting semantic representations from large-scale unlabeled data, reducing dependence on expert annotations, and enhancing cross-modal understanding and transferability [7]. This provides technical support to address challenges such as long-tail distributions, data scarcity, and modality imbalance, thereby promoting a shift in medical decision-making from experience-driven to data-driven approaches.

Unlike traditional specialist models such as nnU-Net [8], which are typically designed for a single modality and specific tasks, FMs emphasize modality unification and task generalization, enabling cross-domain transfer and knowledge sharing. With mechanisms such as prompt engineering and PEFT, these models support few-shot and even zero-shot transfer (ZST). For example, Med-PaLM [9] is based on a unified medical pretraining model, which can generate structured pathology reports and perform lesion localization from medical images. It effectively overcomes the limitations of traditional methods that require separate architectures for different tasks, significantly improving modeling efficiency and system integration. Driven by such unified model architecture, medical AI systems are evolving toward greater generality and reusability.

Despite these advancements, the unique characteristics of the medical domain pose multiple challenges to the application of FMs. On one hand, medical data are highly heterogeneous, with pronounced differences in resolution, contrast, and noise distribution across imaging modalities such as computed tomography (CT), magnetic resonance imaging (MRI), and ultrasound [10]. This limits the ability of traditional single-modality pretraining strategies to achieve effective cross-domain knowledge integration. On the other hand, clinical applications demand higher standards for model performance. Clinical decision-making relies on interpretable diagnostic evidence, yet pretraining models often behave as “black boxes”, limiting their clinical traceability [11]. In addition, the long-tail distribution of rare diseases poses fairness challenges for model generalization [12]. Privacy concerns and computational constraints further complicate model training. The sensitivity of medical data hinders the collection of large-scale pretraining datasets. Although federated learning can mitigate the risk of privacy leakage to some extent, issues such as data heterogeneity and limited communication efficiency still impede model convergence [13]. Addressing these challenges requires ongoing progress in multi-modal representation learning, interpretable model architecture design, and comprehensive evaluation frameworks.

### Comparison of related reviews

Several existing reviews have provided valuable insights into FMs in medical imaging [7,12,14–18], each differing in focus and scope. For example, Khan et al. [7] systematically summarized the latest research up to early 2024, covering the development history, classification systems, application scenarios, major challenges, and future directions of medical FMs. VanBerlo et al. [14] focused on the impact of self-supervised pretraining on radiological imaging tasks (MRI, CT, x-ray, and ultrasound) and highlighted future research priorities. He et al. [15] reviewed over 200 medical FMs studies up to early 2024, proposed a novel classification system, organized datasets and typical applications, and discussed key challenges and prospects. Moor et al. [16] introduced a new paradigm of general medical AI (GMAI), detailing its potential and applications in multi-modal medical image interpretation and outlining implementation challenges. Zhang and Metaxas [17] described the spectrum of medical FMs, from general imaging models to modality-specific and organ/task-specific models, emphasizing their applications, challenges, and future development in medical image analysis. Liu et al. [18] reviewed VLFMs in medicine, summarizing pretraining and fine-tuning strategies, zero-shot capabilities, and multi-modal interpretability, while analyzing key challenges such as privacy and explainability. Ryu et al. [19] systematically reviewed recent advances in medical VLFMs from 2022 to 2024, proposed a structured taxonomy covering models, data, and applications, and summarized key challenges and future directions. FairMedFM [12] systematically evaluated 20+ medical imaging FMs across multi-modal and multi-task settings, revealing biases and limitations of mitigation strategies. Table 1 provides a systematic comparison of these reviews in terms of research content, focus, and coverage.

**Table 1.** Comparison of existing reviews on representative medical foundation models

Related reviews	Data	Evaluation metrics	Pretrain	VFMs	VLFMs	Applications	Challenges	Future directions
Khan et al. [7]	**✗**	**✗**	**✗**	✓	✓	✓	✓	✓
VanBerlo et al. [14]	✓	**✗**	✓	**✗**	**✗**	✓	**✗**	✓
He et al. [15]	**✗**	**✗**	✓	✓	✓	**✗**	✓	✓
Moor et al. [16]	**✗**	**✗**	**✗**	**✗**	**✗**	✓	✓	✓
Zhang and Metaxas [17]	✓	**✗**	**✗**	**✗**	✓	✓	**✗**	✓
Liu et al. [18]	**✗**	**✗**	✓	**✗**	✓	✓	✓	✓
Ryu et al. [19]	✓	**✗**	**✗**	**✗**	✓	✓	✓	✓
FairMedFM [12]	✓	✓	**✗**	✓	✓	**✗**	✓	**✗**
This review	✓	✓	✓	✓	✓	✓	✓	✓

**Fig. 2.**
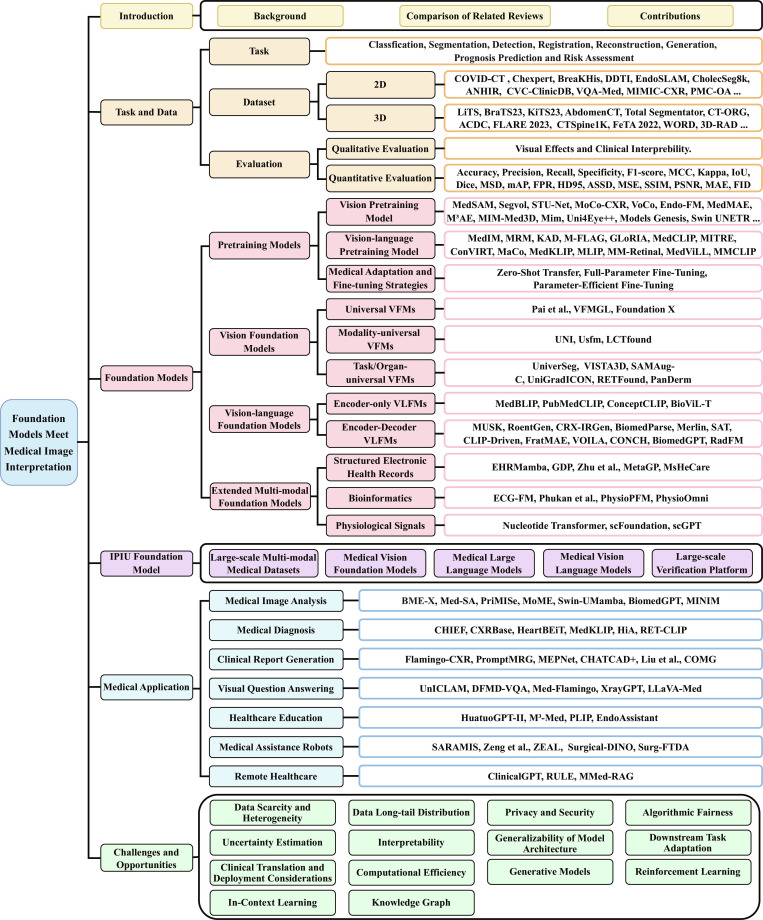
Overview of the key components of this paper, including medical data and evaluation metrics, foundation models, the proposed IPIU medical foundation model platform, medical practical applications, and challenges and opportunities.

Building on the above reviews, this paper provides the first comprehensive and structured survey of medical FMs across data, evaluation metrics, model mechanisms, applications, challenges, and future directions, aiming to offer a more complete understanding of their role in medical image interpretation. The overall framework is illustrated in Fig. 2.

### Contributions

In this work, we focus on medical image interpretation, which encompasses not only the perception and understanding of medical images but also the semantic extraction and analysis through computational algorithms [20]. Within this framework, common medical image analysis tasks can be regarded as different facets of interpretation: Classification corresponds to recognizing the overall semantic content of images [8]; segmentation and detection enable spatial localization and delineation of lesions or organs [8]; registration and reconstruction facilitate cross-modality and longitudinal alignment as well as image quality restoration [21]; and generative models extend the depth and breadth of interpretation by synthesizing or predicting images [10]. Furthermore, prognosis prediction and risk assessment target clinical decision-making by inferring disease progression trends and individualized risk levels from imaging features [22]. Collectively, classification, segmentation, detection, registration, reconstruction, generation, prognosis prediction, and risk assessment have distinct focuses, and they share the common goal of deriving structured and interpretable information from complex medical images to support clinical diagnosis and research applications. Based on this, the main contributions of this work are as follows:

1. Comprehensive overview: This article systematically reviews the development of FMs in medical image interpretation from a global perspective for the first time. It systematically reviews key components including datasets and evaluation metrics, model architectures, the proposed Key Laboratory of Intelligent Perception and Image Understanding (IPIU) medical FM platform, representative application scenarios, as well as major challenges and future directions. The review provides a holistic view of how FMs empower end-to-end medical image analysis.
2. Datasets and evaluation metrics: Considering the unique characteristics of medical imaging tasks, we systematically review major publicly available medical imaging datasets and their associated downstream vision tasks. It also provides the first unified summary of commonly used evaluation metrics across tasks such as image classification, segmentation, detection, and generation, offering a reference for standardized model evaluation.
3. Systematic classification of FMs: This paper categorizes and analyzes current mainstream medical FMs from 3 key dimensions: pretraining paradigms, vision foundation models (VFMs), and vision-language foundation models (VLFMs).
4. IPIU medical FM platform: This paper introduces the IPIU platform, which integrates large-scale multi-modal medical datasets and incorporates advanced VFMs, large medical language models, and VLFMs to enable fair and comprehensive analysis across multiple tasks and modalities in medical imaging.
5. Challenges and future directions: This paper systematically summarizes the core challenges faced by current medical FMs across 12 critical dimensions, including data scarcity, model generalizability, interpretability, privacy and security, and generative modeling, and provides an in-depth discussion of future research trends and development directions.

In summary, this paper offers a systematic analysis from the perspectives of technical principles, application scenarios, and future challenges, addressing gaps in existing reviews regarding comprehensive synthesis and forward-looking trend analysis, while aiming to provide theoretical support and practical guidance for ongoing innovation and clinical translation of medical FMs.

The organization of this paper is as follows: Task and Data analyzes the characteristics of medical imaging tasks and systematically reviews typical visual downstream tasks and commonly used evaluation metrics. Medical FMs classifies and compares existing medical FMs based on pretraining strategies, model architectures, and applicability. IPIU Medical FM Platform introduces the IPIU medical FM platform for multi-task and multi-modal analysis, and conducts a systematic evaluation of mainstream methods under a standardized workflow. Medical Applications of FMs summarizes the practical applications of FMs in typical medical imaging interpretation tasks. Challenges and Opportunities deeply discusses key challenges and future directions. Finally, Conclusion concludes the paper and provides an outlook on the future development of medical FMs.

## Task and Data

### Typical tasks

Medical image interpretation is a critical component in clinical diagnosis and treatment, playing an important role in disease screening, diagnosis, and treatment evaluation [23]. With the advancement of computational intelligence methods and medical imaging technology, the interpretation of massive high-dimensional images has put forward higher requirements for the accuracy and efficiency of medical image interpretation [24]. In this context, core tasks such as organ segmentation, lesion detection, and image generation have been widely used in auxiliary diagnosis and pre- and post-operative evaluation. FMs, with their large-scale pretraining and strong contextual understanding, mitigate the need for extensive annotated data and enhance generalization across heterogeneous and multi-modal medical imaging tasks [16]. The following sections provide a systematic overview of representative tasks enabled by FMs in medical imaging.

#### Classification

Medical image classification aims to learn the mapping relationship between image features and disease categories. It is widely applied in clinical scenarios such as pneumonia detection and tumor staging, providing essential support for early diagnosis [25]. However, this task faces challenges, including modality differences, imbalanced data distribution, and the scarcity of long-tail samples. Traditional models often struggle with generalization and transferability under such conditions [26]. In recent years, medical FMs have leveraged large-scale pretraining and parameter-sharing mechanisms to effectively mitigate data scarcity and modality gaps. These models enhance the recognition of complex pathological features and lay a theoretical foundation for building a general medical image classification system with unified perception and reasoning capabilities [15].

#### Segmentation

Medical image segmentation aims to perform pixel-level or voxel-level partitioning to accurately localize specific anatomical structures or lesion regions. It serves as a fundamental step in supporting clinical diagnosis and treatment decision-making [27]. This task faces several challenges, including inconsistent resolutions across imaging modalities, significant anatomical variability, and blurred lesion boundaries. Traditional methods often show limited generalization and adaptability across tasks. FMs, with their unified architectures and large-scale pretraining, have significantly improved segmentation performance. They also demonstrate strong transferability in small-sample scenarios and multi-center datasets [17]. In addition, the introduction of vision-language models (VLMs) further advanced text-guided segmentation strategies, enhancing the model’s ability to understand and represent complex medical contexts [28].

#### Detection

Medical detection tasks aim to identify and localize lesion areas in medical images to support early diagnosis and precision treatment [29]. The core challenge lies in extracting diagnostically relevant visual features from high-dimensional and complex imaging data. This task is widely applied in various clinical scenarios, including fracture detection, tumor screening, polyp localization, and other disease scenarios. The FMs have improved the detection accuracy and adaptability of complex lesions through cross-modal pretraining and unified representation learning. They are gradually enabling a shift from task-specific models to more generalized detection paradigms [30]. However, existing medical detection model still face limitations in dealing with individual differences and data distribution shifts. Therefore, enhancing their ability to model abnormal sample distributions is critical to improving robustness and reliability in anomaly recognition.

#### Registration

Medical image registration aims to spatially align medical images acquired at different time points, from different modalities, or using different devices, enabling the unified fusion and comparative analysis of multi-source information [21]. It serves as a key technology for multi-modal fusion, image-guided therapy, and disease monitoring. The core challenge is to establish an optimal spatial mapping to maximize anatomical consistency. In recent years, FMs have demonstrated strong structural modeling and cross-modal alignment capabilities in registration tasks. They have shown potential in improving registration accuracy and robustness, particularly in challenging scenarios such as complex organ matching and modality discrepancy modeling [31]. However, their generalization and clinical adaptability still require further enhancement when dealing with practical challenges such as imaging artifacts, individual differences, and pathological deformations [32].

#### Reconstruction

Medical image reconstruction aims to restore high-quality 3-dimensional (3D) or 4D medical images from raw medical data through computational imaging and signal processing techniques to make up for the limitations of physical imaging systems and improve the anatomical clarity and diagnostic value [33]. It is widely applied in modalities such as CT, MRI, and positron emission tomography–computed tomography (PET-CT), and is particularly critical in scenarios like low-dose CT and MRI. In recent years, FMs have shown strong potential in this field by leveraging cross-modal modeling and data-driven inversion methods. For example, diffusion models have been used for high-fidelity reconstruction of undersampled MRI data, while generative adversarial networks combined with physical priors have improved the quality of low-dose CT images. However, there are still challenges such as data sparsity, imaging artifacts, and insufficient interpretability of reconstruction. There is an urgent need to strike a balance between preserving structural fidelity and recovering fine-grained details.

#### Generation

Medical image generation leverages deep generative models to learn the distribution of real medical images and synthesize high-quality medical images. This helps alleviate challenges such as data scarcity, high annotation costs, and class imbalance [34]. The task has been widely applied in training data augmentation, few-shot lesion detection, image denoising, and privacy protection. In recent years, FMs have demonstrated excellent image modeling and cross-modal translation capabilities in generation tasks. For example, high-quality synthetic CT images have been generated from MRI using diffusion models with shifted window Transformers [35], and generative adversarial networks have been used to synthesize lesion-specific image features [36]. However, the lack of a unified, low-cost, and generalizable evaluation framework remains a major challenge, which limits the clinical reliability and application value of generated images.

#### Prognosis prediction and risk assessment

Medical prognosis prediction aims to infer patients’ disease-free survival, overall survival, or recurrence risk, based on medical images, pathology slides, and related clinical information, thereby providing a basis for downstream risk assessment to enable risk stratification and personalized treatment decisions [22]. For instance, Wang et al. [37] developed a pathology FM trained on over 100,000 whole-slide images, demonstrating robust performance in prognosis prediction and risk assessment for gastric and colorectal cancers (CRC), enabling risk stratification independent of clinicopathological factors and assisting in evaluating the potential benefits of adjuvant chemotherapy. Pai et al. [38] trained an FM using self-supervised learning on 11,467 CT scans for quantitative cancer biomarkers discovery, achieving superior performance over conventional supervised models in downstream tasks, including anatomical lesion classification, pulmonary nodule malignancy risk assessment, and non-small cell lung cancer prognosis prediction. Peng et al. [39] proposed a continually evolving multi-modal FM that effectively integrates multiple data modalities through pseudo-target generation and instruction-based knowledge distillation (KD), significantly enhancing cancer prognosis prediction. Nevertheless, prognosis prediction and risk assessment still faces challenges such as long follow-up periods, high annotation costs, and imbalanced labels, highlighting the need for large-scale, multi-modal, longitudinal datasets to improve model generalization and clinical interpretability.

### Medical imaging data

Medical imaging datasets exhibit significant heterogeneity in dimensions, task, anatomical region, and modality [10]. Compared to natural images, medical data are often constrained by privacy concerns, high annotation costs, and cross-institutional variability, which limit model accessibility and generalization [7]. Therefore, developing high-quality datasets that encompass both anatomical structure and pathological features has become essential for advancing medical FMs.

To systematically review the datasets used in current mainstream medical FMs, this study adopts a hierarchical organization of “dimensionality → task → modality” to ensure clear and interpretable classification. Specifically, datasets are first categorized by dimensionality into 2D and 3D to reflect differences in structural information captured by each imaging type. Within each dimensionality, datasets are further grouped by task, including classification, segmentation, detection, reconstruction, medical visual question answering (Med-VQA), and medical report generation (MRG), highlighting the modeling objectives of each dataset. Finally, within each task, datasets are subdivided by imaging modality (MRI, CT, x-ray, ultrasound, etc.) to clearly present the characteristics and clinical applications of different modalities. This hierarchical organization effectively avoids overlaps between task and modality, making the classification of datasets in tables and figures more intuitive and transparent.

Tables 2 and 3 provide a systematic summary of the key medical image datasets used in current mainstream medical FMs, following the classification scheme described above.

**Table 2.** Summary of 2D image datasets widely used in medical foundation models. The original publications or official sources of the datasets are provided in Table S1.

Task	Anatomical region	Imaging method	Dataset
Classification	Whole body	Pathology	NAFLD
	Head and neck	Pathology	OSCC
	Thorax	CT	COVID-CT, SARS-COV-2 Ct-Scan, Chest CT-Scan images
		X-ray	COVID-19 CHEST X-RAY, COVIDGR, MIAS, CoronaHack
		Ultrasound	BUSI
		Pathology	BreaKHis, MIDOG++, LungHist700
	Abdomen	Pathology	Gleason 2019, NuCLS, PANDA, RenalCell
		Endoscope	Colonoscopic, LIMUC, The Nerthus Dataset, CP-CHILD
	Eyes	OCT	Retinal OCT-C8, OCT 2017
		Fundus images	RFMiD 2.0, MuReD, REFUGE, ToxoFundus
Segmentation	Head and neck	Ultrasound	TN-SCUI 2020, DDTI, TG3K, TN3K
	Thorax	Ultrasound	Breast Ultrasound Dataset B, BUSI
	Abdomen	Endoscope	Kvasir-SEG, CVC-ClinicDB, CholecSeg8k, EAD 2019
		Pathology	EBHI-Seg, SegPANDA200, CRAG
	Multi-organs	Multi-modality	IMIS-Benchmark
Detection	Abdomen	Endoscope	LDPolypVideo, LIMUC
Registration	Thorax	Pathology	ANHIR
Reconstruction	Abdomen	Endoscope	EndoSLAM, EndoMappe, C3VD
Med-VQA	Multi-regions	Multi-modality	PMC-OA, PMC-VQA, VQA-RAD, VQA-Med, SLAKE,
			PathVQA, MIMIC-Diff-VQA, Gemex
Report generation	Thorax	Multi-modality	MIMIC-CXR, CheXpert, MIMIC-NLE, CXR-PRO,
			MS-CXR, IU X-ray
	Multi-regions	Multi-modality	ROCOv2

**Table 3.** Summary of 3D image datasets widely used in medical foundation models. The original publications or official sources of the datasets are provided in Table S2.

Task	Anatomical region	Imaging method	Dataset
Classification	Multi-regions	Multi-modality	RP3D-DiagDS
Detection	Thorax	CT	LUNA16
	Skeleton	CT	RibFrac 2020
		MR	Lumbosacral spine MRI
Segmentation	Whole body	CT	TotalSegmentator v1.0, CT-ORG, AutoPET
		MR	TotalSegmentator MRI
	Head and neck	CT	SegRap2023, HaN-Seg, PDDCA, HECKTOR 2022
	Brain	CT	INSTANCE 2022
		MR	FeTA 2022, iSeg, BraTS21, ISLES22, ATLAS v2.0, WMH
	Thorax	CT	ATM22, Parse 2022, SegTHOR, PleThora, FUMPE
		MR	ACDC, LAScarQS 2022, MyoPS 2020, CMRxMotion
		Ultrasound	MVSeg-3DTEE 2023, TDSC-ABUS2023
	Abdomen	CT	FLARE 2022, WORD, AbdomenCT-1K, 3D-IRCADB, LiTS
			KiTS23, BTCV, MSD, PanSegData, MOOD2022
		MR	AMOS 2022, ATLAS, SPPIN, CHAOS, PROMISE12
	Skeleton	CT	VerSe, CTSpine1K, CTPelvic1K
		MR	SPIDER
	Vessel	CTA	SEG.A., KiPA22, ImageCAS
Registration	Brain	MR	L2R-OASIS
Med-VQA	Multi-regions	Multi-modality	3D-RAD
Report generation	Multi-regions	Multi-modality	RP3D-Caption

At the task level, medical imaging datasets reflect distinct modeling priorities: Classification datasets support disease or lesion type identification [25]; segmentation datasets focus on precise delineation of organs or lesion regions; detection datasets emphasize spatial localization of target; registration datasets align multi-phase or multi-modal images to integrate structural and functional information; reconstruction datasets aim to restore 3D structures or image quality [10]. Meanwhile, Med-VQA and report generation datasets combine imaging with natural language to enable cross-modal semantic understanding and automated diagnostic modeling. Given the substantial differences in research objectives and clinical applications across tasks, organizing datasets by task type clarifies modeling goals and guides methodological choices. Detailed descriptions of each task type and their characteristics are provided in Typical tasks.

At the anatomical level, datasets cover targets ranging from organs to specific anatomical structures. Different anatomical regions exhibit substantial variations in morphology, tissue characteristics, and pathological presentations [40]. For example, the imaging properties and focal points differ markedly among brain, bones, and vessels. Classifying datasets by anatomical region helps clarify the training and evaluation targets, enhances comparability among datasets for the same task, and facilitates analysis of model performance across different organs or anatomical regions.

At the imaging method level, different imaging techniques, leveraging their unique imaging mechanisms and clinical advantages, enrich the representational diversity of the datasets.

2D data such as x-rays, ultrasound, and endoscopic images are based on projection or surface imaging principles, enabling rapid acquisition and preliminary clinical screening. Among them, x-ray imaging relies on the transmission of radiation and is commonly used for fracture detection and chest disease screening [41]. Ultrasound employs high-frequency sound wave reflections, offering real-time imaging without radiation, and is widely applied in obstetrics, cardiology, and abdominal examinations [42]. Endoscopic images are obtained using optical probes, allowing direct visualization of lesions within the gastrointestinal or respiratory tracts [10]. Dermoscopic imaging uses polarized light to enhance the visualization of subsurface skin structures, supporting the screening of skin lesions [43].

In contrast, 3D imaging modalities such as CT, MRI, and PET-CT provide rich spatial structural information, supporting detailed modeling and dynamic analysis of complex anatomical regions. CT reconstructs 3D internal structures using multi-angle x-ray projections and is suitable for comprehensive whole-body assessments [10]. MRI, based on magnetic resonance signals, offers superior soft tissue contrast and is widely used for imaging the brain, spine, and joints [44]. PET-CT enables precise imaging of tissue metabolism and functional states through radiotracer techniques, which is widely used in the diagnosis of tumors and cardiovascular and neurological diseases [44].

Meanwhile, with the rise of multi-modal learning, vision-language medical datasets have emerged in recent years, such as Med-VQA and MRG. These datasets combine images with natural language annotations or diagnostic texts, supporting cross-modal representation learning and clinical semantic understanding in FMs [45].

However, the scope of medical multi-modal data extends far beyond the combination of images and text. To further enhance the coverage of multi-modal FMs, Table 4 systematically summarizes widely used datasets related to structured EHRs, physiological signals, and bioinformatics. These datasets encompass clinical records, physiological monitoring signals such as electrocardiogram (ECG) and electroencephalogram (EEG), as well as genomic sequencing information, providing a solid foundation for knowledge integration and representation learning across diverse medical modalities.

**Table 4.** Summary of extended multi-modality datasets widely used in medical foundation models. The original publications or official sources of the datasets are provided in Table S3.

Task	Dataset	Imaging method	Data information
Structured EHRs	MIMIC-II	2001–2008 EHRs	25,328 ICU admissions with laboratory data, interventions, vital signs, clinical notes, and waveform recordings.
	MIMIC-III	2001–2012 EHRs	53,423 adult patients admitted to the BIDMC ICU from June 2001 to October 2012, 7,870 critically ill neonates admitted from 2001 to 2008.
	MIMIC-IV	2008–2019 EHRs	Clinical data on more than 190,000 patients and 450,000 hospitalizations admitted to BIDMC, including Hospital module (demographics, measurement, and related information), ICU module, and Notes module.
	eICU Dataset	2014–2015 EHRs	Multi-center intensive care dataset comprising 200,859 admissions of approximately 140,000 unique patients.
Physiological signals	PhysioNet 2021	ECG	131,149 12-lead ECG records from 9 databases.
	MIMIC-IV-ECG	ECG	Approximately 800,000 diagnostic 12-lead ECGs from nearly 160,000 unique patients, each 10 s long and sampled at 500 Hz.
	WESAD	Physiological signals, motion signals	Comprising physiological and motion signals from 15 subjects using chest (700 Hz) and wrist (4–64 Hz) sensors for stress and affect detection.
	Sleep-EDF	EEG, EOG, chin EMG	197 whole-night PolySomnoGraphic sleep recordings, containing EEG, EOG, chin EMG, and event markers.
	DREAMER	EEG, ECG	Comprising EEG and ECG signals recorded from 23 participants during emotion elicitation via audiovisual stimuli.
	MIT-BIH arrhythmia dataset	ECG	Including over 4,000 dynamic 24-h ECG recordings from 47 subjects, totaling 109,500 heartbeats, with 30% abnormal beats.
	SEED-VII	EEG, EOG, ECG	A multi-modal emotion dataset comprising EEG, EOG, and ECG signals from 20 subjects recorded at 1,000 Hz.
	TUEG	EEG, EOG, ECG, EMG	Contains 26,846 clinical EEG recordings with occasional
	EOG, ECG, and EMG signals, sampled at 250–1,024 Hz.	
	DEAP	EEG, EOG, EMG	EEG, EOG, and EMG signals from 32 participants watching 40 one-minute
	music video clips, sampled at 512 Hz.	
	GX	EEG, ECG, EOG, tES	A multi-modal human-subject dataset combining EEG, physiological signals (ECG, EOG), and tES data from 783 trials across 62 sessions.
	SPH	ECG	A large-scale multi-label 12-lead ECG dataset from 24,666 patients with 25,770 records.
Bioinformatics	hECA	scRNA-seq	A unified human cell atlas (hECA) integrating 1,093,299 cells from 116 studies across 38 organs and 146 cell types.
	Baron dataset	scRNA-seq	A single-cell transcriptomic dataset of over 12,000 pancreatic cells from humans and mice.
	The 1000G dataset	Whole-genome sequencing	A whole-genome sequencing (WGS) dataset of 3,202 human samples from 26 populations, including 602 trios.
	DeepSEA	DNA sequence	Comprising 2.4 million sequences of 1,000 nucleotides each, annotated with 919 chromatin features.

Overall, these vision-language datasets, together with medical images, form a multi-modal medical imaging ecosystem, providing a solid data foundation for precision diagnosis and the development of FMs [10,44].

**Fig. 3.**
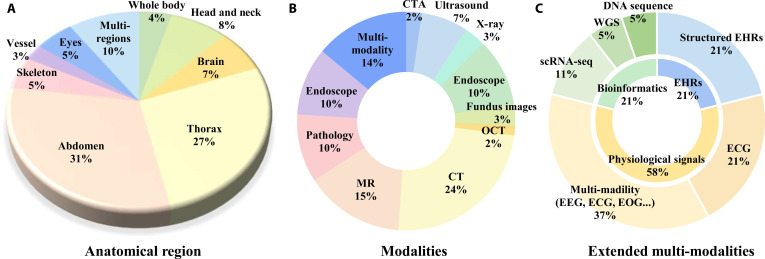
(A and B) Anatomical structure distribution and modality distribution of the datasets summarized in Tables 2 and 3. (C) Modality distribution of the extended multi-modal datasets summarized in Table 4.

Figure 3 illustrates the anatomical structures and modality distributions of the datasets summarized in Tables 2 to 4.

### Evaluation metrics

The performance evaluation of medical FMs is a critical measure of their practical value [12]. Due to the distinct characteristics of tasks such as classification, segmentation, detection, registration, reconstruction, and generation, differentiated evaluation metrics are required. A scientific and systematic evaluation system not only comprehensively reflects model performance and guides the optimization of model architecture and training strategies but also provides quantitative evidence for assessing reliability in clinical applications [46]. Overall, evaluation methods primarily consist of quantitative and qualitative metrics, which assess model effectiveness from numerical accuracy and visual quality perspectives, respectively.

#### Qualitative assessment

Qualitative metrics focus on subjective evaluation of model outputs from the perspective of visual quality and clinical interpretability, and are often achieved through image visualization analysis. In classification tasks, evaluation focuses on the consistency between the predicted results and lesion features [25]. For segmentation and detection, assessment emphasizes boundary accuracy, lesion completeness, and localization validity [31,44]. Registration tasks focus on structural continuity and anatomical rationality [32]. Reconstruction and generation consider structural fidelity, natural texture, and lesion preservation [34]. Prognosis prediction and risk assessment tasks focuses on the accuracy of risk stratification for patient prognosis and the clinical interpretability and rationality of the model’s predictive outcomes [22].

Common qualitative evaluation methods include expert blind review and subjective scoring, which are used to supplement the dimensions that quantitative indicators cannot fully cover, especially in evaluating the clinical applicability and semantic integrity of the model [47]. In generative tasks, the most widely adopted approach is the mean opinion score (MOS), in which multiple radiologists or clinical experts rate the generated images, and the average score is taken as an indicator of the subjective quality of the model outputs. To enhance clinical interpretability, MOS can be combined with perceptual quality metrics such as the structural similarity index measure (SSIM) to cross-validate the consistency of generated images in terms of structure, texture, and luminance. In addition, saliency maps or attention-based visualizations, such as Grad-CAM, can be employed to evaluate whether the generation process focuses on key lesions or anatomical structures, thereby providing visual evidence to support clinical decision-making and further validating the reliability and clinical plausibility of the generated results.

In prognostic prediction and risk assessment tasks, Kaplan–Meier survival analysis is employed to estimate time-dependent survival probabilities, while risk group curves and log-rank tests are used to evaluate the model’s ability to distinguish between high- and low-risk patients [38]. This approach integrates qualitative visualization with statistical significance testing, effectively illustrating survival differences between risk groups and providing an intuitive validation of the model’s prognostic discrimination and clinical plausibility.

#### Quantitative evaluation

Quantitative metrics provide objective and reproducible numerical evaluations of model outputs in terms of accuracy, stability, and generalization ability, serving as the core basis for performance assessment.

Compared to qualitative analysis, quantitative metrics provide a more standardized basis for comparison. Table 5 systematically summarizes the commonly used quantitative evaluation methods and their calculation formulas in various tasks [46].

**Table 5.** Summary of evaluation metrics for medical image interpretation tasks in foundation models. ↑ indicates that a higher value implies better performance, and ↓ indicates that a lower value is preferable.

Task	Evaluation metrics	Calculation formula	Explanation
Classification	Accuracy [47] ↑	$\text{Accuracy}=\frac{\mathrm{TP}+\mathrm{TN}}{\mathrm{TP}+\mathrm{FP}+\mathrm{FN}+\mathrm{TN}}$	The number of correct predictions among all predictions.
	Precision [47] ↑	$\text{Precision}=\frac{\mathrm{TP}}{\mathrm{TP}+\mathrm{FP}}$	The proportion of predicted positives that are truly positive.
	Recall [47] ↑	$\text{Recall}=\frac{\mathrm{TP}}{\mathrm{TP}+\mathrm{FN}}$	Sensitivity; the proportion of actual positives that are correctly predicted.
	Specificity [47] ↑	$\text{Specificity}=\frac{\mathrm{TN}}{\mathrm{TN}+\mathrm{FP}}$	The proportion of actual negatives that are correctly predicted.
	*F*_1_ score [47] ↑	$F_{1}\text{Score}=2\times \frac{\text{Precision}\times \text{Recall}}{\text{Precision}+\text{Recall}}$	The harmonic mean of precision and recall.
	AUC [48] ↑	$\mathrm{AUC}=\frac{\sum_{\left(p_{i},\;n_{j}\right),\;p_{i}>n_{j}}1}{P\times N}$	Area under curve, less sensitive to sample class imbalance.
	AUROC [46] ↑	$\text{AUROC}=\int_{0}^{1}\mathrm{TPR}\left(\mathrm{FPR}\right)\;d\left(\mathrm{FPR}\right)$	Area under the ROC curve, measuring classification ability.
	MCC [47] ↑	$\mathrm{MCC}=\frac{\mathrm{TP}\times \mathrm{TN}-\mathrm{FP}\times \mathrm{FN}}{\sqrt{\left(\mathrm{TP}\;+\;\mathrm{FP}\right)\left(\mathrm{TP}\;+\;\mathrm{FN}\right)\left(\mathrm{TN}\;+\;\mathrm{FP}\right)\left(\mathrm{TN}\;+\;\mathrm{FN}\right)}}$	Consider all confusion matrix elements, suitable for imbalanced data.
	Kappa [49] ↑	$\text{Kappa}=\frac{\mathrm{Po}-\mathrm{Pe}}{1-\mathrm{Pe}}$	Measure the consistency between model predictions and ground truth.
	Net benefit [46] ↑	$\mathrm{NB}=\frac{\mathrm{TP}-w\cdot \mathrm{FP}}{N}$	Measure the actual clinical benefits of the model.
	Expected cost [46] ↓	$\mathrm{EC}=\sum_{i,j}P\left(i\right)\cdot C\left(i,\;j\right)$	Expected loss of the cost of class error.
	${\mathrm{LR}}^{+}$ [46] ↑	${\mathrm{LR}}^{+}=\frac{\text{Sensitivity}}{1-\text{Specificity}}$	The value of a positive test result in confirming disease.
	Brier score [46] ↓	$\mathrm{BS}=\frac{1}{N}\sum_{i=1}^{N}{\left(p_{i}-y_{i}\right)}^{2}$	Measure the deviation between predicted probabilities and true labels.
Segmentation	Pixel accuracy [49] ↑	$\mathrm{PA}=\frac{\sum_{i=0}^{K}p_{\mathrm{ii}}}{\sum_{i=0}^{K}\sum_{j=0}^{K}p_{\mathrm{ij}}}$	Pixel-level classification accuracy.
	IoU [48] ↑	$\mathrm{IoU}=\frac{|A\cap B|}{|A\cup B|}$	The degree of overlap between the predicted and ground truth regions.
	Dice [49] ↑	$\text{Dice}=\frac{2|A\cap B|}{|A|+|B|}$	Measure the similarity between predicted and ground truth samples.
	clDice [46] ↑	$\text{clDice}=\frac{2\cdot |P\cap T|}{|P|+|T|}$	Dice based on centerline similarity; suitable for tubular structures segmentation.
	HD [46] ↓	$\mathrm{HD}=\text{max}\;\left(h\left(X,\;Y\right),\;h\left(Y,\;X\right)\right)$	Maximum boundary distance, sensitive to outliers.
	ASSD [48] ↓	$\text{ASSD}=\frac{\sum_{x\in X}\min_{y\in Y}d\left(x,\;y\right)+\sum_{y\in Y}\min_{x\in X}d\left(y,\;x\right)}{|X|+|Y|}$	Average symmetric surface distance.
	MASD [46]	$\text{MASD}=\frac{1}{|S_{P}|+|S_{T}|}\sum_{x\in S_{P}\cup S_{T}}d\left(x,\;S_{T}\cup S_{P}\right)$	Mean absolute surface distance.
	NSD [47] ↑	$\mathrm{NSD}=\frac{\left|\;\left\{p\in S_{P}\;|\;d\left(p,\;S_{G}\right)\leq \tau \right\}\;\right|+\left|\;\left\{g\in S_{G}\;|d\left(g,\;S_{P}\right)\leq \tau \;\right\}\right|\;}{|S_{P}\;|+|S_{G}\;|}$	Measure surface overlap within a tolerance margin; emphasizes boundary agreement.
	mAP [48] ↑	$\mathrm{mAP}=\frac{1}{N}\sum_{i=1}^{N}{\mathrm{AP}}_{i}$	Mean average precision, measuring the average precision across all classes.
	VOE [48] ↓	$\mathrm{VOE}=1-\frac{|A\cap B|}{|A\cup B|}$	Measure the volume mismatch between prediction and ground truth regions.
Detection	mAP [48] ↑	$\mathrm{mAP}=\frac{1}{N}\sum_{i=1}^{N}{\mathrm{AP}}_{i}$	Mean average precision, considering the precision at different recall levels.
	Recall [47] ↑	$\text{Recall}=\frac{\mathrm{TP}}{\mathrm{TP}+\mathrm{FN}}$	The ability to detect lesions.
	$F_{\beta }$ score [46]	$F_{\beta }=\frac{\left(1\;+\;\beta^{2}\right)\cdot \text{Precision}\cdot \text{Recall}}{\beta^{2}\cdot \text{Precision}}+\text{Recall}$	Weighted harmonic mean of precision and recall.
	FPPI [46] ↓	$\text{FPPI}=\frac{\text{Total}\;\text{FP}}{\text{Number}\;\text{of}\;\text{Images}}$	Number of false positives per image.
	FROC score [46] ↑	FROC = average sensitivity at predefined FPPI	The sensitivity of the model at different false positive rates.
	FPR [49] ↓	$\mathrm{FPR}=\frac{\mathrm{FP}}{\mathrm{FP}+\mathrm{TN}}$	The proportion of actual negatives that are incorrectly predicted as positive.
Registration	HD95 [49] ↓	$H\left(S_{f},\;S_{m}\right)=\text{max}\;\left(h\left(S_{f},\;S_{m}\right),\;h\left(S_{m},\;S_{f}\right)\right)$	Measure the similarity between the predicted and ground truth boundary.
	ASSD [48] ↓	$\text{ASSD}=\frac{\sum_{x\in X}\min_{y\in Y}d\left(x,\;y\right)+\sum_{y\in Y}\min_{x\in X}d\left(y,\;x\right)}{\mathrm{len}\left(X\right)+\mathrm{len}\left(Y\right)}$	Measure surface similarity between prediction and ground truth.
	MSE [50] ↓	$\mathrm{MSE}=\frac{1}{\mathrm{mn}}\sum_{i=1}^{m}\sum_{j=1}^{n}{\left(I_{\mathrm{ij}}-{\hat{I}}_{\mathrm{ij}}\right)}^{2}$	Measure average squared error between predictions and ground truth.
	SSIM [50] ↑	$S\left(A,\;B\right)=\frac{\left(2\mu_{A}\mu_{B}+\;c_{1}\right)\left(2\sigma_{\mathrm{AB}}+\;c_{2}\right)}{\left(\mu_{A}^{2}+\;\mu_{B}^{2}+\;c_{1}\right)\left(\sigma_{A}^{2}+\;\sigma_{B}^{2}+\;c_{2}\right)}$	Structural similarity index measure.
Reconstruction	PSNR [50] ↑	$\text{PSNR}=10\log_{10}\left(\frac{{\left(2^{k}-1\right)}^{2}}{\mathrm{MSE}}\right)$	Measure the difference between the original and reconstructed images.
	SSIM [50] ↑	$S\left(A,\;B\right)=\frac{\left(2\mu_{A}\mu_{B}+\;c_{1}\right)\left(2\sigma_{\mathrm{AB}}+\;c_{2}\right)}{\left(\mu_{A}^{2}+\;\mu_{B}^{2}+\;c_{1}\right)\left(\sigma_{A}^{2}+\;\sigma_{B}^{2}+\;c_{2}\right)}$	Evaluate image similarity in luminance, contrast, and structural.
	MSE [50] ↓	$\mathrm{MSE}=\frac{1}{\mathrm{mn}}\sum_{i=1}^{m}\sum_{j=1}^{n}{\left(I_{\mathrm{ij}}-{\hat{I}}_{\mathrm{ij}}\right)}^{2}$	Mean square error, which is sensitive to outliers.
Generation	MAE [50] ↓	$\mathrm{MAE}=\frac{1}{\mathrm{mn}}\sum_{i=1}^{m}\sum_{j=1}^{n}\left|\;,I_{\mathrm{ij}},-,{\hat{I}}_{\mathrm{ij}},\;\right|$	Mean absolute error.
	PSNR [50] ↑	$\text{PSNR}=10\log_{10}\left(\frac{{\left(2^{k}-1\right)}^{2}}{\mathrm{MSE}}\right)$	Peak signal-to-noise ratio.
	FID [51] ↓	$\mathrm{FID}=∥\;\mu_{r}-\mu_{g}\;{∥}_{2}^{2}+\mathrm{Tr}\left(\Sigma_{r}+\Sigma_{g}-2{\left(\Sigma_{r}\Sigma_{g}\right)}^{1/2}\right)$	Measure feature distribution differences between generated and real images.
	LPIPS [51] ↓	$\sum_{l}\frac{1}{H_{l}W_{l}}\sum_{h,w}{\left\|w_{l}⊙\left({\hat{f}}_{x,h,w}^{l}-\;{\hat{f}}_{y,h,w}^{l}\right)\right\|}_{2}^{2}$	Learned perceptual image patch similarity; perceptual distance in deep feature space.
	IS [51] ↑	$\text{IS}=\exp\left(E_{x}\;\mathrm{KL}\left(p\left(y|x\right)∥p\left(y\right)\right)\right)$	Measure image quality and diversity using a classifier.
	KID [189] ↓	$\mathrm{KID}=\left\|\;,\sum_{i\neq j}\frac{k\left(x_{i},\;x_{j}\right)}{m\left(m\;-\;1\right)}-\sum_{i,j}\frac{2k\left(x_{i},\;y_{j}\right)}{\mathrm{mn}}+\sum_{i\neq j}\frac{k\left(y_{i},\;y_{j}\right)}{n\left(n\;-\;1\right)}\right\|$	Kernel inception distance.
	CMMD [189] ↓	$\text{CMMD}\left(X_{s},\;X_{t}\;|Y\right)=\;∥\;E\left[\phi \left(X_{s}\right),\;,|,\;,Y\right]-E\left[\phi \left(X_{t}\right),\;,|,\;,Y\right]\;{∥}_{H}^{2}$	Conditional maximum mean discrepancy.
	SSIM [178] ↑	$S\left(A,\;B\right)=\frac{\left(2\mu_{A}\mu_{B}+\;c_{1}\right)\left(2\sigma_{\mathrm{AB}}+\;c_{2}\right)}{\left(\mu_{A}^{2}+\;\mu_{B}^{2}+\;c_{1}\right)\left(\sigma_{A}^{2}+\;\sigma_{B}^{2}+\;c_{2}\right)}$	Structural similarity index measure.
	TCT [190] ↑	$\mathrm{TCT}=\frac{|\;\mu_{\mathrm{wm}}-\mu_{\mathrm{gm}}\;|}{\sqrt{\sigma_{\mathrm{wm}}^{2}+\sigma_{\mathrm{gm}}^{2}}}$	The ratio between the inter-tissue intensity difference and the intra-tissue noise level.
Prognosis prediction and risk assessment	C-index [22] ↑	$C-\text{Index}=\frac{1}{M}\sum_{i:\sigma_{i}=1}\sum_{j:t_{i}<t_{j}}I\left({\hat{y}}_{i}\left(x_{i}\right)>{\hat{y}}_{j}\left(x_{j}\right)\right)$	Assess the survival models’ capacity to rank individual survival risks.
	AUC [48] ↑	$\mathrm{AUC}=\int_{0}^{1}\mathrm{TPR}\left(\mathrm{FPR}\right)\;d\left(\mathrm{FPR}\right)$	Reflect the model’s ability to distinguish between high- and low-risk patients.
	IBS [52] ↓	$\mathrm{IBS}=\frac{1}{\tau }\int_{0}^{\tau }\frac{1}{N}\sum_{i=1}^{N}{\left(S_{i}\left(t\right)-{\hat{S}}_{i}\left(t\right)\right)}^{2}\mathrm{dt}$	Evaluate the overall accuracy of predicted survival probabilities.

In classification and detection tasks, commonly used evaluation metrics include accuracy, recall, and specificity [47]. For segmentation tasks, Dice coefficient, intersection over union (IoU), and average symmetric surface distance (ASSD) are employed to assess the consistency between predicted regions and ground truth annotations [48,49]. In image reconstruction and registration tasks, metrics such as peak signal-to-noise ratio (PSNR), SSIM, and Hausdorff distance (HD95) are widely used to evaluate image quality and spatial alignment accuracy [49,50]. For image generation tasks, the Fréchet inception distance (FID) is commonly used to measure the distributional similarity between generated and real images [51]. In prognosis prediction and risk assessment tasks, commonly used metrics such as C-index, area under the ROC (receiver operating characteristic) curve (AUC), and integrated Brier score (IBS) evaluate models’ overall performance in survival probability prediction, risk stratification, and prognostic discrimination [22,52].

Although the aforementioned metrics are widely adopted in current research and offer good objectivity and reproducibility, they still fall short of fully reflecting the diagnostic value of models in real-world clinical settings. For instance, UniverSeg [53] evaluates segmentation performance solely using the Dice coefficient. Similarly, Swin-UMamba [54] employs the Dice similarity coefficient (DSC) and normalized surface distance (NSD) for evaluation. While these metrics effectively quantify the similarity between predictions and annotations, they remain insufficient in capturing clinically relevant aspects such as structural completeness, anatomical consistency, and generalization to complex or rare cases. Addressing these limitations represents a key challenge for current FMs. Future research should retain standard evaluation metrics while developing clinically aligned assessment frameworks to more comprehensively and realistically reflect a model’s value in actual diagnostic applications.

In summary, this section provides a systematic review of the typical tasks, commonly used datasets, and evaluation metrics in medical image interpretation. This not only establishes a unified framework for task definition and evaluation for subsequent research but also lays the foundation for model design and performance comparison. However, the diversity of medical image interpretation tasks and the complexity of the data characteristics impose differentiated requirements on the representational capabilities and learning paradigms of the models.

To address these requirements, research on FMs in medical imaging has primarily followed 2 technical paths, with the key distinction lying in the pretraining paradigms and data modalities employed. The first path focuses on VFMs that learn purely visual representations. These models leverage pretraining strategies such as supervised learning, contrastive learning, and generative learning to acquire generalizable visual features from large-scale imaging datasets, thereby significantly enhancing core visual interpretation tasks, including classification, segmentation, and detection [40]. The second path focuses on VLFMs that align visual and language semantics. These models rely on pretraining strategies such as masked modeling and cross-modal contrastive learning to achieve deep integration of medical images and text, not only improving performance on visual tasks but also enabling emerging multi-modal applications, such as Med-VQA and MRG [18].

The next section will first analyze the pretraining methods that drive the development of the aforementioned models, and then delve into the architectural innovations, representative works, and how VFMs and VLFMs address the diverse task requirements outlined in this section.

## Medical FMs

With the advancement of medical AI, research focus is gradually shifting from task-specific deep learning models toward FMs that can adapt to multi-modal and multi-task scenarios [16]. This section aims to systematically review the core methodologies and representative works underlying this paradigm shift, organized around 4 dimensions: pretraining models, VFMs, VLFMs, and extended multi-modal FMs. Finally, we provide a systematic analysis of the presented content to offer a clearer understanding of the developmental trends of medical FMs.

Specifically, Pretraining models focuses on pretraining models, which form the foundation of medical FMs. Their learning strategies and data modalities directly determine the representational capacity and transferability. This subsection systematically reviews vision pretraining and vision-language pretraining (VLP), further categorizing methods by learning paradigms, and summarizes their core objectives and representative approaches to establish a unified conceptual framework. Vision foundation models examines VFMs, highlighting the shift from single tasks or organ-specific applications toward cross-task and cross-modal generalization, with “model generality” as the key classification criterion [17]. Vision-language foundation models discusses VLFMs, emphasizing the suitability of encoder-only and encoder–decoder architectures for different medical image interpretation tasks, as well as their advances in cross-modal alignment and integration [19]. Finally, Extended multi-modal FMs extends to multi-modal fusion models, focusing on advances in integrating medical imaging with structured EHR data, physiological signals, and genomics, and indicating the potential trend of medical FMs toward more comprehensive intelligence.

In terms of specific classification, vision pretraining models focus on feature learning and transferability within the visual modality, serving as foundational representation methods [55], whereas visual FMs emphasize model generality, targeting cross-task and cross-modal applications. Similarly, VLP models primarily concern methods and techniques for joint vision-language pretraining [56], while VLFMs highlight cross-modal adaptability in medical image interpretation tasks. This classification logic clearly delineates the characteristics and applicable scenarios of different model categories, providing a systematic foundation for the in-depth analyses presented in the subsequent sections.

### Pretraining models

Pretraining in medical FMs aims to build generalizable and transferable representations by deeply exploring the latent structures and semantic associations within large-scale multi-modal medical data such as images, texts, and knowledge graphs [7]. This strategy addresses critical challenges in the medical domain, including limited annotations, heterogeneous modalities, and the long-tailed distribution of pathological features. The core methodology combines self-supervised and supervised learning. Self-supervised approaches extract low-level features such as anatomical structures and pathological patterns directly from raw data [57], while supervised learning incorporates expert annotations to embed medical prior knowledge, thereby enhancing interpretability and clinical decision-making. Compared to traditional training from scratch for each task, the pretraining paradigm significantly reduces the dependence on labeled data and improves model generalization and adaptability in low-resource settings [14].

After pretraining, the model undergoes fine-tuning to adapt general representations to specific clinical tasks [7]. Current fine-tuning strategies primarily include ZST, PEFT, and full-parameter fine-tuning (FPFT). ZST is designed to be directly applied to downstream tasks without additional model training. PEFT former freezes the backbone network and introduces lightweight modules, such as Adapter or LoRA [58], to enable rapid adaptation. In contrast, FPFT updates all parameters through end-to-end optimization, allowing the integration of multiple supervision signals to enhance performance [58]. The following sections focus on the vision and VLP models, and analyze their performance breakthroughs and medical adaptation strategies in key vision tasks, as summarized in Table 6.

**Table 6.** Summary of representative pretraining models in medical foundation models. The original publications or official sources of the datasets are provided in Table S4.

Category		Method	Public	Param	Vision encoder	Datasets	Adaptation
**Vision pretraining model**	Supervised learning	STU-Net [60]	[Arxiv’23]	14M–1.4B	nnU-Net	TotalSegmentator	FPFT
		MedSAM [27]	[Nature Communications’24]	-	ViT	1,570,263 image-mask pairs	ZST
		SegVol [62]	[NeurIPS’24]	180M	3D ViT/CLIP	25 Datasets, 96k CTs	ZST
	Contrastive learning	MoCo-CXR [64]	[PMLR’21]	-	ResNet18, DenseNet121	CheXpert	FPFT
		Endo-FM [65]	[MICCAI’23]	121M	Video Transformer	33K Video Clips	PEFT
		VoCo [63]	[CVPR’24]	62.2M	3D UNet, Swin-UNETR	1.6k CT Scans	FPFT
	Generative learning	Models Genesis [71]	[MIA’21]	16.32M	3D U-Net	LUNA16, ChestX-ray14	FPFT
		M^3^AE [68]	[AAAI’23]	-	3D U-Net	BraTS18, BraTS21	FPFT
		MIM-Med3D [69]	[WACV’23]	-	ViT, SwinTransformer, VAN	BTCV, BraTS21, TCIA-COVID19	FPFT
		FedMed-GAN [72]	[Neurocomputing’23]	-	GAN	IXI, BraTS21	FPFT
		Uni4Eye++ [70]	[TMI’24]	-	ViT	mmOphth-v2	FPFT
		Mim [55]	[TMI’25]	-	Swin Transformers	10,502 CT Scans	FPFT
		MedMAE [67]	[MDPI’25]	-	ViT	LUMID 2 Million+ Images	FPFT
	Predictive learning	Zhang et al. [73]	[MIA’24]	-	U-Net, AlexNet	CMR, Knee MRI	FPFT
	Hybrid learning	Swin UNETR [74]	[MICCAI’21]	62.2M	Swin Transformer	BraTS21	FPFT
		MIS-FM [75]	[arXiv’23]	-	CNN+Transformer	110k Unannotated 3D CT	FPFT
Category		Method	Public	Text encoder	Vision encoder	Datasets	Adaptation
**Vision-language pretraining model**	Mask modeling	MRM [78]	[ICLR’23]	Transformer	ViT	MIMIC-CXR	FPFT
		MedIM [79]	[MIA’24]	BioClinicalBERT	ViT	MIMIC-CXR-JPG	FPFT
	Contrastive learning	GLoRIA [80]	[ICCV’21]	BioClinicalBERT	ResNet-50	CheXpert, RSNA Pneumonia, SIIM Pneumothorax	ZST
		MedCLIP [4]	[EMNLP’22]	BioClinicalBERT	Swin Transformer	CheXpert, MIMIC-CXR, COVID, RSNA Pneumonia	ZST
		ConVIRT [81]	[PMLR’22]	BERT	ResNet50	MIMIC-CXR, 48k Musculoskeletal Image–text Pairs	ZST
		KAD [82]	[Nature Communications’23]	PubMedBERT	ResNet-50, ViT	MIMIC-CXR	ZST
		M-FLAG [83]	[MICCAI’23]	CXR-BERT	ResNet50	MIMIC-CXR	FPFT
		MITER [84]	[ESWA’23]	ClinicalBERT	ViT	900k Unlabeled Radiographs	FPFT
		Liu et al. [85]	[ACM MM’24]	BioClinicalBERT	ResNet-50	MIMIC-CXR	ZST
	Hybrid VLP	MedViLL [86]	[JBHI’22]	BERT	ResNet-50	MIMIC-CXR	FPFT
		MedKLIP [87]	[ICCV’23]	ClinicalBERT	ResNet-50	MIMIC-CXR v2	ZST
		MaCo [88]	[Nature Communications’24]	BERT	ViT	MIMIC-CXR v2	ZST
		MLIP [89]	[ISBI’24]	BioClinicalBert	ViT-B	MIMIC-CXR	ZST
		MM-Retinal [90]	[MICCAI’24]	BioClinicalBert	ResNet50	MM-Retinal, 4.3K image–text pairs	ZST
		MMCLIP [91]	[arXiv’24]	Transformer	ViT	MIMIC-CXR, PadChest	ZST

Public, methods reported in publicly accessible research papers and preprints; Param, number of trainable parameters; ZST, zero-shot transfer; PEFT, parameter-efficient fine-tuning; FPFT, full-parameter fine-tuning

Vision pretraining focuses on extracting generalizable pathological features from medical images, while VLP emphasizes cross-modal semantic fusion [59]. Both approaches leverage techniques such as self-supervised learning to reduce reliance on annotated data and incorporate domain knowledge to enhance clinical adaptability, thereby providing efficient and scalable FMs support for downstream tasks.

#### Vision pretraining models

In medical imaging, vision pretraining aims to develop models with generalizable visual representations by leveraging large-scale multi-modal medical images. These models are expected to automatically capture anatomical structures and latent pathological semantics, thereby improving performance in various downstream tasks, such as disease classification, lesion detection, and organ segmentation [55]. However, challenges remain due to data scarcity, privacy concerns, and inter-institutional variability. To address these issues, various learning paradigms have been proposed, primarily including supervised learning and self-supervised learning. Self-supervised learning can be further divided into contrastive, generative, predictive, and hybrid approaches. Collectively, these strategies drive medical vision models toward greater efficiency, robustness, and generalization.

##### Supervised learning

**Fig. 4.**
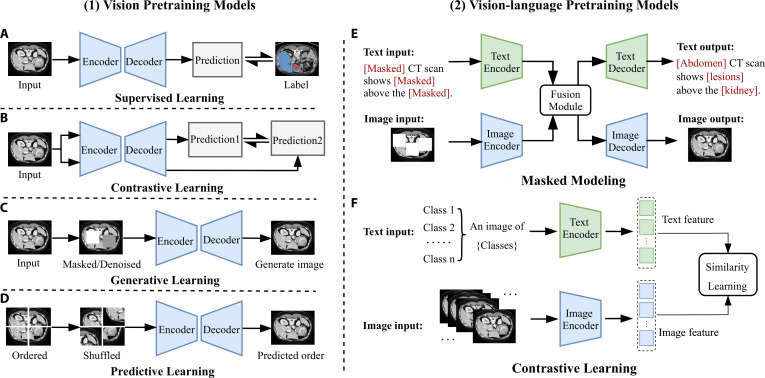
Overview of common pretraining paradigms for medical foundation models. Left: Vision pretraining approaches, including (A) supervised learning, (B) contrastive learning, (C) generative learning, and (D) predictive learning. Right: Vision-language pretraining paradigms, including (E) masked modeling and (F) contrastive learning.

Supervised learning trains models using paired image–label data, as illustrated in Fig. 4A, and is effective in learning image representations when annotated data are abundant.

For example, STU-Net [60], based on large-scale annotated datasets and a scalable architecture, validates the expressive power of supervised pretraining under both inference and fine-tuning settings. By systematically scaling network depth and width, STU-Net constructs a family of models ranging from 14 million to 1.4 billion parameters and is pretrained on the large-scale TotalSegmentator dataset. Experimental results show that model performance improves monotonically with increasing parameter size, and joint scaling of depth and width achieves optimal results—consistent with the scaling laws of VFMs. This study empirically validates the applicability of scaling laws to medical image segmentation and highlights the promising potential of large models for cross-modality and cross-task transfer [61]. However, its training is heavily dependent on large-scale annotated data, which limits adaptability to scenarios with scarce or unlabeled data.

MedSAM [27], the first universal segmentation model across multiple modalities and disease types, leverages large-scale multi-modal medical images to achieve unified representations of lesions and anatomical structures across 10 imaging modalities. However, this approach mainly depends on 2D image modeling and lacks direct capability for 3D volumetric medical data. SegVol [62] is a 3D foundation segmentation model based on semantic–spatial interactive prompts. By pretraining on 90,000 unlabeled CT scans and 6,000 labeled CT scans, together with the proposed “zoom in–zoom out” mechanism, it achieves efficient multi-scale fusion of semantic and spatial information. The model supports prompt-based segmentation of over 200 anatomical categories and demonstrates remarkable generality and interactivity across 22 anatomical segmentation tasks. Despite its superior performance in semantic prompt segmentation, SegVol still shows limitations in reference volume segmentation, which calls for optimization with more diverse prompt data in the future.

In summary, supervised pretraining on large-scale annotated datasets enables the effective learning of generalizable representations for medical image interpretation tasks. However, the high cost of medical image annotation and limited expert resources make it challenging to build large-scale, high-quality annotated datasets, thus limiting the scalability of supervised learning [57].

##### Contrastive learning

Contrastive learning aims to acquire discriminative image representations by minimizing the distance between positive pairs while maximizing the distance from negative pairs in the feature space [63]. It typically employs the information noise contrastive estimation (InfoNCE) loss function, which is defined as follows:

$$L_{\text{Image}}^{\text{InfoNCE}}=E_{\left(z_{i}^{I},\;z_{j}^{I}\right)}\left[-\log\frac{\exp\left(\mathrm{sim}\left(z_{i}^{I},\;z_{j}^{I}\right)/\tau \right)}{\sum_{k=1}^{N}\exp\left(\mathrm{sim}\left(z_{i}^{I},\;z_{k}^{I}\right)/\tau \right)}\right],$$ (1)

where $E_{\left(z_{i}^{I},\;z_{j}^{I}\right)}$ denotes the expectation over all positive sample pairs, and $\mathrm{sim}\left(z_{i}^{I},\;z_{j}^{I}\right)=\frac{<z_{i}^{I},\;z_{j}^{I}>}{∥\;z_{i}^{I}\;∥∥\;z_{j}^{I}\;∥}$represents the similarity measure, such as cosine similarity. τ is the temperature coefficient, and *N* is the total number of sample pairs including all negative samples. This loss function encourages the model to aggregate positive samples in the feature space while separating negative samples, thereby learning more discriminative image representations.

In medical image interpretation, contrastive learning has become a key technology for self-supervised pretraining, as illustrated in Fig. 4B. MoCo-CXR [64] builds upon the MoCo framework with targeted optimizations in initialization, data augmentation, and learning rate scheduling, enabling better adaptation to chest radiographs. Through fine-tuning on datasets of varying sizes and evaluation via linear classifiers and end-to-end training, it demonstrates enhanced representational quality and cross-task transferability, particularly under limited labeled data conditions.

Endo-FM [65] is the first FM for large-scale endoscopic videos. Built on a video Transformer with a teacher–student self-supervised learning strategy, it is pretrained on a endoscopy video corpus—over 33,000 video clips. By leveraging paired global and local spatiotemporal views that differ in spatial resolution and frame rate, Endo-FM learns resilient representations that remain stable across variations in viewpoint, scale, and motion. Moreover, it employs dynamic spatiotemporal positional encoding to handle inputs of diverse spatial sizes and temporal frequencies, achieving robust performance across tasks and extending the applicability of contrastive learning to dynamic medical video modeling. Endo-FM outperforms existing methods on various downstream tasks such as classification, segmentation, and detection, thereby extending the boundaries of contrastive learning applications in dynamic medical video modeling.

Moreover, VoCo [63] leverages inter-organ geometric consistency for volumetric contrastive self-supervised learning, allowing the model to learn consistent semantic representations without labeled data and exhibiting strong generalization capability. However, the scale and diversity of the datasets used for pretraining remain limited, which may constrain the model’s representational capacity. Further validation on larger and more diverse medical imaging datasets is therefore warranted.

##### Generative learning

Generative learning aims to capture high-level representations by reconstructing or generating image content. It typically uses pixel-level reconstruction losses to guide the model in restoring masked or perturbed regions, encouraging attention to local textures and critical anatomical details, as illustrated in Fig. 4C. Representative approaches include masked autoencoder (MAE) and its variants [66]. Among them, MedMAE [67] proposed a self-supervised pretraining backbone for medical images based on MAE, enabling the learning of generalizable and discriminative visual representations from medical images. Masked image modeling (MIM), as one of the core strategies in generative learning, usually reconstructs random image regions. Its loss function is the negative log-likelihood over the masked area, which measures the model’s ability to recover the missing content, defined as follows:

$$L_{\mathrm{MIM}}=-\frac{1}{|\;M\;|}\sum_{i\;\in \;M}\log\;f_{\theta }\left({\bar{x}}_{i}^{I}\;|{\hat{x}}_{i}^{I}\right),$$ (2)

where $L_{\mathrm{MIM}}$ represents the reconstruction loss, *M* denotes the set of masked voxels, and ${\bar{x}}_{i}^{I}$ and ${\hat{x}}_{i}^{I}$ correspond to the masked and unmasked patches in $x_{i}$, respectively.

M^3^AE [68] is a 2-stage, multi-modal self-supervised framework designed for brain tumor segmentation. It leverages a multi-modal MAE to achieve robust self-supervised learning under missing modalities and employs model inversion to efficiently optimize representative full-modality images, enabling a single model to handle all possible modality subsets and achieve high-performance segmentation in the presence of missing modalities. MIM-Med3D [69] systematically demonstrated the applicability and effectiveness of MIM for 3D medical images, accelerating downstream segmentation training and improving performance, while using a lightweight decoder to reduce computational cost. However, its representation capability and task adaptability under weakly labeled or limited supervision remain to be systematically evaluated. Mim [55] enhances feature representation through multi-granularity reconstruction and hierarchical alignment mechanisms. Mim learns discriminative features from multi-scale hierarchical visual representations through multi-granularity masked inputs, cross-layer alignment mechanisms, and a hybrid backbone network. It is pretrained on large-scale CT volumetric data, effectively capturing hierarchical anatomical features, and outperforms existing self-supervised learning methods on 12 public datasets for organ/tumor segmentation and disease classification. However, its current pretraining scale and downstream task coverage remain limited, and the potential for multi-modal integration and learnable hierarchical embeddings has yet to be fully explored, restricting its applicability to broader 3D medical imaging tasks. Uni4Eye++ [70] employs entropy-guided masked reconstruction to focus on information-rich regions and introduces a dynamic head generator to encode modality information and control reconstruction results, thereby alleviating modality confusion. It effectively unifies 2D and 3D ophthalmic image modeling under limited supervision and outperforms existing methods in multiple downstream tasks. Nevertheless, in the mmOphth-v2 dataset, there exists a significant imbalance between 2D images and 3D volumes, which may limit the effectiveness of the pretraining scheme. Additionally, to accommodate the pretraining model, 3D volumes are substantially downsampled, potentially affecting performance on high-resolution data.

In addition, other generative methods have been widely applied to medical image pretraining. For example, Models Genesis [71] aims to automatically learn universal anatomical representations from large-scale unlabeled 3D medical image. FedMed-GAN [72] combines federated learning with medical generative adversarial networks to address data privacy and decentralized training challenges in cross-modal brain image synthesis. It achieves superior performance under multi-modal and long-tailed data distributions while also significantly enhancing the quality of synthesized images. However, the generation effect of FedMed-GAN has not yet been verified by radiologists, and its interpretability needs to be further explored in the future.

##### Predictive learning

Predictive learning is a common paradigm in vision pretraining that aims to learn semantic structural features by predicting image attributes such as rotation angle, spatial position, or patch order [57]. These tasks typically focus on geometric or spatial relationships within the image, effectively guiding the model to capture spatial dependencies among different regions. This makes predictive learning particularly well suited for modeling anatomical structures in medical images, as illustrated in Fig. 4D. A common optimization objective is the cross-entropy loss, formulated as follows:

$$L_{\text{pred}}=-\sum_{i=1}^{C}y_{i}\log\left(f_{\theta }\left(x_{i}^{I}\right)\right),$$ (3)

where $x_{i}^{I}$ represents the input image and $f_{\theta }\left(x_{i}^{I}\right)\in {\mathbb{R}}^{C}$ represents the model’s predicted probability distribution. $y_{i}$ is the one-hot encoding of the true category, and *C* represents the number of categories. The loss quantifies the discrepancy between the predicted distribution and the ground-truth label.

A representative method by Zhang et al. [73] introduces 2 predictive tasks based on spatial relationships between imaging planes: learning the relative orientation between planes and regressing the relative position of the parallel planes within the image stack. This self-supervised pretraining strategy effectively guides the model to capture spatial representations aligned with organ structures, and its effectiveness has been validated on cardiac and knee medical images. However, the effectiveness of the self-supervised pretext task has only been evaluated in a transfer learning setting, and its applicability for direct pretraining and evaluation on the same dataset remains unexplored.

##### Hybrid learning

Hybrid learning improves model representation by integrating multiple pretraining strategies and leveraging the advantages of diverse tasks. Representative methods include Swin UNETR [74], which combines masked reconstruction, image rotation prediction, and contrastive learning for pretraining on large-scale unlabeled CT data. Its pretrained encoder provides robust feature extraction for downstream segmentation tasks. MIS-FM [75] employs a volume fusion self-supervised strategy that merges foreground and background subvolumes, enforcing the model to predict voxel-wise fusion coefficients, effectively converting the pretraining task into a pseudo-segmentation task and enhancing segmentation performance on 3D medical images and few-shot scenarios. Hybrid pretraining can more comprehensively model the texture details and spatial structures while taking into account both semantic understanding and structural reconstruction.

##### Comparative analysis of vision pretraining model

Based on the above analysis, different paradigms of medical vision pretraining exhibit distinct characteristics in their representation learning mechanisms and task adaptability. Supervised learning relies on large-scale annotated data to extract semantic information directly from medical images, enabling the learning of discriminative features highly relevant to downstream tasks. While it performs well when sufficient labels are available, its scalability is limited by the high cost of expert annotations [57]. In contrast, self-supervised learning designs pretext tasks such as image reconstruction, contrastive learning, and masked modeling to automatically learn generalizable representations from raw data, offering greater scalability. Contrastive learning emphasizes learning discriminative inter-sample relationships by pulling positive samples closer and pushing negatives apart in the feature space, which benefits classification tasks but depends on the quantity and quality of negative samples [63]. Generative learning reconstructs masked regions to guide the model in understanding both local structures and global semantics, excelling at fine-grained anatomical feature modeling for reconstruction and segmentation, although it may lack high-level semantic understanding [66]. Predictive learning focuses on predicting geometric or spatial properties to model anatomical structures, offering unique spatial understanding yet facing semantic gaps with complex downstream tasks [57]. Hybrid learning integrates multiple self-supervised objectives to balance semantic understanding, detail reconstruction, and structural modeling, achieving comprehensive and robust representations but facing challenges in optimization and computational cost. Overall, each paradigm offers unique advantages for building medical FMs, and their selection should balance data scale, annotation resources, computational budget, and task requirements.

In summary, these diverse pretraining strategies have collectively driven the paradigm shift from task-specific to generalized medical visual models. Medical vision pretraining is gradually becoming a key way to build FMs with multi-task transferability, structural awareness, and semantic understanding [14]. With the depth integration of multi-modal modeling, self-supervision learning, and federated learning, pretraining methods are expected to further overcome data barriers and task limitations, providing more universal, efficient, and trustworthy solutions for medical image analysis, accelerating the translation of intelligent medicine into clinical practice.

#### VLP models

VLP in medical image analysis aims to construct a cross-modal representation space by integrating anatomical structure and diagnostic knowledge embedded in medical language, thereby enhancing the model’s ability to understand critical regions and semantic relationships within medical images [56]. By leveraging large-scale medical image–text datasets, VLP enables the model to learn deep associations between anatomical structures and pathological features during training. This significantly improves robustness and cross-domain generalization in downstream tasks such as lesion detection and organ segmentation, particularly demonstrating superior performance over traditional vision pretraining approaches in data-scarce settings.

As the core of VLP, multi-modal representation learning aims to achieve semantic alignment and joint modeling between images and text [7]. Typical strategies include a shared representation space and feature alignment mechanisms.The shared representation space extracts features through separate image and text encoders and maps them into a common low-dimensional semantic space, where semantically related image–text pairs are brought closer together. This process is typically optimized using similarity metrics such as cosine similarity, supporting tasks such as image–text retrieval and generation. In contrast, feature alignment focuses on associating visual and language features at local or global levels. It employs cross-modal attention and hierarchical alignment strategies to enable fine-grained semantic mapping [76].

To achieve more effective cross-modal semantic alignment and joint modeling, medical VLP methods typically rely on multi-modal representation learning frameworks incorporating various self-supervised paradigms to capture deep correlations between medical images and text [14]. Among them, masked modeling, contrastive learning, and hybrid learning are the mainstream paradigms. These approaches enhance unified image text representation learning by focusing on different aspects such as image reconstruction and semantic alignment.

##### Masked modeling

Mask modeling is a general self-supervised learning paradigm that aims to learn contextual semantics and underlying structural information by randomly masking portions of the input, such as image patches or text tokens and predicting the original content of the masked regions [77], as illustrated in Fig. 4E. This strategy has been broadly applied in both natural language processing and computer vision tasks, enabling the extraction of implicit supervision signals from unlabeled data. Masked language modeling (MLM) is one of the earliest forms of masked modeling. In this task, certain words in an input sentence are randomly masked, and the model is trained to predict them based on contextual information, thereby learning language structure and contextual dependencies. The loss function is defined as follows:

$$L_{\mathrm{MLM}}=-\frac{1}{|M|}\sum_{i\in M}\log\;f_{\theta }\left({\bar{x}}_{i}^{T}|{\hat{x}}_{i}^{T}\right),$$ (4)

where *M* represents the set of masked positions, ${\bar{x}}_{i}^{T}$ represents the original token at position *i*, ${\hat{x}}_{i}^{T}$ represents the context information, and $f_{\theta }$ is a parameterized prediction function. Similarly, MIM extends this concept to the visual domain by masking portions of an image and predicting either the original pixel values or high-level semantic representations. This approach encourages the model to capture both local structures and global semantics [66]. The corresponding loss function is defined in Eq. (2).

Furthermore, mask cross-modal modeling extends the masking paradigm to multi-modal learning frameworks. It aims to reconstruct masked content through cross-modal information interaction when the modality is missing or incomplete. Its essence is also the generalization of the masked modeling paradigm in the multi-modal space [77]. The loss function is formulated as follows:

$$L_{\mathrm{MCM}}=-\frac{1}{|M|}\sum_{i\in M}\left[\log\;f_{\theta }\left({\bar{x}}_{i}^{I}|{\hat{x}}_{i}^{I}\right)+\log\;f_{\theta }\left({\bar{x}}_{i}^{T}|{\hat{x}}_{i}^{T}\right)\right].$$ (5)

Masked modeling has been widely used in medical image interpretation. For instance, MRM [78] preposes a masked record modeling framework that reconstructs masked image patches and masked report tokens, integrating visual information with medical knowledge during pretraining to learn knowledge-enhanced and highly generalizable semantic representations of chest x-rays (CXRs), achieving high performance and strong transferability on downstream classification tasks even under extremely low annotation conditions. MedIM [79] is the first medical MIM approach guided by radiology reports, which enhances semantic representations and downstream task performance through knowledge- and sentence-driven masking strategies. MedIM has only been studied on 2D medical images, and its applicability and effectiveness on 3D volumetric medical data have not yet been verified.

Overall, masked modeling, as a general and efficient self-supervised pretraining approach, has demonstrated excellent performance across various medical imaging scenarios and is gradually becoming a key technology for building FMs.

##### Contrastive learning

Contrastive learning in VLP facilitates cross-modal semantic alignment and discriminative representation learning by constructing positive and negative image–text pairs. It aims to maximize the similarity of semantically matched pairs in a shared embedding space while minimizing the similarity of unmatched pairs [57], as illustrated in Fig. 4F.

In the medical imaging domain, various methods have been proposed to fully exploit multi-modal information from images and reports. For instance, GLoRIA [80] proposes an attention-based multi-modal representation learning framework that contrasts image subregions with paired textual information, enabling joint learning of global and local features and improving performance on downstream medical image recognition tasks under limited or zero-shot label conditions. MedCLIP [4] decouples images and text for multi-modal contrastive learning, efficiently scaling up available training data, and introduces a medical knowledge-guided semantic matching loss to replace the conventional InfoNCE loss, effectively mitigating false negatives in contrastive learning. It demonstrates strong performance in zero-shot prediction, supervised classification, and image–text retrieval. However, it remains limited by semantic label noise, insufficient detection of negations or uncertain phrases, and the reliance of zero-shot predictions on prompt quality and pretraining data scale.

ConVIRT [81] pretrains medical image encoders via a bidirectional contrastive loss using naturally paired image–text data, enhancing generalization without expert annotations. ConVIRT is compared with ImageNet initialization, image caption-based initialization, and pure image contrastive learning methods, validating the data efficiency and effectiveness of image–text pretraining. However, it is not directly compared with subsequent methods that extend ConVIRT, such as GLoRIA [80].

KAD [82] incorporates medical domain knowledge into CXR VLP through knowledge graph and report entity extraction, and employs a disease query network to enable flexible zero-shot reasoning and interpretable predictions. Evaluation on 4 external CXR datasets shows that KAD’s zero-shot performance not only is comparable to fully supervised models but also exceeds the average performance of 3 radiologists in 5 pathologies, with statistical significance. M-FLAG [83] performs pretraining and regularization of medical VLMs using frozen language models and latent space geometry optimization, achieving efficient, parameter-efficient, and superior performance across multiple downstream tasks.

Similarly, MITER [84] presents a multi-level contrastive learning adaptive pretraining framework for medical image–text modeling, leveraging knowledge from large-scale datasets to enhance performance on multi-modal tasks and overcome small-sample limitations. Its effectiveness has been validated in image report retrieval, multi-label image classification, VQA, and report generation. MITER is suitable for downstream tasks that increasingly use text encoders rather than image encoders. Liu et al. [85] design a contrastive learning pretraining framework for patient-level data, employing a 2-stage feature extraction process to obtain more representative textual embeddings. Using a momentum encoder and memory queue, semantic exploration is conducted from cross-modal, multi-modal, and single-modal perspectives, fully utilizing potential information overlooked in prior studies. This work demonstrates significant improvements across zero-shot and data-efficient image classification, image segmentation, and cross-modal retrieval tasks.

##### Hybrid VLP

In recent years, medical hybrid VLP methods have gradually integrated multiple learning paradigms to enhance model understanding and generalization capabilities. For example, MedViLL [86] employs MLM and image report matching during pretraining, thereby maximizing the generalization performance of 3 types of vision-language understanding tasks: diagnosis classification, medical image report retrieval, and Med-VQA, and vision-language generation tasks for radiology report generation. MedKLIP [87] employs triplet extraction and entity-conversion encoding module to effectively mine rich domain knowledge in the medical field and implicitly model the relationships between medical entities in the language embedding space. By further integrating a Transformer-based spatial alignment strategy, MedKLIP achieves fine-grained alignment between visual patches and textual descriptions, leading to significant improvements in diagnostic performance on multiple CXR tasks.

Building on this line of research, several studies further explored the integration of masked modeling and contrastive learning to enhance fine-grained representation and zero-shot transferability. MaCo [88] employs masked contrastive learning to simultaneously achieve fine-grained image understanding and zero-shot learning while introducing a relevance-weighted mechanism to optimize the correspondence between masked CXR patches and their associated report segments, thereby enhancing the model’s representation ability. Although MaCo demonstrates strong performance on CXR tasks, its development is still limited in several ways. The limited scale and diversity of pretraining data hinder its generalizability across diverse clinical settings. In addition, the use of BERT for text encoding constrains the model from fully leveraging the capabilities of larger, domain-specific language models. Moreover, practical deployment still faces unresolved challenges, particularly concerning data privacy and ethical considerations. MLIP [89] leverages sentence-patch alignment to better utilize limited medical image–text data, and introduces a masked contrastive learning strategy guided by semantic completeness estimation, which reduces image redundancy while preserving key semantics. The method demonstrates significant improvements over existing approaches in zero-shot and few-shot classification and segmentation tasks. However, due to dataset limitations, MLIP currently focuses only on CXR image–text data.

In addition, Wu et al. [90] constructed the high-quality fundus image–text dataset MM-Retinal and introduced the pretraining model KeepFIT. By integrating domain-specific knowledge of fundus image–text analysis, and adopting image similarity-guided text rewriting together with a mixed training strategy, KeepFIT effectively injects clinical expertise, thereby enhancing both the generalization and transferability of fundus analysis tasks. MMCLIP [91] is a masked medical contrastive language-image pretraining framework that leverages the attention-masked image modeling (AttMIM) and entity-driven MLM module (EntMLM) modules, along with unpaired data and disease-type prompts, to enhance pathology-aware visual and textual representations, achieving strong zero-shot and fine-tuning performance across 5 datasets.

##### Comparative analysis of vision-language pretraining model

Based on the above analysis, different approaches to medical VLP exhibit distinct levels of hierarchy and complementarity in their cross-modal alignment mechanisms and task adaptability. Masked modeling encourages the model to learn cross-modal contextual dependencies and local semantic associations by reconstructing masked image regions or textual tokens [77]. This paradigm excels at capturing fine-grained anatomical structures and their correspondence to medical terminology but is relatively limited in achieving global semantic alignment. Contrastive learning, by constructing image–text positive and negative pairs and maximizing semantic consistency, effectively aligns visual and textual modalities at the global level, showing strong advantages in zero-shot classification and cross-modal retrieval tasks. However, its performance is sensitive to the quality of negative samples and often lacks fine-grained modeling of local semantic relations. To overcome the limitations of single paradigms, hybrid VLP integrates multiple objectives such as masked reconstruction and contrastive alignment, achieving a balance between global semantic consistency and local feature discrimination. This approach yields more comprehensive and robust cross-modal representations, substantially improving generalization in complex downstream tasks such as VQA and report generation. Nevertheless, hybrid methods introduce higher computational complexity and optimization challenges, and their reliance on large-scale, high-quality paired data remains a practical constraint [59]. Overall, the choice of pretraining strategy should consider the trade-off between global alignment and local understanding, data pairing quality, and computational resources to achieve optimal performance and feasibility.

In summary, VLP in medical imaging is advancing cross-modal semantic modeling through paradigms such as masked reconstruction, contrastive learning, and hybrid learning [56]. With the evolution of the representation learning and the introduction of medical knowledge such as medical knowledge graphs (MKGs) and structured reports, VLP models have demonstrated improved generalizability, interpretability, and clinical utility. Looking forward, VLP is expected to further strengthen knowledge alignment and semantic understanding while reducing dependence on manual annotations, thereby accelerating its practical applications in medical scenarios such as clinical decision support, disease prediction, and intelligent question answering.

#### Medical adaptation and fine-tuning strategies

**Fig. 5.**
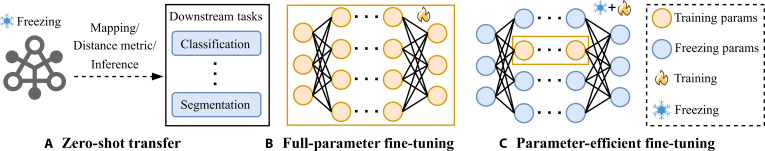
Comparison of transfer strategies from different pretraining models to downstream tasks, including (A) zero-shot transfer, (B) full-parameter fine-tuning, and (C) parameter-efficient fine-tuning.

Due to the characteristics of medical data, such as significant heterogeneity, high annotation costs, and stringent privacy restrictions, training models from scratch on target datasets is often computationally expensive and prone to overfitting [14]. As a result, how to effectively transfer pretraining models to specific medical scenarios has become a major research focus. Based on the amount of parameter updates required and the model adaptation methods for the target task, this section categorizes model transfer strategies into 3 approaches: ZST, FPFT, and PEFT (as illustrated in Fig. 5) and provides a systematic analysis of representative methods across various medical scenarios.

##### Zero-shot transfer

ZST refers to the direct application of large-scale pretraining models to downstream tasks without any additional task-specific training or fine-tuning. This capability typically relies on the model having acquired task-relevant general representations and reasoning abilities during the pretraining phase. Consequently, ZST is commonly observed in models based on large-scale supervised or multi-modal pretraining. For instance, supervised learning-based models such as MedSAM [27] and SegVol [62] trained on large-scale annotated segmentation datasets not only achieve state-of-the-art performance on known tasks and categories but also enable zero-shot segmentation of new datasets or novel classes through prompt engineering. In the realm of VLP models, methods including GLoRIA [80], MedCLIP [4], ConVIRT [81], KAD [82], Liu et al. [85], MedKLIP [87], MaCo [88], MLIP [89], MM-Retinal [90], and MMCLIP [91] facilitate zero-shot inference on downstream classification tasks via feature mapping or distance metrics. This strategy significantly reduces annotation requirements and lowers the barrier to deployment, thereby facilitating rapid validation of the model generalization capability. However, predictions are often coarse-grained and performance may be uncertain in specific scenarios or modalities.

##### Full-parameter fine-tuning

Full fine-tuning is one of the most widely adopted strategies in transfer learning, which involves end-to-end retraining of all parameters of a pretraining model on target medical task data to adapt it to specific downstream tasks. Unlike methods such as prompt learning or partial fine-tuning, full fine-tuning does not rely on specific input prompts or template designs but directly optimizes the entire model through gradient backpropagation. This approach is typically suitable for scenarios where the target domain data are relatively sufficient, computational resources are abundant, and there exists a significant domain gap between the pretraining and downstream tasks. For instance, vision pretraining models such as MoCo-CXR [64], VoCo [63], Models Genesis [71], M^3^AE [68], MIM-Med3D [67], FedMed-GAN [72], Uni4Eye++ [70], Mim [55], MedMAE [67], MIS-FM [75], and Zhang et al. [73] learn general representations of medical images but may lack explicit reasoning capabilities. Others, like Swin UNETR [74] and STU-Net [60], are constrained by domain shifts specific to medical imaging, while VLP models such as MRM [78], MedIM [79], M-FLAG [83], MITER [84], and MedViLL [86] also face challenges in direct downstream application. As a result, these models often depend on FPFT strategies. Although this approach often achieves optimal performance on specific tasks and adapts well to highly customized medical scenarios, it requires substantial computational and storage resources, is prone to overfitting on small-scale datasets, and hinders cross-task transfer and continuous learning.

##### Parameter-efficient fine-tuning

PEFT achieves task adaptation by introducing a small number of trainable parameters (e.g., adapters and low-rank factorization) while keeping the main body of the pretraining model frozen. This approach is suitable for medical scenarios with limited annotated data, large-scale models, or multiple tasks that share a common FM, such as Endo-FM [65]. It enables efficient adaptation to downstream tasks without significantly increasing computational costs. However, due to its inherent complexity and often decoupled design from the original pretraining architecture [65], PEFT methods are less commonly observed in the listed pretraining models. This strategy significantly reduces trainable parameters and computational resources while preserving model generality, making it favorable for multi-task or continual learning settings. Nevertheless, it may slightly underperform full fine-tuning on highly granular tasks (e.g., segmenting lesions with extremely fine structures) and can be sensitive to the design of prompts or adapters.

### Vision foundation models

VFMs are general models pretrained on large-scale visual data with cross-task transferability [17]. The concept originated in the field of computer vision, inspired by FMs in natural language processing, such as GPT [3]. The core of VFMs lies in powerful visual encoder architectures and leverages large-scale pretraining combined with self-supervised learning strategies [1], enabling them to learn hierarchical and semantically rich visual representations that can be efficiently adapted to a wide range of downstream tasks.

In recent years, VFMs have emerged prominently in medical image analysis, offering new solutions to key challenges such as few-shot learning, multi-modal integration, and task transfer [1,16]. To meet the complex demands of medical imaging, researchers have systematically classified existing VFMs into 3 typical paradigms based on their scope and domain characteristics: universal VFMs, modality-universal FMs, and task- or organ-universal FMs [17], as summarized in Table 7. This not only helps to systematically sort out the development of VFMs but also provides clear guidance for the application scenarios of different paradigms.

**Table 7.** Summary of representative medical vision foundation models. The original publications or official sources of the datasets are provided in Table S5.

Category	Method	Public	Param	Vision encoder	Data information	Task
Universal VFMs	Pai et al. [92]	[Nature machine intelligence’24]	-	3D ResNet50	11,467 radiographic lesions	Classification, prognosis prediction
	VFMGL [93]	[Nature Communications’25]	11M	ResNet18, U-Net	EC dataset ^a^, CAMELYON17, TNBC, I2CVB, PROMISE12, MoNuSAC2018, MoNuSAC2020, NCI-ISBI13	Classification, segmentation
	Foundation X [94]	[WACV’25]	-	Swin Transformer	11 Chest X-Ray Datasets	Classification, localization, segmentation
Modality-universal VFMs	UNI [95]	[Nature Medicine’24]	-	ViT	10w+ Diagnostic H&E-stained WSIs	Classification, quantitative evaluation
	Usfm [42]	[MIA‘24]	-	ViT-B	3M-US Database, 2187k+ US Images	Classification, segmentation, image enhancement
	LCTfound [96]	[medrxiv’25]	200M	U-Net+ViT	105,184 lung CT scans	Segmentation, diagnosis, prognosis prediction, generation, reconstruction, image enhancement, 3D modeling, treatment response prediction
Organ/task-universal VFMs	UniverSeg [53]	[ICCV’23]	1.18M	U-Net	MegaMedical	Segmentation
	SAMAug-C [26]	[ISBI’24]	-	SAM	ISIC 2017, Vitiligo, ExtCRC	Segmentation
	UniGradICON [21]	[MICCAI’24]	-	3D U-Net	COPDGenev, OAI, HCP, L2R-Abdomen	Registration
	VISTA3D [97]	[CVPR’25]	-	SegResNet	11,454 CT scans	Segmentation
	RETFound [30]	[NPJ digital medicine’23]	-	ViT	904,170 CFPs and 736,442 OCT scans	Detection
	PanDerm [98]	[Nature Medicine’25]	-	ViT-Large	2,149,706 unlabeled skin images	Segmentation, prognosis prediction, diagnosis, screening, risk assessment, change detection, ...

^a^ The dataset is private.

#### Universal VFMs

**Fig. 6.**
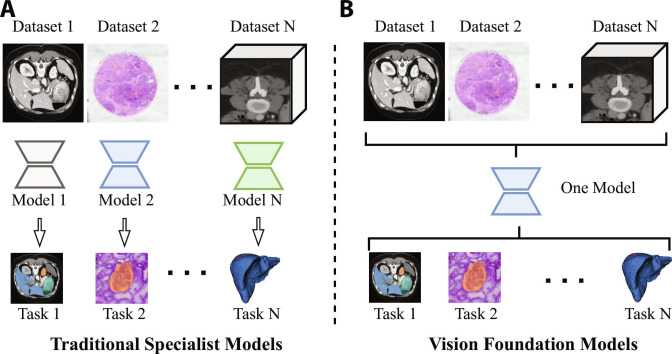
Comparison between traditional specialist models and vision foundation models, including (A) traditional specialist models and (B) vision foundation models.

Universal VFMs are typically pretrained on large-scale medical datasets using convolutional neural networks (CNNs) or vision transformers (ViTs) as backbone architecture, demonstrating strong cross-domain transferability [1]. Unlike traditional vision models that require task-specific network designs for individual medical tasks, universal vision models models employ a unified architecture that can be efficiently fine-tuned to adapt to diverse downstream tasks such as organ segmentation, lesion detection, and disease classification, as illustrated in Fig. 6.

Pai et al. [92] proposed an FM for cancer imaging biomarkers, pretrained through self-supervision on large-scale CT lesions, which supports multiple downstream tasks, including lesion anatomical classification, malignancy of lung nodule assessment, and non-small cell lung cancer tumor prognosis prediction, outperforming traditional supervised and existing pretrained approaches. The model’s reliability and reproducibility across different populations and tasks, as well as its robustness to distribution shifts, remain to be fully validated. In addition, according to scaling laws, expanding the pretraining dataset and increasing the model size could potentially further improve its performance. Moreover, the model’s “black-box” nature continues to constrain its interpretability and the transparency of its decision-making.

Many recent approaches have demonstrated superior performance. VFMsGL [93] introduces a heterogeneous-model general knowledge transfer (HGKT) method and screens low-heterogeneity data from each center through data deduction in batch level (DDBL) technology. This method combines the KD mechanism to effectively drive redundant parameters in local models and promote learning general knowledge from shared models. It is suitable for a variety of medical classification and segmentation tasks. However, the specific roles of adaptive features and general features in enhancing model robustness and generalization remain insufficiently explored. Foundation X [94] proposed a “lock-and-release” pretraining strategy and a “student–teacher” learning framework to enhance continual learning across multiple datasets. This approach preserves generalizable knowledge while mitigating overfitting, enabling robust performance across classification, localization, and segmentation tasks.

#### Modality-universal FMs

Different medical imaging modalities, such as CT, MRI, x-ray, and ultrasound, possess unique data distributions and visual characteristics [10]. Modality-universal FMs are pretrained on large-scale data from a single modality to effectively capture the unique textures and anatomical structures of that modality, enabling broad applicability across various tasks within it so that it is widely applicable to multiple tasks under this modality. These models emphasize cross-task generalization within the same imaging modality.

For instance, Usfm [42] is the first universal ultrasound FM, pretrained on a dataset comprising over 2 million multi-organ ultrasound images using a spatial-frequency dual-mask image modeling strategy. The model robustly extracts meaningful features from low-quality images and demonstrates excellent generalization and efficiency across multiple ultrasound tasks, including segmentation, classification, and image enhancement. Although Usfm performs strongly on 2D ultrasound image analysis, directly constructing a 3D ultrasound FM could better leverage the spatial relationships between images. Furthermore, due to computational constraints, the potential performance gains of larger model architectures, such as ViT-L, ViT-H, and Swin Transformer, remain to be explored. In the field of pathology, UNI [95] is a pathology universal FM pretrained in a self-supervised manner on over 100,000 hematoxylin and eosin (H&E)-stained whole slide images (WSIs). It outperforms existing methods across 34 CPath tasks and demonstrates novel capabilities, including resolution-independent tissue classification, whole slide imaging (WSI) classification with few-shot class prototypes, and generalization to disease subtypes.

LCTfound [96] combines diffusion model and self-supervised learning strategy, training on over 100,000 multi-center lung CT scans to achieve joint encoding of images and clinical data, achieving outstanding performance in multiple tasks, including lung tumor segmentation, disease diagnosis, and cancer prognosis. Currently, LCTfound is primarily pretrained on 2D images and has yet to integrate multi-modal information for more precise clinical diagnosis. Notably, with sufficient computational resources, a 3D lung CT FM could further enhance diagnostic capability for small lesions.

#### Task/organ-universal FMs

Task/organ-universal FMs focus on a certain type of clinical task or anatomical region and support a unified modeling strategy to support various downstream tasks within a given domain. Rather than aiming for full-domain generalization, they emphasize efficient knowledge transfer and strong generalization within targeted scopes. For example, UniverSeg [53] introduces query images and support sets of image–label pairs, leveraging a CrossBlock mechanism to jointly train across modalities and anatomical structures, enabling accurate segmentation at inference without retraining. UniverSeg is currently limited to 2D single-label segmentation and does not yet support 3D multi-label segmentation, which restricts its ability to model complex 3-dimensional structures. SAMAug-C [26] enhances classification datasets by generating variants of the original images using SAM and processes both the original and augmented images within a unified framework that integrates a deep learning classifier, SAMAug-C augmentation, and ensemble learning, effectively leveraging the complementary information from both inputs. UniGradICON [21] is the first universal FM for medical image registration, combining the generalizability of traditional methods with the efficiency of the deep registration network GradICON, and offering strong zero-shot performance across modalities and anatomical regions. However, UniGradICON is limited by the relatively small size of the current training dataset, the lack of training on multi-modal image data, and its current implementation solely based on the GradICON network. According to scaling laws, there remains potential to further enhance its performance by expanding the network size. VISTA3D [97] is the first FM that uniformly supports 3D automatic and interactive segmentation, combining a 3D supervoxel approach to extract pretraining 2D backbones for efficient correction and zero-shot capability. However, VISTA3D currently has limited support for categories and imaging modalities, and it does not yet include MRI and PET images.

In the domain of organ-universal FMs, RETFound [30] is the first large-scale retinal image FM trained via self-supervised learning. It learns generalizable representations from 1.6 million unlabeled retinal images and adapts them to labeled disease detection tasks, effectively supporting fundus disease diagnosis and systemic disease risk prediction. RETFound’s data sources are currently limited, primarily from the UK, and although it has evaluated the performance of CFP and optical coherence tomography (OCT) modalities, it has yet to explore multi-modal integration between them. PanDerm [98] is the first FM pretrained on multi-modal dermatology data, leveraging 4 imaging modalities and 2 million real clinical images. It achieves state-of-the-art performance across 28 downstream tasks, including skin cancer screening, risk stratification, differential diagnosis, lesion segmentation, longitudinal monitoring, and prognosis prediction. Nevertheless, PanDerm has limited coverage of rare genetic disorders, complex systemic diseases, and clinical variations, and its evaluation focuses on overall accuracy rather than disease-specific analysis; therefore, a more comprehensive evaluation framework is needed to assess its robustness.

In summary, visions are progressively establishing a universal framework for cross-modal and cross-task visual understanding [31]. As data scale grows, pretraining paradigms evolve, and medical knowledge integrates more effectively, VFMs are expected to play an increasingly central role in medical imaging, facilitating more intelligent, precise, and efficient healthcare services.

### Vision-language foundation models

VLFMs, a key branch of FMs, jointly model the cross-modal semantic relationships between medical images and text, providing a unified semantic understanding framework for clinical diagnosis [18,59]. These models have shown significant advantages in a variety of medical image interpretation tasks. The primary challenge lies in efficiently and accurately aligning visual features with textual semantics [76]. Current VLFM architectures focus on cross-modal feature fusion strategies and task adaptability to meet diverse clinical requirements.

In encoder design, VLFMs typically consist of a text encoder and a visual encoder that separately process medical text and imaging data [59]. Text encoders are usually Transformer-based, including medical variants such as BioBERT [99], which encode medical terminology and image descriptions into high-dimensional semantic vectors. Visual encoders extract semantic features from medical images using CNNs or ViTs. CNNs capture local spatial hierarchies through convolution and pooling, while ViTs divide images into fixed-size patches and model global dependencies via self-attention, effectively capturing complex relationships between lesions and anatomical structures. The text and visual encoders output vectors of matching dimensions, which are mapped into a shared semantic space through contrastive learning or similar methods, enabling strong image–text semantic alignment and enhancing multi-modal understanding and interpretability [76].

According to task objectives, VLFMs can be categorized into 2 types: encoder-only and encoder–decoder architectures, as summarized in Table 8. Encoder-only VLFMs focus on representation learning and semantic matching, making them well-suited for tasks such as Med-VQA and cross-modal retrieval. In contrast, encoder–decoder VLFMs further support pixel-level reconstruction and natural language generation, thereby better addressing complex generative tasks such as medical image segmentation and report generation. The following sections provide a systematic discussion of these 2 architectural paradigms.

**Table 8.** Summary of representative medical vision-language foundation models. The original publications or official sources of the datasets are provided in Table S6.

Category	Method	Public	Vision encoder	Text encoder	Data information	Task
Encoder-only	PubMedCLIP [100]	[EACL’23]	ViT,ResNet-50	CLIP	VQA-RAD, SLAKE	Med-VQA
	BioViL-T [101]	[CVPR’23]	CNN-Transformer	BERT	MIMIC-CXR v2, MS-CXR-T	Progression classification, phrase grounding, medical report generation
	MedBLIP [102]	[ACCV’24]	ViT-G/14	FLAN-T5,BioGPT, BioMedLM	30,000 3D Image Scans	Classification, Med-VQA
	ConceptCLIP [103]	[arXiv’25]	ViT	PubMedBERT	MedConcept-23M	Diagnosis, medical report generation, Med-VQA, text-to-image retrieval, image-to-text retrieval
Encoder–decoder	CLIP-Driven [29]	[ICCV’23]	-	CLIP	3,410 CT Scans	Segmentation, detection
	SAT [104]	[arXiv’23]	3D U-Net	BERT	22K 3D Medical Image Scans	Segmentation
	RoentGen [111]	[Nature Biomedical Engineering’24]	U-Net	CLIP ViT-L/14	MIMIC-CXR, CheXpert, VinDr-CXR	Generation
	Merlin [107]	[Research Square’24]	ResNet152	Clinical Longformer	1.5w+ CT Scans,180w+ EHRs Codes, 600w+ Report Tokens	Classification, segmentation, cross-modal retrieval, prognosis prediction, radiology report generation
	CRX-IRGen [110]	[WACV’24]	CLIP	CLIP	MIMIC-CXR	Generation
	CONCH [105]	[Nature Medicine’24]	ViT	-	1.17M Image-caption Pairs	Classification, segmentation, captioning, text-to-image retrieval, image-to-text retrieval
	BiomedGPT [76]	[Nature Medicine’24]	ViT	BERT	MIMIC-CXR, IU X-ray, PathVQA, VQA-RAD, PMC-OA	Classification, radiology report generation, radiology VQA, radiology report summarizes
	MUSK [109]	[Nature’25]	ViT	Transformer	TCGA, QUILT-1M, PathAsst	Image-to-text retrieval, text-to-image retrieval, Med-VQA, classification, molecular biomarker prediction
	BiomedParse [108]	[Nature Methods’25]	Focal21, SAM-ViT	PubMedBERT	BiomedParseData	Segmentation, detection, recognition
	FratMAE [106]	[arXiv’25]	ViT	BERT	AutoPET 3	Segmentation, Ann Arbor staging
	VOILA [28]	[AAAI’25]	-	CLIP	Totalsegmentator v2, WORD, AMOS, BTCV, AbdomenCT-1K, LiTS, Pancreas-CT	Segmentation
	RadFM [112]	[Nature Communication’25]	ViT	MedLLaMA-13B	MedMD, 13 million 2D Images and 615k 3D Scans	Diagnosis, Med-VQA, medical report generation

Note: The original publications or official sources of the datasets are provided in the supplementary database Table S6.

#### Encoder-only VLFMs

Encoder-only models generate fixed-dimensional multi-modal embeddings by jointly encoding visual and text features. These models are structurally simple and computationally efficient, making them well-suited efficient visual representation tasks such as VQA (Med-VQA) and medical cross-modal retrieval, although they lack sequence generation capabilities [19].

PubMedCLIP [100] adapts the CLIP model for the medical domain and achieves strong performance in VQA. Due to the lack of suitable data for training large-scale models, PubMedCLIP is currently limited to English Med-VQA, and different outcomes may be observed for other languages. BioViL-T [101] aligns cross-modal temporal semantics by integrating prior images and reports, achieving state-of-the-art performance across various vision-language tasks and validating its effectiveness on the CXR-T temporal benchmark. MedBLIP [102] integrates a pretrained image encoder with a large language model (LLM) via the MedQFormer module, bridging the gap between 3D medical images and 2D pretrained encoders and language models, and demonstrating strong zero-shot performance in Alzheimer’s diagnosis and Med-VQA. ConceptCLIP [103] is the first biomedical FM that combines high diagnostic accuracy with clinical interpretability. It achieves accurate and interpretable multi-modal medical image analysis through image–text–concept triples and dual alignment strategies.

#### Encoder–decoder VLFMs

In contrast, the encoder–decoder architecture decouple the processes of encoding and decoding, enabling an end-to-end workflow from visual representation to high-level semantic generation [19]. Such architectures demonstrate stronger transferability and generalization capacity, making them suitable for complex medical image interpretation tasks such as segmentation, MRG, and disease diagnosis. Nevertheless, they are limited by slower inference speed and a strong dependence of generation quality on the availability and quality of well-aligned medical data.

CLIP-driven universal model [29] incorporates CLIP-derived text embeddings into segmentation models to capture anatomical semantics. SAT [104] leverages multi-modal knowledge trees and contrastive learning to optimize the text encoder, and combines 3D U-Net and Transformer query decoder to achieve mapping text to segmentation masks. In line with the scaling law, expanding SAT from Nano (110 million parameters) to Pro (447 million parameters) led to significant improvements in performance, generalization, and robustness, achieving performance on par with 72 specialized nnU-Nets across 497 categories. CONCH [105] is the first large-scale vision-language contrastive pretraining model for tissue pathology. By unifying image and text modalities, it effectively improves multi-task generalization capabilities and performs well in tasks such as classification, segmentation, and cross-modal retrieval. FratMAE [106] employs separate ViT encoders for PET-CT and CT images and uses a cross-attention decoder to effectively integrate modality-specific information, further enhanced by textual metadata for cross-modal collaboration. FratMAE effectively captures the complex relationships among anatomical structures, metabolic activities, and textual features, demonstrating strong performance in lesion segmentation and Hodgkin’s lymphoma staging tasks, and showing its potential as a scalable PET/CT FM even with limited training data. However, despite its multi-modal architecture, its potential in single-modality applications for CT and PET remains underexplored. VOILA [28] introduces a voxel-language interaction framework and complexity-aware sampling (CAS) to achieve efficient voxel-level segmentation.

Merlin [107] is a VLFM for 3D CT, trained on a large-scale clinical dataset comprising 15,331 CT studies, over 1.8 million EHR diagnosis codes, and more than 6 million radiology report tokens, enabling diverse medical applications such as finding classification, cross-modal retrieval, outcome prediction, and 3D segmentation. BiomedParse [108] is a general biomedical FM trained on a large dataset comprising over 6 million triples of image, segmentation mask, and textual description, capable of jointly performing segmentation, detection, and recognition across 9 imaging modalities. Although BiomedParse performs well in pixel-level object recognition, it lacks instance-level differentiation and processes 3D modalities, such as CT and MRI, only as 2D slices, limiting its ability to leverage spatial and temporal information. MUSK [109] is a VLFM based on a multi-modal transformer, pretrained with unified masked modeling on 50 million pathology images and 1 billion text tokens, achieving efficient image–text alignment. It demonstrates strong performance across a wide range of downstream tasks, including image-to-text and text-to-image retrieval, Med-VQA, classification, and molecular biomarker prediction, as well as clinical prediction tasks such as melanoma recurrence in lung and gastroesophageal cancers, pan-cancer prognosis, and immunotherapy response prediction, thereby highlighting its potential in precision oncology.

CXR-IRGen [110] combines diffusion models and CAS to optimize the clinical consistency of CXR image and report generation. RoentGen [111] adapts latent diffusion models to the medical domain, leveraging CXR images and corresponding radiology reports to generate images that are both high-fidelity and conceptually accurate, while also serving as an effective data augmentation resource for downstream tasks. BiomedGPT [76] is the first open-source lightweight biomedical VLFM, built on a Transformer with a unified encoder–decoder architecture. Pretrained on large-scale biomedical image–text datasets, it achieves cross-modal alignment between vision and language and demonstrates state-of-the-art performance in classification, radiology report generation, radiology VQA, and radiology report summary, validating its generality and practicality for multi-modal biomedical applications. RadFM [112] is the first generalist FM for the field of radiology. It was pretrained on the MedMD dataset, which comprises 13 million 2D images and 615,000 3D scans, and then fine-tuned on RadMD—a domain-specific subset containing 3 million radiology-specific multi-modal samples. This model integrates natural language with 2D and 3D medical scans, utilizing natural language as output to address a wide range of medical tasks. Evaluation on the RadBench benchmark demonstrated that RadFM outperforms existing multi-modal FMs, including GPT-4V, in tasks such as diagnosis, Med-VQA, MRG, and rationale diagnosis.

**Fig. 7.**
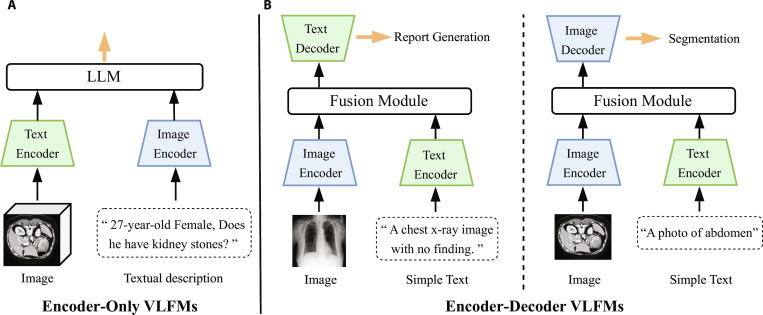
Structural classification of medical vision-language foundation models (VLFMs). (A) Encoder-only VLFMs. (B) Encoder–decoder VLFMs.

Overall, encoder-only architectures, owing to their structural simplicity and computational efficiency, are particularly well-suited for tasks such as VQA, medical image retrieval, and cross-modal alignment, where efficient feature extraction and representation are central, as illustrated in Fig. 7A. In contrast, encoder–decoder architectures achieve explicit mappings from low-level visual representations to high-level semantic generation through a decoding process, making them indispensable for complex tasks such as medical image segmentation and report generation, which require pixel-level reconstruction or natural language output, as shown in Fig. 7B. The differences in their design orientation and application scope highlight their complementarity in multi-modal medical tasks: The former emphasizes discriminative power and efficiency, while the latter prioritizes generative capability and flexibility.

### Extended multi-modal FMs

The previous subsection mainly reviewed the methods of FMs in medical image interpretation, with a particular focus on VFMs and VLFMs. However, the complexity of clinical data extends far beyond imaging, and recent studies have begun to explore the extension of FMs to additional modalities such as structured EHRs and continuous physiological signals, aiming to achieve more comprehensive clinical modeling and decision support [113]. This section focuses on the applications of FMs in structured EHRs, continuous physiological signals, and genomic data, as shown in Table 9.

**Table 9.** Summary of representative medical extended multi-modal foundation models. The original publications or official sources of the datasets are provided in Table S7.

Category	Method	Encoder	Data information	Task
Structured EHRs	EHRMamba [116]	Mamba	MIMIC-IV	Mortality, length of stay, readmission, hypertension, fluid disorders, lipoid metabolism disorders prediction
	Zhu et al. [117]	Transformer	1,288,333 patients with 587 million medical concept tokens from NYU Langone Health EHRs	The AD/ADRD/MCI risks in 1 and 5 years
	GDP [115]	Transformer	MIMIC-IV	Heart failure (HF), type 2 diabetes mellitus (T2DM), 30-d readmission prediction, discharge summaries generation
	MetaGP [119]	Transformer	8 million EHRs, biomedical literature, medical textbooks	Rare disease diagnosis, emergency conditions identification, medical report generation
	MsHeCare [118]	Transformer	eICU Dataset, Changde Dataset ^a^	Chronic disease predict (multi-label classification)
Physiological Signals	ECG-FM [122]	Transformer	PhysioNet 2021, MIMIC-IV-ECG	UHN-ECG interpretation, MIMIC-IV-ECG machine reads, UHN-ECG reduced LVEF
	Phukan et al. [123]	Transformer	WESAD	Physiological signal (ECG, EMG, EDA) classification and stress recognition
	PhysioPFM [124]	Transformer	Sleep-EDF dataset, DREAMER, MIT-BIH arrhythmia dataset, FOG dataset	Sleep-state detection, emotion detection, arrhythmia diagnosis, freezing of gaits detection
	PhysioOmni [121]	Transformer	TUEG, DEAP, Sleep-EDF dataset, CAP, GX	Emotion recognition, sleep stage classification, motor prediction, mental workload detection
Bioinformatics	scFoundation [126]	Transformer	Over 50 million human scRNA-seq data	Gene expression enhancement, tissue drug response prediction, single-cell perturbation prediction, single-cell drug response classification, cell type annotation, gene module inference
	scGPT [127]	Transformer	CELLxGENE	ScRNA-seq Integration with Batch Correction, Cell Type Annotation, Multi-omic Integration, Perturbation Prediction, Gene Regulatory Network Inference
	Nucleotide Transformer [125]	Transformer	3,202 human genomes, 850 genomes from diverse species	Predicting varied molecular phenotypes, promoter tasks, histone modification and enhancer tasks

^a^ The dataset is private.

#### Structured EHRs

EHRs provide patients’ longitudinal and comprehensive medical histories, encompassing structured data such as demographics, diagnoses, surgeries, vital signs, and medications, as well as unstructured clinical notes and medical imaging. With their widespread adoption in hospitals, EHR data have become important resources for supporting medical research and clinical applications such as personalized treatment plans, disease pattern discovery, rare disease identification, and clinical prediction [114]. However, EHR data are characterized by high dimensionality, longitudinal and heterogeneous, and irregular sampling, and are often accompanied by noise and missing values, posing significant challenges for traditional machine learning methods [115]. Recent advances in deep neural networks, particularly medical FMs, have shown remarkable ability to learn complex patient representations from multi-modal EHR data, thereby improving the accuracy of prediction tasks such as readmission prediction and disease identification, as well as enhancing clinical decision-making capabilities.

In this background, several representative EHR-based FMs have emerged and show a trend toward multi-modal integration. EHRMamba [116], built on the Mamba architecture and incorporating multi-task prompt fine-tuning with the HL7 FHIR standard, achieved state-of-the-art performance on 6 core clinical tasks in the MIMIC-IV dataset. These tasks include mortality prediction, length-of-stay prediction, readmission prediction, and the prediction of 3 specific diagnostic conditions: hypertension, fluid and electrolyte disorders, and lipid metabolism disorders.

Zhu et al. [117] trained an FM on the EHRs of 1.2 million patients and proposed the prediction model TRADE, which analyzes longitudinal visit records to effectively predict the risks of Alzheimer’s disease (AD/ADRD) and mild cognitive impairment (MCI) in individuals aged 65 and above. The model demonstrated robust capability in 1- and 5-year risk prediction, with a high positive predictive value in high-risk populations. This research significantly enhances the capability for early detection and risk assessment of dementia and provides a scalable solution for prognosis and management.

GDP [115] integrates structured EHR time series with unstructured clinical notes and employs an LLM backbone with cross-modal attention to derive unified latent representations. During generative pretraining, the model generates clinical narratives from structured data while capturing temporal dynamics. In the multi-task fine-tuning stage, it adapts to specific prediction tasks such as readmission and diagnosis prediction using labeled data. Unlike approaches that serialize EHR data into plain text, GDP consistently preserves multi-modal representations throughout the pipeline. On the MIMIC-IV dataset, it achieves breakthroughs in both clinical prediction and narrative generation, demonstrating strong performance in tasks such as heart failure, type 2 diabetes, and 30-d readmission prediction, while producing high-quality narratives that highlight its potential to improve patient prognosis and reduce documentation burden.

MsHeCare [118] employs a 2-stage framework. In the pretraining stage, it mitigates semantic bias in diagnostic and treatment sequences through self-supervised contrastive learning and MLM while leveraging cross-attention mechanisms to integrate temporal features with structured demographic information, thereby enhancing personalized patient representations. In the fine-tuning stage, it combines multi-source domain adaptation with source importance estimation to facilitate adaptive knowledge transfer across domains, improving model generalization. Experiments demonstrate that this method significantly outperforms existing approaches in chronic disease prediction on real-world EHR data, showing strong robustness and clinical consistency.

It is noteworthy that these methods primarily focus on structured and unstructured EHRs modalities, whereas MetaGP [119] further incorporates medical imaging in downstream tasks, demonstrating the potential for cross-modal extension.

MetaGP is built upon the Qwen-1.5 32B architecture and pretrained on more than 8 million EHRs, biomedical research articles, and medical textbooks. Through data aggregation, tokenization, Qwen model pretraining, and continued pretraining, MetaGP effectively handles long-context data while encoding complex medical knowledge. During the fine-tuning, MetaGP incorporated approximately 630,000 rare disease and emergency EHRs, over 600,000 CXRs, and nearly 24,000 CT scans, alongside natural language data and diverse natural language datasets, including medical question-answering dataset such as PubMedQA, MedQA, and MedMCQA, as well as dialogue and reasoning resources such as the OpenAssistant dataset and the chain-of-thought collection. Experimental results demonstrate that MetaGP achieves the performance of both clinicians and GPT-4 in rare disease diagnosis, emergency situation identification, and MRG, showing its potential for improving diagnostic accuracy and alleviating clinical documentation burden. However, according to the scaling law, the relatively small size of MetaGP may limit its ability to encode and retain large-scale medical knowledge.

Overall, EHR-based medical FMs, by learning generalizable and hierarchical data representations, not only improve the accuracy and generalizability of clinical prediction tasks but also enable cross-modal information integration. The combination of medical imaging and EHR data holds the potential to construct more comprehensive patient representations, further enhancing predictive performance and clinical decision support.

#### Physiological signals

Continuous physiological signals refer to dynamic health-related data continuously recorded by medical devices or wearable sensors, such as EEG, ECG, electromyogram (EMG), galvanic skin response (GSR), cardiotocography (CTG), and electrooculogram (EOG) [120]. Unlike medical imaging focused on spatial structure, continuous physiological signals highlight temporal dynamics. Represented as time series, these signals capture the dynamic variations of physiological activities, making them well-suited to track disease progression or instantaneous fluctuations in patient status. FMs based on physiological signals leverage large-scale pretraining to learn cross-modal general representations, enabling efficient transfer and generalization across downstream tasks such as emotion recognition, sleep staging, and disease classification, while maintaining robustness under data scarcity or imbalance [121].

In this direction, ECG-FM [122] demonstrates an open ECG FM based on the Transformer architecture. Pretrained on 1.5 million ECG recordings using a hybrid contrastive and generative self-supervised strategy, it demonstrates strong performance across 3 clinically relevant tasks: (a) ECG interpretation on the UHN-ECG dataset using pattern matching and knowledge graph-based label refinement; (b) automated ECG report interpretation on MIMIC-IV-ECG by adapting the parsing system to standardized terminology and constructing consistent binary labels; and (c) left ventricular ejection fraction (LVEF) prediction on UHN-ECG by extracting threshold labels from ECG reports for early heart failure screening. Results show that ECG-FM achieves robustness, label efficiency, and strong discriminative power, significantly outperforming task-specific models across datasets.

Phukan et al. [123] conducted the first systematic evaluation of state-of-the-art speech foundation models (SFMs), including WavLM, Wav2vec2, UniSpeech-SAT, x-vector, HuBERT, MMS, XLS-R, and Whisper, on physiological signal classification [ECG, EMG, ectrodermal activity (EDA)] and stress recognition. Despite being pretrained only on speech data, SFM representations consistently outperformed models trained directly on raw physiological data, with multilingual SFMs achieving the best performance, highlighting their strong cross-domain generalization ability.

To enhance personalization and privacy protection, PhysioPFM [124] introduces a personalizing time series foundation model (TSFM) for physiological signals, designed to generate personalized models without additional training during deployment. PhysioPFM is pretrained on large-scale public physiological datasets via low-rank adapters (LoRA) and constructing data mappings while mitigating class imbalance through neural folding. During the generator stage, diffusion transformers (DiT) synthesize LoRA weights conditioned on time series shapelets, bridging the gap between temporal data and adapter parameters. During local personalized inference, DiT generates personalized LoRA weights based on the provided shapelets and integrates them with the universal TSFM, enabling efficient adaptation to clinical tasks such as sleep-state detection, emotion recognition, arrhythmia diagnosis, and freezing of gait detection, while preserving performance and data privacy.

In more complex multi-modal scenarios, PhysioOmni [121] models both homogeneous and heterogeneous features, employing a decoupled multi-modal tokenizer to disentangle signals into modality-invariant and modality-specific representations and leveraging masked signal modeling to learn cross-modal general representations. During fine-tuning, a homogeneous representation mapping projects features from different modalities into a unified space, combined with prototype alignment and modality-specific prediction to ensure robust adaptability across arbitrary modality combinations. Evaluations on 4 brain–computer interface (BCI) tasks—emotion recognition, sleep staging, motion prediction, and workload detection—demonstrate state-of-the-art performance, validating the effectiveness of decoupled multi-modal learning and elastic fine-tuning.

In summary, current FMs for continuous physiological signals primarily focus on multi-modal time-series data, with limited exploration of integration with medical imaging. Unifying physiological signals and imaging holds promise for building more comprehensive patient representations, enhancing clinical prediction, supporting personalized care.

#### Bioinformatics

Advances in bioinformatics have created new opportunities to unravel the complexity of biological systems, spanning multi-modal omics layers such as genomics, transcriptomics, and proteomics. A central goal is to elucidate the molecular mechanisms of disease and to facilitate precision medicine applications, including disease risk prediction, targeted cancer therapies, and drug development [113]. Despite these advances, the rapid expansion of multi-modal omics data poses a major challenge for effective integration and utilization. Medical FMs enable these omics data to improve molecular-level tasks such as disease diagnosis, genetic disorder screening, and antigen–antibody recognition, thereby accelerating the implementation of personalized medicine.

In the field of genomics, Nucleotide Transformer [125] is a large-scale pretrained FM with 50 million to 2.5 billion parameters. It integrates genomic information from 3,202 human genomes and 850 genomes of diverse species. By generating context-specific representations of nucleotide sequences, Nucleotide Transformer can capture key genomic elements even in low-data settings and can be efficiently fine-tuned to support a variety of genomic applications, including improving the prioritization of genetic variants.

In the field of transcriptomics, scFoundation [126] is an FM designed for single-cell transcriptomics data. The model comprises 100 million parameters, spans approximately 20,000 genes, and is pretrained on more than 50 million human single-cell transcriptomic profiles. Its asymmetric transformer architecture and designed pretraining tasks enable effective modeling of complex contextual relationships among genes across diverse cell types and states. It demonstrate that scFoundation achieves state-of-the-art performance across a wide range of single-cell analysis tasks, including gene expression enhancement, tissue drug response prediction, single-cell drug response classification, single-cell perturbation prediction, cell type annotation, and gene module inference. However, scFoundation focuses solely on transcriptomic data without incorporating genomic or epigenomic information. Moreover, although unsupervised pretraining avoids reliance on large-scale manual annotations, it overlooks the rich information embedded in metadata. Integrating cellular metadata with transcriptomic data could better link molecular features to phenotypes.

Meanwhile, single-cell RNA sequencing (scRNA-seq) enables fine-grained characterization of cellular heterogeneity, supporting advances in lineage tracing, disease mechanism elucidation, and personalized medicine, while generating large-scale resources such as the Human Cell Atlas. Efficiently leveraging the rapidly growing multi-modal sequencing data remains a major challenge. scGPT [127] addresses this by performing generative pretraining on over 33 million cells, establishing a unified framework for nonsequential omics data, and optimizing the Transformer architecture to jointly learn gene and cell representations. This approach achieves state-of-the-art performance across downstream tasks, including cell type annotation, multi-batch and multi-omics integration, perturbation response prediction, and gene network inference.

FMs have shown strong potential across genomics, transcriptomics, and single-cell omics [113]. However, due to the distinct characteristics of each field, research remains largely confined to individual modalities, with limited integration with phenotypic data such as medical imaging. Future efforts in cross-modal modeling could enable multi-scale integration from molecules to organs, systematically revealing disease mechanisms and advancing precision medicine.

### Comparative analysis of medical FMs

#### Comparison of VFMs and VLFMs

To comprehensively present the advantages and limitations of medical VFMs and medical VLFMs across various dimensions, we systematically compare them in terms of input modalities, model architectures, applicable tasks, generative capability, and interactivity, as shown in Table 10. With the increasing diversity of medical imaging data and the growing complexity of tasks, both VFMs and VLFMs have demonstrated complementary strengths in the medical domain. VFMs excel in medical image representation and discriminative tasks, showing strong feature extraction and transfer capabilities, particularly in segmentation, detection, and diagnostic prediction, where they offer high stability and interpretability [1]. However, they exhibit significant limitations in cross-modal understanding, textual interaction, and generative tasks, struggling to meet the demands of multi-modal information integration and complex clinical semantic reasoning. In contrast, VLFMs enable multi-modal knowledge alignment through joint modeling of images and texts, demonstrating greater flexibility and generalization in ZST, interactive reasoning, and report generation. However, they face challenges such as the scarcity of medical annotations, limited textual corpora, and difficulties in cross-language adaptation [18]. Overall, VFMs hold advantages in structured, high-precision medical tasks, while VLFMs show greater potential in multi-modal, interactive, and generative medical applications. The integration of both approaches is expected to drive the development of next-generation medical intelligent systems.

**Table 10.** A comprehensive comparison of vision foundation models and vision-language foundation models

Method	Modal	Structure	Application tasks	Generative capability	Interactivity
Universal VFMs	Multi-modal	Encoder–decoder	Segmentation, detection, classification	✓	✓
Modality-universal VFMs	Uni-modal	Encoder–decoder	Segmentation, detection, classification	**✗**	**✗**
Organ/task-universal VFMs	Multi-modal	Encoder–decoder	Segmentation, detection, classification	**✗**	**✗**
Encoder-only VLFMs	Multi-modal	Encoder-only	Classification, Med-VQA, report generation, diagnosis	**✗**	**✗**
Encoder–decoder VLFMs	Multi-modal	Encoder–decoder	Segmentation, detection, diagnosis	√	√

#### Temporal analysis of medical FMs

**Fig. 8.**
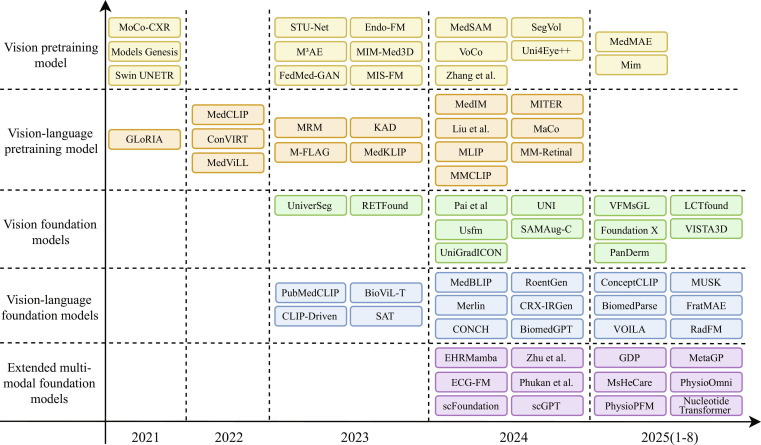
The time-research paradigm of the medical foundation models approach.

As illustrated in Fig. 8, we systematically categorized the methods discussed in this section along the temporal dimension and research paradigm categories in order to better understand the longitudinal evolution of the field. Overall, the research focus exhibits relatively stable, stage-wise development.

During 2021–2022, studies primarily concentrated on vision pretraining and VLP paradigms, with the central objective of learning robust representations from medical images—particularly in the context of limited annotations—to support downstream interpretation tasks such as segmentation and classification. Beginning in 2023, with the rapid advancement of large-scale pretraining and general-purpose architectures, the research focus gradually shifted toward vision and VLMs framed explicitly as FMs [1,76]. At this stage, attention extended beyond representation learning itself toward stronger task adaptability and transferability. Entering 2024 and beyond, a clear trend toward multi-modal medical data integration can be observed, encompassing EHRs, continuous physiological signals, and genomics [113]. This indicates that researchers have begun to explore extending the capabilities of FMs to broader and more complex clinical data domains.

In summary, medical FMs demonstrate significant potential in pretraining models, VFMs, VLFMs, and extended multi-modal FMs. However, existing studies still face challenges such as insufficient task-level collaboration, fragmented training paradigms, and limited model deployment efficiency. These limitations, to some extent, hinder the widespread adoption and application of medical FMs in real clinical settings. To systematically address these challenges and bridge the gap between research and practical application, there is an urgent need to develop an open-source, integrated, and modular medical FM platform. Based on this motivation, in the next section, we propose and introduce the IPIU medical FM platform, designed to achieve these objectives and support subsequent objective evaluation and experimental validation.

#### Scaling laws in medical FMs

In recent years, scaling laws have become an important topic in FMs research. By revealing power–law relationships between model performance, model scale, data scale, and computational resources, they provide a theoretical basis for performance prediction and resource planning [128]. The core idea is to fit a power–law curve describing how the loss function changes with scale, based on training experiments conducted on models and datasets of varying sizes. While preliminary scaling patterns have been established in natural language and general vision domains [61], their applicability in the medical field faces unique challenges. Medical data are typically scarce in annotations and highly heterogeneous, leading to nonmonotonic performance scaling and a stronger dependence on the trade-off between data diversity and computational resources.

Therefore, in the medical context, discussions of scaling laws needs to shift from the “single pursuit of larger models/more data" to a balance among the 3 dimensions of “data scale–model scale–computational budget”. Previous studies have shown that as model scale, computational scale, and data scale increase, pretraining loss and downstream task performance steadily improve [61]. This trend has also been preliminarily validated in medical FMs. For example, Zhang et al. [129] conducted the first study on scaling laws for EHR-based FMs. Training Transformer models of different sizes on the MIMIC-IV dataset, they identified consistent scaling patterns, including a parabolic IsoFLOPs curve and power–law relationships between computation, model parameters, data scale, and clinical utility. This suggests that medical FMs exhibit predictability and efficiency when scaled.

In the context of scarce medical data and high annotation costs, the core of the compute-optimal problem shifts from “how to allocate computation to scale models and data” to “how to achieve optimal performance with limited data”. For example, OptiDEL [130] achieves a 6.2% higher mIoU using only 5% of the pretraining data compared to models trained on the full dataset. While improving performance by an average of 4.7%, it reduces the training data requirement to ^1^/_20_ of the original, highlighting the importance of effective sample utilization under limited resources.

In small-sample medical scenarios, the suitability of different pretraining paradigms requires a comprehensive consideration of task consistency and data characteristics. Generative pretraining learns the intrinsic structure of the data through masked reconstruction. While it requires substantial computational resources, it excels in generalization for downstream tasks, making it suitable for diverse tasks with large-scale, unlabeled data. Contrastive learning constructs a discriminative feature space by bringing positive sample pairs closer, and is particularly suitable for tasks like cross-modal retrieval. Its computational efficiency is closely related to task consistency, but it requires high-quality, large-scale paired data. Selecting an appropriate pretraining paradigm based on task requirements and data conditions is crucial for improving scaling effectiveness.

Despite preliminary explorations, systematic research on scaling laws for medical FMs remains limited, particularly in cross-modal validation and real clinical applications, which is still in its early stages. Constrained by issues such as data sharing and standardization, unified evaluation and analysis of scaling across modalities or institutions is lacking. With the advancement of federated learning and medical image generation technologies [13,35], future work is expected to enable more systematic cross-institutional validation and performance comparisons, demonstrating greater potential for performance enhancement, resource optimization, and clinical translation.

## IPIU Medical FM Platform

With the widespread application of AI in the medical domain, building a unified, efficient, and scalable medical AI platform has become a core requirement for advancing intelligent healthcare. Currently, medical FMs are emerging as a vital bridge connecting general AI capabilities with specific clinical tasks, and their importance is increasingly recognized [15]. In particular, with the continuous accumulation of multi-modal medical data such as CT, MRI, pathology images, and clinical texts, developing traditional models designed for single tasks or single modalities is no longer sufficient to meet the diverse needs of complex clinical environments.

**Fig. 9.**
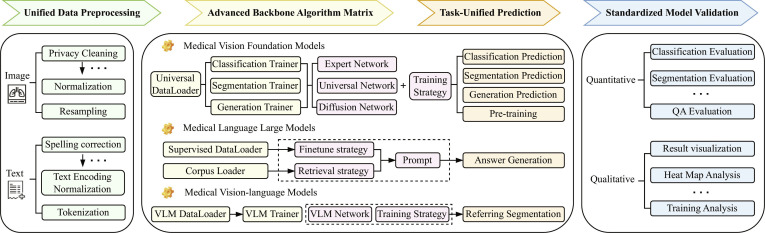
Framework of the IPIU medical foundation model platform. This platform provides a complete set of foundation model construction processes: unified data pre-processing, advanced backbone algorithm matrix, task-unified model prediction, and standardized model verification.

Therefore, there is an urgent need for an integrated and modular platform that supports multi-modal and multi-task collaborative training and efficient deployment, thereby accelerating the development and clinical translation of medical FMs. To this end, we propose the IPIU medical FM platform, which aims to provide state-of-the-art model architectures, fully reproducible training paradigms, and comprehensive pretraining weights for rapid adaptation to downstream medical applications, as illustrated in Fig. 9. The unique technical contribution of the IPIU platform lies in its effective integration of nnU-Net’s [8] pipeline robustness with MONAI’s [131] modular flexibility, ensuring training stability and reproducibility while providing highly scalable model interfaces and multi-task support capabilities. Its main contributions include the following: first, the introduction of a systematic training framework for medical VFMs, supporting large-scale pretraining and validation; second, the implementation of flexible integration and unified modeling for multiple task types (e.g., segmentation, classification, and detection); and third, the extension of language and multi-modal processing capabilities on top of visual models, providing a systematic foundation for building comprehensive medical intelligence analysis platforms. These features endow the IPIU platform with significant methodological and engineering advantages in the development and clinical translation of medical FMs.

### Large-scale multi-modal medical datasets

The platform incorporates 12 commonly used medical imaging modalities, including MRI, CT, PET-CT [44], computed tomography angiography (CTA), intraoral scanning (IOS), x-ray radiography (x-ray) [41], ultrasound imaging (US), endoscopy [10], WSI/pathology, OCT, OCT angiography (OCTA), and fundus imaging.

Among these modalities, MRI includes various imaging sequences, such as T1-weighted (T1), contrast-enhanced T1-weighted (T1C), T2-weighted (T2), and fluid-attenuated inversion recovery (FLAIR), each providing distinct tissue contrast characteristics [44].

**Fig. 10.**
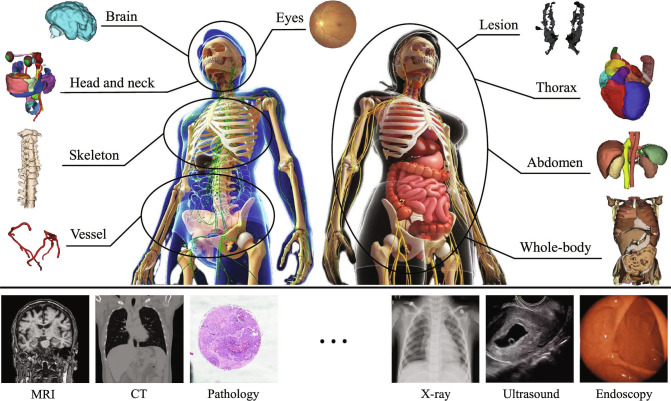
IPIU large-scale multi-modal medical image dataset.

The platform categorizes the datasets into 9 groups based on anatomical regions and clinical applications: whole-body multi-organ, brain, eye, head and neck, thorax, abdomen, vessel, and skeleton, as illustrated in Fig. 10. In total, over 130 publicly available datasets have been integrated, comprising 4.4w+ 3D images and 21w+ 2D images, covering a range of tasks including classification, segmentation, detection, registration, reconstruction, and generation. Tables 11 to 19 provide a systematic summary of each dataset’s modality, task, data format, target, data size, and sources.

**Table 11.** Summary of whole-body dataset within the IPIU large-scale medical imaging dataset

Dimensions	Modality	Dataset	Task	Targets	Data size	File format
3D	CT	Total Segmentator v1.0	Organ segmentation	104	1,204	nii.gz
		Total Segmentator v2.0	Organ segmentation	117	1,228	nii.gz
		CT-ORG	Organ segmentation	6	140	nii.gz
	PET-CT	AutoPET 1 (2022)	Lesion segmentation	1	1,164	nii.gz
		AutoPET 2 (2023)	Lesion segmentation	1	1,214	nii.gz
		AutoPET 3 (2024)	Lesion segmentation	1	1,614	nii.gz
	MRI	TotalSegmentator MRI	Organ segmentation	56	298	nii.gz

**Table 12.** Summary of brain dataset within the IPIU large-scale medical imaging dataset

Dimensions	Modality	Dataset	Task	Targets	Data size	File format
3D	CT	INSTANCE 2022	Lesion segmentation	1	200	nii.gz
	MRI	FeTA 2022	Organ segmentation	7	280	nii.gz
		iSeg	Organ segmentation	3	23	nii.gz
		cSeg 2022	Organ segmentation	3	13	nii.gz
		CAS2023	Organ segmentation	1	100	nii.gz
		BraTS21	Lesion segmentation	3	2,040	nii.gz
		BraTS2023-SSA	Lesion segmentation	3	105	nii.gz
		BraTS2023-MET	Lesion segmentation	3	328	nii.gz
		BraTS2023-MEN	Lesion segmentation	3	1,650	nii.gz
		BraTS2023-PED	Lesion segmentation	3	228	nii.gz
		BraTS-TCGA-LGG	Lesion segmentation	3	65	nii.gz
		BraTS-TCGA-GBM	Lesion segmentation	3	102	nii.gz
		MSD Brain (Task01)	Lesion segmentation	3	750	nii.gz
		MSD Hippocampus (Task04)	Organ segmentation	1	394	nii.gz
		ISLES22	Lesion segmentation	1	400	nii.gz
		ATLAS v2.0	Lesion segmentation	1	1,271	nii.gz
		WMH	Lesion segmentation	1	170	nii.gz
		L2R-OASIS	Segmentation/registration	35	416	nii.gz

**Table 13.** Summary of eyes dataset within the IPIU large-scale medical imaging dataset

Dimensions	Modality	Dataset	Task	Targets	Data size	File format
2D	OCTA	OCTA-500	Organ segmentation	1	300	png
		ROSE	Organ segmentation	1	229	png, tif
	Fundus images	RFMiD 2.0	Classification	45	3,200	png
		JSIEC	Classification	39	1,000	jpg
		DRIVE	Organ segmentation	1	40	tif, gif
		PALM19	Organ segmentation	1	1,200	png
		REFUGE	Classification/segmentation	2	1,200	png
		Retina	Classification	4	601	png

**Table 14.** Summary of thorax dataset within the IPIU large-scale medical imaging dataset

Dimensions	Modality	Dataset	Task	Targets	Data size	File format
2D	Pathology	BreaKHis	Classification	2	7,909	png
		LungHist700	Classification	7	691	jpg
		WSSS4LUAD	Lesion segmentation	3	10,211	png
		ANHIR	Registration	-	481	png, jpg
	CT	COVID_CT_COVID-CT	Classification	2	746	png, jpg
		SARS-COV-2 Ct-Scan	Classification	2	2,482	png
		Chest CT-Scan images	Classification	4	1,000	png, jpg
		COVID-19-CT SCAN IMAGES	Classification	2	1,400	png, jpg, jpeg
	X-ray	COVID-19 CHEST X-RAY	Classification	3	3,886	png
		Chest X-ray PD Dataset	Classification	3	4,575	jpg, png
		COVIDGR	Classification	2	852	jpg
		MIAS	Classification	7	322	png
		CoronaHack	Classification/segmentation	7	5,910	png
		SZ-CXR	Classification/segmentation	1	566	png
		ChestX-Det10	Instance detection	10	3,543	png
	Ultrasound	BUSI	Classification/segmentation	3	780	png
		Breast Ultrasound Dataset B	Lesion segmentation	1	163	png
3D	CT	LUNA16	Detection/segmentation	1	888	mhd
		ATM22	Organ segmentation	1	500	nii.gz
		MSD Lung Tumours (Task06)	Lesion segmentation	1	96	nii.gz
		Parse 2022	Organ segmentation	1	200	nii.gz
		StructSeg 2019 Task3	Organ segmentation	6	50	nii.gz
		StructSeg 2019 Task4	Lesion segmentation	1	50	nii.gz
		SegTHOR	Organ segmentation	4	60	nii.gz
		FUMPE	Lesion segmentation	1	35	nii.gz
		LNDb	Lesion segmentation	1	294	nii.gz
		LNQ 2023	Lesion segmentation	1	413	nrrd
		PleThora	Organ segmentation	2	402	dicom, nii.gz
	MRI	ACDC	Organ segmentation	3	150	nii.gz
		MSD Cardiac (Task02)	Organ segmentation	1	30	nii.gz
		LAScarQS 2022	Organ segmentation	2	194	nii.gz
		MyoPS 2020	Organ segmentation	5	45	nii.gz
		MM-WHS	Organ segmentation	7	120	nii.gz
		CMRxMotion	Organ segmentation	3	360	nii.gz
	Ultrasound	MVSeg-3DTEE 2023	Organ segmentation	2	175	nii.gz
		TDSC-ABUS2023	Lesion segmentation	1	200	nrrd

**Table 15.** Summary of abdomen dataset within the IPIU large-scale medical imaging dataset

Dimensions	Modality	Dataset	Task	Targets	Data size	File format
2D	Pathology	Gleason 2019	Classification	6	311	jpg
		PANDA	Classification	5	331	jpg
		EBHI-Seg	Lesion segmentation	6	4,456	png
		CRAG	Lesion segmentation	1	213	png
		AGGC	Lesion segmentation	5	203	tif
	Endoscope	Colonoscopic	Lesion detection	2	76*2	mp4
		CP-CHILD	Classification	2	9,500	jpg
		Kvasir-SEG	Lesion segmentation	1	1,160	jpg
		CVC-ClinicDB	Lesion segmentation	1	612	tif
		EAD 2019	Organ segmentation	7	2,991	jpg, tif
		PSVFMs	Organ segmentation	1	483	png
		CholecSeg8k	Organ segmentation	13	8,080	png
		C3VD	Reconstruction	22	10,015	png, tiff, txt
3D	CT	FLARE 2021	Organ segmentation	4	511	nii.gz
		FLARE 2022	Organ segmentation	12	2,300	nii.gz
		FLARE 2023	Organ segmentation	14	4,500	nii.gz
		WORD	Organ segmentation	16	150	nii.gz
		AbdomenCT-1K	Organ segmentation	4	1,112	nii.gz
		3D-IRCADB	Organ segmentation	40	22	dicom, vtk
		LiTS	Lesion segmentation	2	201	nii.gz
		KiTS19	Lesion segmentation	2	300	nii.gz
		KiTS21	Lesion segmentation	3	400	nii.gz
		KiTS23	Lesion segmentation	3	599	nii.gz
		BTCV	Organ segmentation	13	50	nii.gz
		BTCV Cervix	Organ segmentation	4	50	nii.gz
		CHAOS	Organ segmentation	4	40	dicom
		MSD Liver (Task03)	Lesion segmentation	2	210	nii.gz
		MSD Prostate (Task05)	Organ segmentation	1	48	nii.gz
		MSD Pancreas Tumour (Task07)	Lesion segmentation	2	420	nii.gz
		MSD Spleen (Task09)	Organ segmentation	1	61	nii.gz
		MSD Colon Cancer (Task10)	Lesion segmentation	1	190	nii.gz
		SLIVER07	Organ segmentation	1	30	mhd
		QUBIQ2021-3D CT	Lesion segmentation	2	90	nii.gz
	CTA	KiPA22	Organ segmentation	4	130	nii.gz
	MRI	AMOS 2022	Organ segmentation	15	600	nii.gz
		ATLAS	Lesion segmentation	2	90	nii.gz
		SPPIN	Lesion segmentation	1	111	nii.gz
		CHAOS	Organ segmentation	4	40	dicom
		PROMISE12	Organ segmentation	1	50	mhd
		PI-CAI	Lesion segmentation	1	1,500	mha
		LLD-MMRI2023	Detection	7	394	nii.gz
		PanSegData	Organ segmentation	1	767	nii.gz

**Table 16.** Summary of lesion dataset within the IPIU large-scale medical imaging dataset. Given the distinct characteristics of lesion-related tasks in terms of target scale and diagnostic relevance compared to conventional anatomical structure analysis, we separately summarize them to more specifically highlight their significance in the development of medical foundation models.

Dimensions	Modality	Dataset	Task	Targets	Data size	File format
2D	Pathology	WSSS4LUAD	Lesion segmentation	3	10,211	png
		EBHI-Seg	Lesion segmentation	6	4,456	png
		CRAG	Lesion segmentation	1	213	png
		AGGC	Lesion segmentation	5	203	tif
	Ultrasound	TN-SCUI2020	Lesion segmentation	1	4,554	png
		DDTI	Lesion segmentation	1	637	png
		TG3K	Lesion segmentation	1	3,585	jpg
		TN3K	Lesion segmentation	1	3,493	jpg
		Breast Ultrasound Dataset B	Lesion segmentation	1	163	png
	Endoscope	Colonoscopic	Lesion detection	2	76*2	mp4
		Kvasir-SEG	Lesion segmentation	1	1,160	jpg
		CVC-ClinicDB	Lesion segmentation	1	612	tif
3D	PET-CT	AutoPET 1 (2022)	Lesion segmentation	1	1,164	nii.gz
		AutoPET 2 (2023)	Lesion segmentation	1	1,214	nii.gz
		AutoPET 3 (2024)	Lesion segmentation	1	1,614	nii.gz
		HECKTOR 2022	Lesion segmentation	2	882	nii.gz
	CT	INSTANCE 2022	Lesion segmentation	1	200	nii.gz
		SegRap2023	Organ/lesion segmentation	45/2	200	nii.gz
		MSD Lung Tumours (Task06)	Lesion segmentation	1	96	nii.gz
		StructSeg 2019 Task4	Lesion segmentation	1	50	nii.gz
		FUMPE	Lesion segmentation	1	35	nii.gz
		LNDb	Lesion segmentation	1	294	nii.gz
		LNQ 2023	Lesion segmentation	1	413	nrrd
		LiTS	Lesion segmentation	2	201	nii.gz
		KiTS19	Lesion segmentation	2	300	nii.gz
		KiTS21	Lesion segmentation	3	400	nii.gz
		KiTS23	Lesion segmentation	3	599	nii.gz
		MSD Liver (Task03)	Lesion segmentation	2	210	nii.gz
		MSD Pancreas Tumour (Task07)	Lesion segmentation	2	420	nii.gz
		MSD Colon Cancer (Task10)	Lesion segmentation	1	190	nii.gz
		QUBIQ2021-3D CT	Lesion segmentation	2	90	nii.gz
		MSD Hepatic Vessel (Task08)	Lesion segmentation	2	443	nii.gz
	MRI	BraTS21	Lesion segmentation	3	2,040	nii.gz
		BraTS2023-SSA	Lesion segmentation	3	105	nii.gz
		BraTS2023-MET	Lesion segmentation	3	328	nii.gz
		BraTS2023-MEN	Lesion segmentation	3	1,650	nii.gz
		BraTS2023-PED	Lesion segmentation	3	228	nii.gz
		BraTS-TCGA-LGG	Lesion segmentation	3	65	nii.gz
		BraTS-TCGA-GBM	Lesion segmentation	3	102	nii.gz
		MSD Brain (Task01)	Lesion segmentation	3	750	nii.gz
		ISLES22	Lesion segmentation	1	400	nii.gz
		ATLAS v2.0	Lesion segmentation	1	1,271	nii.gz
		WMH	Lesion segmentation	1	170	nii.gz
		ATLAS	Lesion segmentation	2	90	nii.gz
		SPPIN	Lesion segmentation	1	111	nii.gz
		PI-CAI	Lesion segmentation	1	1,500	mha
	Ultrasound	TDSC-ABUS2023	Lesion segmentation	1	200	nrrd

**Table 17.** Summary of head and neck dataset within the IPIU large-scale medical imaging dataset

Dimensions	Modality	Dataset	Task	Targets	Data size	File format
2D	Pathology	OSCC	Classification	2	1,224	jpg
	OCT	OCTMNIST	Classification	4	109,309	png
	Ultrasound	TN-SCUI2020	Lesion segmentation	1	4,554	png
		DDTI	Lesion segmentation	1	637	png
		TG3K	Lesion segmentation	1	3,585	jpg
		TN3K	Lesion segmentation	1	3,493	jpg
3D	CT	NasalSeg	Classification	6	130	nrrd
		SegRap2023	Organ/Lesion segmentation	45/2	200	nii.gz
		PDDCA	Organ segmentation	9	48	nrrd
	PET-CT	HECKTOR 2022	Lesion segmentation	2	882	nii.gz
	CT / MRI	HaN-Seg	Organ segmentation	30	42	nrrd
	IOS	ToothFairy	Organ segmentation	1	443	npy
		Teeth3DS	Teeth segmentation	32	1,800	obj, json

**Table 18.** Summary of vessel dataset within the IPIU large-scale medical imaging dataset

Dimensions	Modality	Dataset	Task	Targets	Data size	File format
3D	CTA	SEG.A.	Organ segmentation	1	56	nrrd
		ImageCAS	Organ segmentation	1	1,000	nii.gz
	MR	COSMOS2022	Organ segmentation	1	75	dicom
	CT	VESSEL12	Organ segmentation	1	20	tar.bz2
		MSD Hepatic Vessel (Task08)	Lesion segmentation	2	443	nii.gz

**Table 19.** Summary of skeleton dataset within the IPIU large-scale medical imaging dataset. The original publications or official sources of the datasets are provided in Tables S8 to S16.

Dimensions	Modality	Dataset	Task	Targets	Data size	File format
3D	CT	RibFrac 2020	Detection	2	1,224	jpg
		VerSe19	Organ segmentation	26	160	nii.gz
		VerSe20	Organ segmentation	26	319	nii.gz
		CTSpine1K	Organ segmentation	25	1,005	nii.gz
		CTPelvic1K	Organ segmentation	4	1,184	nii.gz
		MICCAI2024 PENGWIN	Organ segmentation	3	100	mha
	MRI	SPIDER	Organ segmentation	19	544	mha
		IVDM3Seg	Organ segmentation	1	16	nii.gz

To ensure data integrity and usability, the platform adheres to the following 3 fundamental criteria during the dataset inclusion process: (a) Completeness of annotation: Each dataset should contain clear and structured annotation information (e.g., classification labels and segmentation masks), enabling effective training and evaluation of models for various supervised learning tasks. (b) Usability of data format: Original medical images must be strictly aligned with their corresponding annotations and organized using a standardized storage structure and naming convention. (c) Task adaptability: Datasets should encompass diverse clinical scenarios, disease types, and imaging modalities, ensuring sufficient representativeness to support research requirements in terms of accuracy, generalizability, and reproducibility of algorithms.

### Medical VFMs

#### Visual expert models

##### Backbone algorithms for segmentation and detection of medical organs and lesions

This system establishes a large-scale framework for medical image segmentation, encompassing a comprehensive pipeline including image preprocessing, diversified backbone model selection, image post-processing, universal model inference, and performance verification. It integrates 27 algorithmic variants based on CNN [8], Transformer [132], Mamba [133], LSTM [134], and SAM [135] backbones, with model sizes ranging from 6 million to 1.4 billion parameters, offering medical researchers a rich set of model choices and benchmarking options. Furthermore, the framework supports multiple training and inference paradigms, including zero-shot training, supervised pretraining, and zero-shot inference, allowing flexible adaptation to a wide range of complex medical application scenarios.

##### Expert algorithm for risk prediction of medical lesions

This system is designed for the risk prediction of intracranial aneurysm rupture, incorporating a suite of classical machine learning algorithms, including decision trees, logistic regression, Lightgbm (LGBM), additive Bayesian models, naive Bayes, support vector machines (SVMs), and k-nearest neighbors (KNNs). At the same time, it also supports deep learning algorithms based on CNN and Transformer backbone architectures. This system aims to build a complete platform for simultaneous processing of multi-omics data classification and prediction tasks, integrating “lesion detection–lesion classification–risk prediction” into a unified framework, to improve the robustness and interpretability of the algorithm.

#### Visual universal models

##### Universal algorithms for segmentation and detection of medical organs and lesions

This system relies on the abovementioned backbone network framework platform and embeds a variety of universal segmentation model algorithms, aiming to use only one model to complete multiple medical vision tasks. This system divides them into the following 3 categories: (a) universal segmentation based on task prompts [136], (b) universal segmentation of vision-language (language prompts) [29], and (c) universal segmentation based on pretraining [60]; this system also supports unified preprocessing of multi-task data, general network training, and multi-task verification.

##### Large-scale universal pretraining algorithms for medical imaging

Medical image pretraining algorithms can provide substantial visual priors for downstream models trained from scratch, thereby enhancing the robustness and effectiveness of expert-designed methods. This system supports state-of-the-art supervised [60,63] and unsupervised pretraining [57] algorithms, enabling the model to learn intrinsic representations from vast amounts of medical imaging data under limited or no manual annotations. These learned priors offer valuable support for a wide range of downstream medical tasks with diverse requirements.

#### Visual generation models

To address the challenges of limited availability of multi-modal medical images and the high cost of manual annotation, this system introduces a medical image enhancement module based on generative FMs [36]. It aims to improve the training efficiency and generalization capability of downstream models through multi-modal image synthesis and low-quality image enhancement.

Specifically, to mitigate the scarcity of multi-modal medical data, the system integrates diffusion models for their powerful capability in modeling complex data distributions. High-quality synthetic images can be generated with only a small number of real samples. Structural preservation constraints and modality consistency mechanisms are integrated into the generation process to ensure that the synthesized images retain critical medical semantics, such as anatomical structures and tissue boundaries [35]. This effectively alleviates the limitations imposed by insufficient training data.

In addition, considering the heterogeneity of imaging equipment and the variation in clinical imaging conditions, the system supports image reconstruction and fine-grained detail enhancement based on diffusion models. By restoring high-resolution and structurally clear images from low-quality inputs, the system significantly improves the accuracy and robustness of downstream tasks such as segmentation and detection, thereby enhancing the applicability of models in real-world clinical environments.

Overall, this generative module not only enriches the diversity and stability of model training at the data level but also provides a practical solution to key challenges in medical imaging tasks, such as cross-modality modeling and low-resource learning. It demonstrates strong scalability and broad potential for real-world clinical applications.

### Medical language large models

Conventional medical retrieval-augmented generation (RAG) models [137] struggle to accurately capture the discriminative features of diseases with high semantic similarity due to their reliance on semantic similarity-based retrieval. To address this challenge, the IPIU medical FM platform proposes a novel integrated framework that combines the DeepSeek LLM, a structured MKG, and the RAG paradigm. This approach aims to explore the deep synergy between structured knowledge representations and language modeling. Technically, the framework leverages structured information from the MKG—such as disease characteristics, diagnostic pathways, and differential logic—and integrates it with the retrieval capabilities of the RAG architecture to assist the LLM in generating evidence-based diagnostic suggestions [138]. First, the diagnostic pathways encoded in the graph are used to constrain the LLM’s reasoning process. By aligning a patient’s clinical presentation with relevant graph nodes, the model incrementally constructs differential diagnostic hypotheses. Second, an active inquiry mechanism is designed based on the structured diagnostic logic of the knowledge graph. This mechanism utilizes the generative capacity of the LLM to automatically trigger context-aware follow-up questions (e.g., regarding disease progression or test result correlations) according to the current reasoning state [139]. In doing so, the framework establishes a dynamic “reasoning–inquiry–correction” decision loop, enhancing both the interactivity and interpretability of the diagnostic process.

### Medical VLMs

The VLM is designed to perform target-specific detection and fine-grained segmentation guided by natural language descriptions [59]. In contrast to traditional prompt-based approaches, natural language queries offer a more flexible and intuitive interface, enabling dynamic and interactive medical image analysis. However, conventional referring expression segmentation methods, when applied to medical images, typically rely on single-class supervision and fail to capture the complex relationships among different anatomical structures or pathological targets. This limitation often results in suboptimal performance in clinical applications [140].

**Fig. 11.**
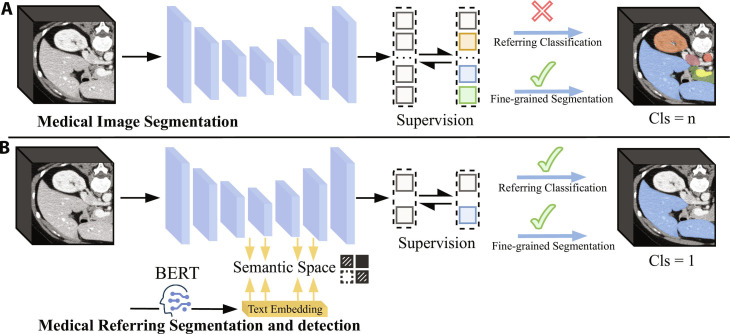
(A) Medical image segmentation. (B) Medical referring expression segmentation and detection, which localizes and segments specific target regions based on textual descriptions.

To address this issue, we propose a novel medical referring segmentation and detection framework, which is the first to extend referring expression techniques to medical image analysis. Our approach leverages language-driven supervision to identify and delineate multiple organs and lesions of interest based on descriptive queries, as illustrated in Fig. 11. By modeling inter-class dependencies and context-aware semantics, the system effectively bridges the gap between language expressions and medical visual patterns. Specifically, textual embeddings are extracted using a pretrained language encoder (BERT) and integrated with multi-scale visual representations through a cross-modal fusion mechanism, enabling accurate localization and segmentation of multiple organs and lesion targets. Moreover, this model is constructed by integrating visual and language model interfaces provided by the IPIU platform, and also supports independent user invocation. Extensive experiments on multi-organ segmentation and lesion detection tasks demonstrate the effectiveness and generalizability of our method, highlighting its potential for real-world clinical deployment in dynamic, language-guided medical workflows.

### Large-scale verification platform

The scoring system plays a critical role in the development and evaluation of FMs in the medical domain [32]. It not only provides quantitative metrics to assess model performance but also helps developers and researchers to identify strengths and weaknesses, thereby guiding further optimization and improvement. Given the complexity of medical applications, evaluation must consider not only accuracy but also efficiency, stability, and real-world clinical performance. The IPIU large-scale validation platform offers a standardized framework, enabling systematic comparison of different models under unified criteria to ensure their effectiveness in practical clinical settings.

Regarding the development environment, the IPIU large-scale verification platform is uniformly built on Python 3.10 and PyTorch 2.0.0, with computational resources provided by the IPIU High-Performance Computing Center. Model training and testing are conducted primarily on 4 servers, each equipped with 8 NVIDIA V100 GPUs (32 GPUs in total), ensuring computational efficiency and experimental stability. Subsequently, we present validation examples across classification, segmentation, and medical question answering tasks.

#### Classification tasks

For cerebral aneurysm rupture risk prediction, we conduct comprehensive evaluations of conventional machine learning and deep learning methods on a private dataset comprising 51 features, including pathological, morphological, and hemodynamic indicators [25]. All raw data are uniformly processed through standardization, categorical encoding, and missing value imputation. Machine learning models are optimized via grid search on the training set. Deep learning models are independently trained on the same training set for 100 epochs using the Adam optimizer with a learning rate of 0.001 and a batch size of 64. Cross-entropy loss is used as the objective function. We adopt 5-fold cross-validation and bootstrapping to compare the performance of all methods in terms of accuracy (ACC) and AUC [48], with a 95% confidence interval. As shown in Table 20, deep learning models generally achieve superior predictive performance on this task.

**Table 20.** Comparison of machine learning and deep learning methods on the task of predicting cerebral aneurysm rupture risk.

Method		Fivefold cross-validation		Bootstrap validation (95% CI)		Public
		ACC	AUC	ACC	AUC	
Machine learning	Logistic Regression [191]	0.58	0.7296	0.4400–0.7200	0.5684–0.8669	✓
	XGBoost [192]	0.50	0.8152	0.3600–0.6200	0.4650–0.7773	✓
	SVM [193]	0.50	0.7056	0.3600–0.6000	0.5454–0.8583	✓
	KNN [194]	0.60	0.7216	0.4600–0.7400	0.5771–0.8622	✓
	Lightgbm [195]	0.50	0.7178	0.3600–0.6000	0.5652–0.8542	✓
	Decision Tree [196]	0.52	0.596	0.3800–0.6000	0.4597–0.7350	✓
	Naive Bayes [197]	0.50	0.6632	0.3800–0.6000	0.5103–0.8132	✓
	GBDT [196]	0.66	0.5952	0.5200–0.8000	0.4316–0.7683	✓
Deep learning	DANETs [198]	0.56	0.7536	0.4200–0.7000	0.6038–0.8719	✓
	TabTransformer [199]	0.54	0.7296	0.4000–0.6800	0.5746–0.8727	✓
	MLP	0.64	0.776	0.5200–0.7600	0.6190–0.9018	**✗**
	TabNet [200]	0.50	0.8384	0.3600–0.6400	0.7198–0.9343	✓
	FT-Transformer [201]	0.62	0.7488	0.4800–0.7600	0.5942–0.8879	✓
	GANDALF [202]	0.50	0.816	0.3600–0.6400	✓	✓

#### Segmentation tasks

To address the lack of clear comparative benchmarks and partitioning in current medical image segmentation applications [8], our research team selected several commonly used public datasets from recent top-tier journals, conferences, and international research competitions, and conducted evaluations across 5 fundamental medical tasks: whole body, brain, lesion, vessel, and skeleton segmentation. For each task, 2 representative datasets are selected.

To ensure fair comparisons with state-of-the-art methods and support reproducibility, key experimental variables are carefully controlled and standardized evaluation protocols are adopted. Specifically, to minimize domain discrepancies across datasets, we apply the preprocessing strategy from nnU-Net [8], including normalization and resampling of the raw data. All models receive input volumes of size 96×96×96, with a batch size of 2, and are trained for 500 epochs. Regarding training protocols, we follow the original implementations of each baseline method, using the same optimizers, learning rate schedules, and loss functions. Additionally, 20% of each dataset is randomly selected as the testset, and all methods are uniformly evaluated using DSC and mIoU metrics [48]. This setup ensures consistency across different models and tasks.

As shown in Table 21, the system has integrated a wide range of advanced FM algorithms across the 5 core tasks and conducted comprehensive evaluations. Analyses have been performed from multiple dimensions, including training resources and performance outcomes, leading to the following conclusions:

1. The most influential factors in training strategies include batch size and patch size, which directly impact model performance, along with the number of training epochs, optimizer selection, and data preprocessing operations.
2. Advanced architectural designs such as Transformers, Mamba, vision LSTMs, and large-kernel convolutional operators have demonstrated strong performance; however, they often incur substantial computational overhead, limiting their scalability and deployment feasibility.
3. Lightweight architectures such as CNNs, Mamba-based models, and vision-oriented LSTMs demonstrate computationally efficient characteristics suitable for large-scale training while achieving state-of-the-art performance through principled network design.
4. Prompt-based methods like SAM rely heavily on sophisticated prompt engineering. In prompt-free scenarios, their performance is limited, and training from scratch introduces significant computational and annotation costs.
5. 3D CNNs remain the most effective and scalable foundational operators for volumetric medical image analysis, especially in large-scale training settings.
6. The design of visual perception modules is critical in determining segmentation performance, as different architectural choices can significantly impact both accuracy and generalization.

**Table 21.** Compare the performance of current state-of-the-art (SOTA) methods on 5 types of datasets: whole-body, vessel, brain, lesion, and skeleton under the same experimental settings

Method						Params (M)	Whole-body		Vessel		Brain		Lesion		Skeleton	
							DSC (%)	mIoU (%)	DSC (%)	mIoU (%)	DSC (%)	mIoU (%)	DSC (%)	mIoU (%)	DSC (%)	mIoU (%)
CNN	SegResNet [203]	18.8	81.59	71.95	75.88	66.25	84.42	73.78	77.96	68.74	72.18	62.14
	nnU-Net [8]	31.0	84.32	75.43	76.24	66.97	84.63	73.96	76.85	67.77	74.32	63.99
	3D UX-Net [204]	53.0	81.52	71.84	74.98	64.88	83.81	73.25	75.58	66.65	72.00	61.98
	STU-Net*_s_* [60]	14.6	83.29	73.67	74.26	64.13	83.51	72.98	73.62	64.92	71.99	61.79
	STU-Net*_b_* [60]	58.3	84.41	75.14	76.00	56.76	84.76	74.08	83.14	73.31	86.91	76.48
	STU-Net*_l_* [60]	440.3	85.93	75.96	78.71	68.95	84.69	74.02	81.23	71.66	82.73	72.80
	STU-Net*_h_* [60]	1,457.3	88.85	80.29	79.89	70.06	84.66	73.99	82.45	72.71	84.67	74.51
	MedNeXt*_S_* [205]	5.9	83.34	73.88	77.72	67.59	84.64	73.97	80.29	70.80	80.67	70.98
	MedNeXt*_B_* [205]	11.0	84.97	75.67	76.34	66.87	84.40	73.77	79.61	70.26	76.08	65.50
	MedNeXt*_M_* [205]	18.3	86.22	78.15	76.16	66.57	84.38	73.74	80.68	71.14	80.56	70.89
	MedNeXt*_L_* [205]	63.0	87.65	79.53	76.12	66.51	84.26	73.64	81.74	72.08	76.94	66.25
Transformer	UNETR [206]	92.8	74.99	64.57	73.71	63.87	83.81	73.25	66.25	58.42	63.56	51.25
	Swin-UNETR [132]	62.2	80.00	71.24	74.27	64.57	84.15	73.54	71.45	63.01	65.28	52.69
	Swin-UNETR* [63]	62.2	80.94	71.85	74.51	63.85	84.53	73.88	75.54	66.47	63.59	51.28
	UNesT*_s_* [207]	22.4	81.66	72.15	75.44	65.83	84.77	74.08	76.04	66.91	62.41	50.48
	UNesT*_b_* [207]	87.2	80.34	71.52	70.77	61.12	84.93	74.22	75.19	66.16	73.35	63.15
	UNesT*_l_* [207]	279.5	81.42	72.18	74.57	65.99	84.96	74.25	74.34	65.42	75.43	64.96
	DHT- Net [208]	58.09	83.53	73.68	76.89	67.21	84.96	74.26	77.56	68.25	75.12	64.67
	EPT- Net [209]	60.69	84.62	75.79	76.27	66.53	84.46	73.81	77.29	68.05	74.96	64.54
Mamba	LightM-UNet [210]	6.2	80.40	71.56	77.27	67.91	83.42	72.91	79.92	70.34	69.81	58.14
	U-Mamba*_Enc_* [211]	59.7	84.11	75.23	74.33	64.58	84.54	73.88	74.24	65.33	67.13	56.84
	U-Mamba*_Bot_* [211]	41.9	84.47	75.65	75.55	65.83	84.51	73.86	74.08	65.19	74.57	64.20
	SegMamba [133]	67.4	82.53	72.68	75.25	65.71	84.45	73.89	73.38	64.57	72.24	62.19
LSTM	xLSTM-UNet*_E_* [134]	41.91	84.25	75.55	77.48	67.85	84.52	73.87	78.85	69.39	75.40	64.92
	xLSTM-UNet*_B_* [134]	43.53	84.50	75.69	77.73	68.13	84.57	73.91	77.54	68.23	74.59	64.22
SAM	SAM-Med 3D [135]	103.65	82.36	71.46	71.24	60.56	80.67	70.13	64.67	52.16	72.78	60.49
	SAM-Med 3D*_b_* [135]	103.65	82.90	71.67	72.23	61.67	82.31	72.42	68.43	55.76	73.48	61.78

#### Medical question answering tasks

To systematically evaluate the performance of medical LLMs, we selected 4 commonly used medical question answering benchmarks: MedQA, MedMCQA, PubMedQA, and MedXpert. Multiple 7-8B scale models were adopted as baselines for comparison. All models were fine-tuned for 3 epochs using a learning rate of 5 × 10^−6^ and a batch size of 128, and evaluated on the respective test sets. As shown in Table 22, The preliminary conclusions are summarized as follows:

1. Different LLMs exhibit varying levels of performance across different medical question answering (QA) benchmarks.
2. The MedReason model, which incorporates external medical knowledge, demonstrates the best overall performance.

**Table 22.** Performance comparison of representative medical LLMs across multiple benchmarks

Model name	Params	Context length	MedXpert	MedQA	MedMCQA	PubmedQA	Avg	Public
Llama3.1-Instruct [212]	8B	128k	12.53	51.45	49.08	65.91	44.74	✓
Qwen2.5-Instruct [213]	7B	128k	11.04	49.96	48.73	63.72	43.36	✓
HuatuoGPT-o1 [214]	8B	128k	15.16	61.53	51.01	66.70	48.60	✓
DeepSeek-Distill [215]	8B	128k	11.83	48.56	42.95	64.77	42.03	✓
MedReason [216]	8B	128k	16.65	62.93	53.20	69.59	50.59	✓

## Medical Applications of FMs

**Fig. 12.**
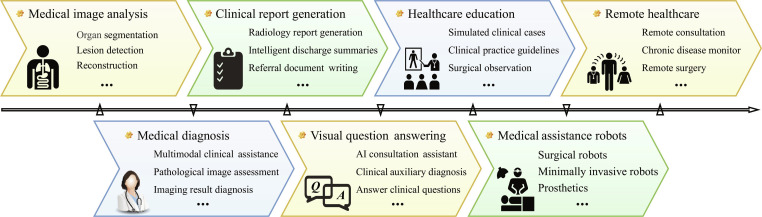
Typical applications of medical foundation models.

With the rapid development of deep learning, FMs based on large-scale pretraining are reshaping the research paradigm of modern medical AI [15,16]. Studies have shown that by integrating multi-modal data such as medical text, imaging, and pathology slides, and incorporating domain-adaptive fine-tuning techniques, FMs not only demonstrate strong cross-modal understanding and efficient knowledge transfer capabilities but also enable the development of cognitive reasoning systems tailored to specific clinical scenarios. These developments are accelerating the transformation of traditional experience-driven medical practice toward a data-intelligent paradigm [17]. The following sections provide a systematic overview of their representative applications across key tasks, including medical image analysis, clinical diagnosis, report generation, VQA, medical education, and remote healthcare, as illustrated in Fig. 12.

### Medical image analysis

With the rapid development of FMs, medical image analysis is accelerating toward an intelligent stage driven by multi-modal large models. These models not only encompass a wide range of medical image interpretation tasks such as classification, segmentation, lesion detection, and generation but also demonstrate advanced semantic understanding, cross-modal question answering, and vision-language generation capabilities, thereby providing comprehensive support for clinical decision support [17].

For example, BME-X [31] proposed an FM for structural MRI quality enhancement, which can simultaneously achieve motion correction, resolution improvement, denoising, and cross-scanner coordination. It first predicts tissue labels via a classification network and then generates high-quality images using a tissue-aware enhancement module, achieving strong performance in segmentation, registration, and diagnosis tasks. Specifically, BME-X effectively addresses the issue of inconsistent image contrast caused by differences in magnetic field strength and pulse sequences across different MRI scanners (such as Siemens, GE, and Philips). Through a generative model, this approach maps MRI images from various scanners to a unified feature space, enabling the standardization of image appearance and histogram distribution without the need for a reference site. This technique significantly reduces scanner-related variability, improving the consistency and reliability of images in multi-center studies and clinical diagnoses, thereby promoting the standardization of image quality across different devices. This achievement holds considerable practical significance in clinical applications, particularly in multi-center studies across institutions and devices, as it greatly enhances the comparability of MRI image data and the accuracy of diagnoses.

Med-SA [141] improves segmentation performance without extensive large-scale fine-tuning, through lightweight structural adaptation and the introduction of medical knowledge.

ProMISe [142], built upon the pretrained 2D SAM Transformer, integrates lightweight adapters to capture depth-related 3D spatial context without updating the original pretrained weights. Furthermore, it introduces a complementary encoder and a boundary-aware loss, which jointly mitigate the under-segmentation of the colon and the over-segmentation of pancreatic tumors, thereby achieving precise segmentation.

MoME [143] introduces a modality-general FM for brain MRI lesion segmentation. It constructs a hybrid architecture composed of multiple MRI modalities, with each modality (e.g., T1 and T2) individually optimized, and coordinates them through a unified FM. By leveraging the complementary features of different modalities, MoME effectively integrates multi-modal information, thereby achieving significantly improved accuracy and robustness in brain lesion segmentation tasks.

Swin-UMamba [54] effectively reduces the distribution differences between natural images and medical images by embedding a self-supervised module within the Mamba architecture, achieving excellent results on abdominal MRI, endoscopy, and microscopy datasets.

Moreover, the lightweight biomedical VLFM BiomedGPT [76], built on a Transformer architecture, follows the unified modeling principles of being modality-agnostic, task-agnostic, and comprehensive in coverage. By discretizing data into patches or tokens, it achieves unified input–output processing and supports a wide range of medical image interpretation tasks, including classification, retrieval, localization, Med-VQA, and report generation.

MINIM [34] can synthesize medical images of various organs and modalities based on clinical instructions, addressing the scarcity of high-quality medical imaging data. It significantly enhances performance across multiple clinical and research tasks, including disease diagnosis, report generation, and disease prediction, demonstrating its potential as a generalist medical AI.

In the epidermal growth factor receptor (EGFR) mutation detection task, MINIM uses a Swin Transformer to classify real chest CT images into 3 EGFR mutation types: wild-type, sensitive, and resistant. To enhance the training dataset, synthetic CT images are combined with real data. Experimental results show that as the ratio of synthetic to real data increases from 0:1 to 1:1 and 5:1, the classification accuracy improves from 81.5% to 91.2%, ultimately reaching 95.4% when the ratio is 5:1. Furthermore, in the binary classification task, the inclusion of synthetic data increased the area under the ROC curve (AUROC) from 74.2% to 96.5%, significantly enhancing the model’s performance in EGFR mutation detection. To validate the clinical effectiveness of the model, a retrospective clinical study was conducted, analyzing data from 2 independent patient cohorts. The study first established the baseline survival curves for patients receiving standard chemotherapy and then used the model to predict whether EGFR mutation-positive patients would benefit from tyrosine kinase inhibitor (TKI) targeted therapy. The results showed that patients with EGFR-sensitive mutations had a 5-year overall survival rate 29.5% higher than those with drug-resistant mutations, and their median survival time was significantly longer. These findings further validated the model’s effectiveness and generalizability in multi-center research. This study provides crucial technical support for the clinical application of EGFR mutation detection in lung cancer.

In the HER2 status detection task, MINIM first established a baseline classification model based on a real dataset to differentiate between no tumor, HER2-positive tumor, and HER2-negative tumor. By gradually incorporating synthetic images into the training dataset, when the ratio of synthetic to real images reached 10:1, the classification accuracy increased to 94.0%. This approach not only addresses the issue of scarce real data but also significantly improves the classification accuracy of the model, particularly in identifying HER2-positive patients, thereby providing more accurate diagnostic support for HER2-targeted therapy.

Overall, the above research achievements demonstrate significant progress in medical image interpretation technologies, particularly in multi-task processing and cross-modal analysis. They provide innovative solutions to challenges such as data scarcity, laying a solid theoretical and practical foundation for building efficient, intelligent, and personalized medical image analysis.

### Medical diagnosis

In medical diagnosis, FMs not only enable in-depth interpretation of medical images but also integrate multi-modal information such as medical texts and structured data, combining patient-reported symptoms with examination results to provide precise support for clinical decision-making [27]. Among these, medical image interpretation plays a central role, with related models being widely applied to tasks such as organ segmentation [97] and tumor and lesion detection [30], effectively improving the efficiency and accuracy of diagnosis.

Building on this foundation, several representative FMs have demonstrated remarkable performance. For example, CHIEF [144] combines image-level unsupervised learning and whole-slide weakly supervised pretraining to analyze pathology images from 44 TB of data across multiple countries. It demonstrates outstanding performance in cancer cell detection, tumor origin identification, molecular profile characterization, and prognostic prediction tasks, achieving efficient and highly generalizable cancer assessment.

In the genomic profiling prediction task, CHIEF successfully completed the prediction of multiple key genes through histopathology image analysis. In the prediction of prevalent genetic mutations, CHIEF analyzed 13,432 WSIs from 30 cancer types and 53 genes, accurately identifying several critical mutations. In the identification of mutations related to targeted therapies, the CHIEF system successfully predicted the mutation status of 18 genes associated with Food and Drug Administration (FDA)-approved targeted therapies, demonstrating exceptional performance, particularly in breast cancer (ESR1), lung adenocarcinoma (EGFR), and CRC (BRAF). Additionally, in the prediction of isocitrate dehydrogenase (IDH) status for gliomas, CHIEF stratified the research cohort by histological grading and predicted IDH status for each layer, revealing that necrotic areas received higher attention when identifying IDH wild-type. In the prediction of microsatellite instability (MSI) status for assessing the benefit of immune checkpoint inhibition in CRC patients, CHIEF successfully predicted the MSI status using histopathological images, focusing on areas of solid tumors, lumen necrosis, and tumor-infiltrating lymphocytes. This genomic profiling information provides important insights into treatment response, and CHIEF’s outstanding performance highlights its broad clinical potential.

In the prognostic prediction task, CHIEF focused on 7 cancer types [COADREAD, LUSC, BRCA, GBM, UCEC, LUAD, and renal cell carcinoma (RCC)] and successfully established a stage-based survival prediction model, distinguishing long-term survivors from short-term survivors. CHIEF achieved an average C-index of 0.74 across all cancer types, indicating superior prognostic performance. Further multivariate analysis showed that the risk score derived from CHIEF was an important prognostic factor, independent of known survival indicators, strengthening its significance in prognostic prediction. Univariate analysis showed significant correlations between the risk score and survival outcomes across all cancer types, further validating its clinical utility.

Moreover, CXRBase [145], by performing self-supervised pretraining on 1.04 million unlabeled CXRs and fine-tuning with a small amount of annotated data, learns generalizable representations that enable efficient adaptation to diverse clinical tasks, thereby enhancing disease detection performance and reducing the burden of expert annotations. HeartBEiT [146], based on a ViT combined with MIM, was pretrained on 8.5 million ECGs and exhibited superior performance over conventional CNNs, particularly under low-sample conditions, significantly improving accuracy and interpretability in the diagnosis of hypertrophic cardiomyopathy, reduced ejection fraction, and myocardial infarction.

Furthermore, the introduction of multi-modal FMs has further expanded the capabilities of medical image interpretation. MedKLIP [87] leverages structured medical triplet extraction and cross-modal alignment strategies to significantly enhance diagnostic performance on x-ray images. HiA [147] employs high-resolution visual encoding, instruction-aware feature extraction, and an information injection module to enable existing Chinese medical multi-modal LLMs to efficiently process multiple high-resolution images and perform comparative analysis in a plug-and-play manner without modifying the model parameters. Its effectiveness and training efficiency have been validated on the Chili–Joint dataset. RET-CLIP [148], built on a CLIP-style architecture and trained on data from 193,865 patients, extracts generalizable features from color fundus photographs using a triple-level optimization strategy. The model shows strong performance and versatility across 4 critical ophthalmic diagnostic tasks, including diabetic retinopathy, glaucoma, multi-disease diagnosis, and multi-label classification.

Overall, medical image interpretation helps clinicians identify critical information and provides more interpretable diagnostic support when integrated with clinical context. Building on this, the continuous evolution of FMs is gradually expanding their capabilities from single-task applications to comprehensive, cross-modal, and multi-task diagnostic support.

### Medical report generation

Various FMs have shown great potential in MRG, with current research focusing on text generation methods based on LLMs and multi-modal image-to-text generation approaches [45]. LLM-based methods integrate heterogeneous data sources, such as EHRs, clinical guidelines, and medical literature, and have made significant progress in structured report generation. Typical applications like automated discharge summaries and automated referral document writing BiomedGPT [76] demonstrate improved efficiency and standardization through precise interpretation of complex medical semantics.

Multi-modal image-to-text approaches focuses on generating structured text from medical images, with image interpretation as the core step to achieve cross-modal semantic alignment. As a representative model for radiology report generation, Flamingo-CXR [149] models radiology reports in an autoregressive manner conditioned on input images, and incorporates regularization together with task-adaptive fine-tuning, thereby enabling efficient integration and generation of medical image–text information, achieving report quality comparable to that of human experts in clinical evaluations.

In the collaboration between clinicians and Flamingo-CXR, Flamingo-CXR first generates a draft report, which is then refined by radiologists as needed, such as replacing sentences or adding additional content. This process allows radiologists and AI to co-author the report, making it “more concise” and “covey’s the clinical findings better”. The evaluation results show that in most cases, radiologists prefer or consider the clinician–AI edited report to be superior or equivalent to the original report. Specifically, in the MIMIC-CXR dataset, 53.6% of cases had at least half of the radiologists preferring or considering the clinician–AI report equivalent to the original, compared to 44.4% for reports generated solely by Flamingo-CXR. Similar results were confirmed in the IND1 dataset. This process demonstrates how foundational models, through collaboration with clinicians, can enhance the quality and practicality of medical imaging reports.

Moreover, PromptMRG [150] transforms diagnostic results into generation prompts through a disease classification branch, enhances cross-modal representations by incorporating similar reports retrieved from databases and pretrained CLIP knowledge, and applies adaptive logit adjustment to address disease imbalance, thereby significantly improving the accuracy and reliability of MRG. MEPNet [151] enhances the accuracy and completeness of brain CT report generation through a synergistic mechanism of medical entity embeddings and dynamic prompts. It employs knowledge-driven joint attention to extract entity visual embeddings, integrates a learning state scorer to evaluate and balance the learning difficulty of each entity, and injects these signals as multi-modal prompts into an LLM, thereby enabling more comprehensive, clinically accurate, and textually coherent report generation. CHATCAD+ [152] combines medical image interpretation with language interaction, which enhances the reliability of diagnosis and interaction through contextual learning and knowledge retrieval. Liu et al. [153] proposed a multi-granularity report generation framework that employs sentence-level image–sentence contrastive learning and a dual-decoder strategy to effectively learn abnormal features and generate topic-guided, fine-grained radiology reports without requiring additional annotations. COMG [154] introduces a multi-image report generation model guided by complex organ masks, which fuses organ-specific masks with disease priors and incorporates a cosine similarity loss to enhance cross-modal consistency. Collectively, these methods tightly couple image and text information, enabling models to extract key clinical features from medical images and generate more precise, comprehensive, and interpretable reports.

Overall, incorporating medical image interpretation into report generation systems enables models to integrate patient history and textual information to provide interpretable diagnostic support. The deep fusion of textual knowledge and imaging information, particularly the joint modeling of images and clinical data, is emerging as a key breakthrough in MRG, driving clinical decision support systems toward greater intelligence and reliability.

### Visual question answering

Med-VQA aims to accurately answer clinical questions by combining medical images and text information, demonstrating significant potential in supporting diagnosis and treatment [45]. A typical Med-VQA system consists of an image encoder, a question encoder, a multi-modal feature fusion module, and an answer prediction module. In recent years, Med-VQA systems have made notable progress through contrastive learning, meta-learning, and multi-task learning, which have effectively advanced multi-modal representation learning and improved model generalization capabilities.

On this basis, several representative models have been introduced to further advance the field. UnICLAM [155] uses adversarial masked contrastive learning to align image and text features, thereby improving the efficiency of multi-modal information fusion.

DFMD-VQA [156] proposes a deep fuzzy multi-teacher distillation (DFMD) network, which, for the first time, introduces fuzzy logic theory into Transformer-based multi-modal encoding. By integrating multi-teacher distillation with a robust encoder (FuzBERT), it effectively models the uncertainty and noise in cross-modal vision-language representations, thereby achieving significant performance improvements over existing methods on the VQA-RAD and SLAKE datasets. Med-Flamingo [157] significantly improves the few-shot generative Med-VQA performance based on vision-language joint pretraining. XrayGPT [158] combines the medical visual encoder with a fine-tuned language model to provide high-quality open-ended question answering for x-ray images. LLaVA-Med [159] demonstrates powerful multi-modal question answering capabilities based on large-scale image and text data and course learning training. Collectively, the above methods have substantially advanced the intelligent of Med-VQA systems. Future research will further combine MKGs, cross-modal pretraining frameworks, and interpretability mechanisms to further improve the practicality and reliability of Med-VQA systems in complex clinical scenarios.

Notably, these systems place medical image interpretation at their core, extracting key clinical features from images and integrating visual representations with reasoning capabilities, thereby enhancing the accuracy and reliability of the answers.

### Healthcare education

Medical FMs have demonstrated significant potential in medical education and are gradually becoming a key technology to improve the efficiency of medical knowledge acquisition and intelligent education systems. Leveraging their strong capabilities in language understanding and generation, FMs can assist medical students in clinical question answer, simulated case analysis, and exam preparation, thereby improving their autonomous learning and clinical reasoning abilities. For example, HuatuoGPT-II [160] unifies pretraining and fine-tuning under an instruction format training framework, effectively alleviating catastrophic forgetting and achieving outstanding performance on multiple traditional Chinese medicine benchmarks and pharmacist licensing exams.

In supporting personalized teaching and equitable access to knowledge, medical FMs are becoming the cornerstone of the intelligent medical education ecosystem. FMs are capable of efficiently retrieving and integrating academic literature, clinical guidelines, and real cases, thereby enabling rapid access to authoritative knowledge and promoting health education [149]. Furthermore, systems equipped with multi-modal understanding and generation capabilities can automatically produce medical image explanations and health education videos, enhancing the visualization of medical knowledge. Typical applications include virtual patient systems based on LLMs, which generate dynamic cases to train students’ clinical reasoning skills, and 5G ultrasound platforms that enable community physicians to remotely observe expert procedures, thereby facilitating the dissemination of practical skills.

To further advance deep cross-modal understanding and clinical reasoning, M^3^-Med [161] introduces the first benchmark specifically designed for medical education scenarios, featuring expert-annotated question–video pairs and the innovative introduction of multi-hop reasoning tasks. It further defines 2 subtasks: temporal answer grounding within a single video (TAGSV) and temporal answer grounding across a video corpus (TAGVC). The multi-hop reasoning task requires the model to first locate a key entity in the text, then identify the corresponding visual evidence in the video, and finally integrate information across modalities to derive the answer. This design spans the entire reasoning chain from entity localization to cross-modal evidence synthesis, not only overcoming the limitations of existing benchmarks that are confined to English and shallow retrieval but also exposing the deficiencies of current models in deep cross-modal understanding, thereby providing strong support for cultivating clinical reasoning skills in medical students and advancing intelligent medical education.

In addition, medical FMs demonstrate unique advantages in the construction of educational resources. Leveraging medical image interpretation, FMs can extract key clinical features from images and transform them into teaching materials, enabling students to grasp diagnostic logic and practical skills in multi-modal learning contexts. PLIP [162], trained on the pathology image–text dataset OpenPath, demonstrates outstanding performance in zero-shot classification and image–text retrieval tasks, providing an efficient and open intelligent support tool for medical education. Meanwhile, the EndoAssistant dataset [163], which encompasses large-scale and diverse endoscopic videos, images, subtitles, and image–text question–answer pairs, lays a foundation for training general VLFMs. It is suitable for a wide range of surgical endoscopy understanding tasks and offers strong support for medical education and intelligent surgical training.

### Medical assistance robots

FMs are accelerating the intelligent development of medical assistance robots by enhancing their capabilities in perception, reasoning, and cross-modal processing, thereby providing efficient support for clinical applications. High-quality datasets enable FMs to learn complex surgical scenarios and instrument manipulation features, and thus play a critical role in surgical planning, surgical navigation, and real-time monitoring. Leveraging these datasets, FMs empower robots assistance surgeries, significantly improving the accuracy and safety of surgical decision-making and driving the transformation of traditional medical practice toward intelligent, data-driven paradigms.

With the support of high-quality datasets, surgical assistance robots can achieve robust training and verification in diverse clinical environments. The 3D rendering dataset SARAMIS [164] enhances visual simulation capabilities for minimally invasive surgical tasks based on anatomical structures. Zeng et al. [165] proposed a synthetic surgical dataset generation method based on 3D Gaussian splatting, effectively alleviating the shortage of training data and demonstrating improvements in instrument detection performance for robot-assisted surgery.

Building on high-quality datasets, FMs demonstrate potential in surgical planning and surgical navigation. ZEAL [166] achieves objective surgical skill assessment through zero-shot instrument segmentation and temporal modeling, and its superiority has been validated on public datasets JIGSAWS. This approach not only provides new avenues for surgical training and improved patient prognosis but also lays the groundwork for integrating surgical navigation systems to enhance surgical process monitoring and decision support. Surgical-DINO [167] introduces low-rank adaptation (LoRA) into DINOv2, effectively transferring the FM to the task of surgical depth estimation. It demonstrates significant superiority over existing methods on the SCARED and Hamlyn endoscopic datasets, validating its potential in 3D reconstruction, surgical navigation, and augmented reality visualization, while also revealing the limitations of zero-shot prediction and naive fine-tuning in surgical applications. Furthermore, Surg-FTDA [168] leverages few-shot modality alignment and text-driven adaptation to enable laparoscopic surgical workflow analysis without the need for large-scale paired data. Specifically, it utilizes the CholecT50 dataset for triplet recognition, the Cholec80 dataset for phase recognition, and the SVL-Caption dataset for image captioning. This approach achieves strong performance across both generative and discriminative tasks, significantly reducing reliance on expert annotations while enhancing generalization capability.

Building on this foundation, integrating medical FMs into surgical robots provides a new technological paradigm for achieving intelligent, interpretable, and safely controllable autonomous surgical operations. For example, RT-RAS [169] enhances robotic autonomy and decision-making by constructing a multi-modal, multi-task vision–language–action model, thereby addressing challenges such as limited data, soft tissue modeling complexity, and safety constraints. The model is trained on real surgical demonstration data and optimized using postoperative outcomes and expert evaluations as feedback, enabling it to learn directly from clinical results and progressively surpass the quality ceiling of the original demonstrations. Its core innovation lies in not only generating surgical actions but also estimating the confidence of each decision through conservative Q-learning (CQL) and conformal prediction, allowing the system to proactively hand control back to the surgeon when uncertainty is high (“knows when they do not know and ask for help when needed”). Using laparoscopic cholecystectomy as an example, RT-RAS demonstrates how the model can identify anomalies, trigger low-confidence alerts, recommend human intervention, and record such events for iterative improvement—forming a closed-loop framework of explanation, intervention, feedback, and evolution. This work provides a viable pathway and reference paradigm for the safe deployment and continuous advancement of medical FMs in intelligent surgical robotics.

These advancements rely on the ability of FMs’ modeling and parameter optimization of multi-modal medical data, as well as the application of medical image interpretation in key feature recognition and scene understanding, thereby significantly enhancing medical robot’s understanding of complex physiological processes and autonomous decision-making capabilities [59]. With continued progress in cross-modal alignment and reasoning mechanisms, medical robots are expected to achieve breakthroughs in generalization capabilities, safety, and operational precision, contributing to the broader adoption of precision medicine.

### Remote healthcare

In areas with limited medical resources, medical FMs promote the advancement of remote healthcare toward intelligence and multi-scenario with cross-modal transfer learning and multi-modal data processing capabilities [15]. Specifically, FMs are capable of integrating large-scale clinical data and multi-modal information, thereby enhancing the intelligence and operational efficiency of remote healthcare systems. For example, ClinicalGPT [170] significantly improves the performance of tasks such as medical question answering and diagnostic analysis by integrating real clinical data and multi-task evaluation.

In medical imaging, FM-driven interpretation technologies can accurately analyze imaging data from modalities such as x-rays and MRI [44], and have been widely applied to assist in the screening of various diseases, including retinal disorders and skin cancer, thereby providing critical imaging support for remote healthcare. In the domain of diagnostic reasoning, RAG techniques enhance clinical inference by combining LLMs with medical knowledge bases.

For example, the RULE framework [171] mitigates factual bias in Med-LVLMs by calibrating the amount of retrieved context to control factual risk and employing a preference fine-tuning strategy to balance the model’s reliance on inherent knowledge and retrieved contextual information, thereby improving factual accuracy in Med-VQA and report generation tasks. Similarly, MMed-RAG [138] enhances the accuracy and cross-domain generalizability of medical VLFMs through a domain-aware retrieval mechanism, adaptive context selection, and a provable RAG-based fine-tuning strategy, leading to significant improvements in consistency for medical question answering and report generation.

At the level of patient management, FMs enable personalized interventions for chronic disease monitoring and postoperative rehabilitation by analyzing continuous physiological signals from wearable devices [120]. At the same time, the intelligent consultation model that integrates MKGs and dialogue systems has demonstrated its practical value in initial screening and risk assessment in scenarios such as emergency triage.

Furthermore, by leveraging the capabilities of FMs in cross-modal representation and reasoning, together with the role of medical image interpretation in key feature extraction and clinical semantic understanding, remote healthcare systems can facilitate disease screening, diagnosis, and follow-up management for primary care physicians through image transmission and real-time interpretation while integrating patients’ EHRs to enable personalized interventions [115]. This not only highlights the significant potential of FMs in advancing remote healthcare but also provides critical support for mitigating disparities in healthcare resource distribution and enhancing the efficiency and accessibility of remote healthcare services.

Overall, with the continuous advancement of FMs and the rapid development of medical image interpretation technologies, remote healthcare is expected to achieve more efficient cross-regional collaboration and resource allocation, thereby facilitating the widespread dissemination of high-quality healthcare services and promoting equity and sustainability in healthcare systems.

## Challenges and Opportunities

**Fig. 13.**
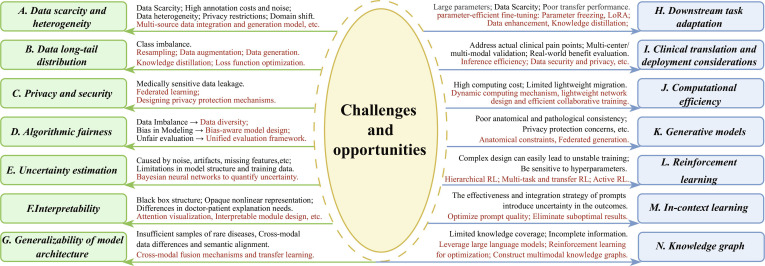
Overview of current challenges and opportunities for medical foundation models.

In the preceding sections, we systematically outlined the development of medical FMs in terms of datasets, evaluation metrics, model architectures, and representative applications, highlighting their theoretical foundations and potential in medical image interpretation. This section will focus on key challenges and explores future research directions and application prospects, as illustrated in Fig. 13, aiming to provide reference and inspiration for future work.

### Data scarcity and heterogeneity

Despite their impressive generalization in natural image, FMs face significant challenges in medical imaging due to data scarcity, multi-modal heterogeneity, imaging noise, and annotation errors [10]. On one hand, medical images heavily rely on real patient cases and are constrained by ethical and privacy regulations, resulting in high acquisition costs and limited dataset sizes, especially for rare diseases. Additionally, annotations require expert clinicians, making the process time-consuming, costly, and prone to subjective variability, leading to annotation noise.

On the other hand, medical images exhibit substantial modality heterogeneity, with significant semantic differences for the same anatomical structures across modalities, and strong coupling between artifacts and lesion feature. Semantic misalignment between images and texts also exists. These 3 issues further exacerbates the difficulty of modeling the FM under multi-modal and weakly supervised conditions. Moreover, medical image interpretation tasks demand high-resolution and fine-grained feature modeling [20], as small lesions are often highly localized, requiring models to possess both global context awareness and precise local localization capabilities. In practical applications, domain shifts caused by variations in imaging devices, institutions, and annotation standards significantly limit model generalization and clinical deployment feasibility.

To address the above challenges, researchers have proposed multiple strategies, including using generative models for data augmentation and completion to enhance sample diversity, quantity, and quality [34], employing cross-modal alignment mechanisms to alleviate heterogeneous information fusion issues and improve semantic consistency, and adopting unsupervised or self-supervised pretraining to enhance model representation under limited annotation [14]. Looking forward, with the continued development of technologies such as multi-source data integration, self-supervised pretraining, and generative technology, it is expected to effectively mitigate data scarcity and heterogeneity in medical imaging, driving the development of generalizable and transferable medical FMs for clinical applications.

### Data long-tail distribution

In medical imaging datasets, samples from different disease or lesion categories often exhibit an obvious long-tail distribution: A few high-frequency categories (“head categories”) comprise the majority of samples, while low-frequency categories (“tail categories”) contain only a small number of samples but frequently possess substantial clinical significance. The degree of imbalance can be quantified by the ratio of the largest to the smallest class [172]. Such long-tail distribution presents significant challenges for FMs. During training, models tend to be biased toward high-frequency classes, resulting in insufficient recognition of low-frequency classes and reduced reliability and generalization for rare cases or small lesions. Moreover, as the degree of class imbalance increases, model performance degrades more obviously, making the long-tail problem a major challenge for FMs in medical image interpretation.

To address the long-tail problem in medical imaging, current research primarily focuses on optimizations at 3 levels: data, model, and training strategies. At the data level, approaches include resampling to balance class distribution, data augmentation to expand the training dataset, and using generative models to synthesize semantically rich tail class samples, thereby enhancing their representativeness and diversity [34]. At the model level, researchers employ strategies such as decoupled learning and knowledge distillation (KD). These approaches are typically combined with feature enrichment, perturbation strategies, logit adjustment, and loss function optimization to improve the discriminability and robustness of tail-class samples. At the training and inference level, by combining training strategies such as optimizers and regularization, as well as post-processing methods, the models can better adapt to long-tail distributions during both training and inference, thereby improving prediction performance for minority classes.

For example, RadDiag [173] introduces a knowledge-enhanced vision-language contrastive learning strategy that aligns textual descriptions of tail diseases with image features, effectively mitigating performance degradation in diagnosis under long-tail distributions.

In the future, leveraging the pretraining characteristics of FMs, methods such as multi-task learning, cross-modal knowledge transfer, and active learning can be explored to enhance the recognition of low-frequency categories, thereby improving the robustness and applicability of models in real clinical settings.

### Privacy and security

Medical data are highly sensitive and strictly protected by various laws and regulations, making privacy and security critical challenges for medical FMs [7]. The training and inference processes often involve personally identifiable information, medical records, pathological data, and imaging, and any leakage can lead to severe consequences for both patients and healthcare institutions. During cross-institutional data sharing and model deployment, there are additional risks of unintended data retention, information leakage, and potential re-identification. Although collaborative training frameworks such as federated learning have partially mitigated privacy concerns associated with centralized data [13], they remain insufficient to fully prevent the inference of sensitive information from model parameters.

To address these concerns, researchers have proposed various privacy-preserving mechanisms, including differential privacy and de-identification techniques. These methods cover all stages from data preprocessing to model training and inference, establishing a multi-level and comprehensive privacy protection framework. As FMs become more deeply integrated into medical applications, building secure, trustworthy, and compliant privacy-preserving frameworks is a necessary prerequisite for their clinical deployment.

### Algorithmic fairness

Current medical FMs face multifaceted fairness challenges [12]. At the data level, significant distribution imbalances exist across gender, ethnicity, and geographic origin, leading to inadequate learning of pathological features in minority groups and resulting in systemic biases in disease detection. At the model level, biases not only originate from data but also are amplified by model architecture design, loss function, and optimization strategies, affecting the clinical fairness of the results [174]. At the evaluation level, existing metrics fail to reveal performance disparities across demographic groups and lack fine-grained fairness assessments aligned with medical requirements. This interconnected sequence of data bias, model amplification, and evaluation gaps significantly limits the generalizability and ethical compliance of medical FMs in clinical applications.

To address this issue, it is imperative to advance collaboratively in multiple directions, including the construction of diverse datasets, bias-aware model design, and the development of unified evaluation frameworks. Integrating technical standards with ethical governance mechanisms is essential to systematically enhance the fairness and trustworthiness of medical FMs, promoting their equitable, safe, and compliant deployment in real-world clinical settings.

### Uncertainty estimation

Uncertainty in medical image interpretation reflects the confidence level of model predictions, and its reliability is directly related to the safety of clinical decision-making. Uncertainty can be divided into 2 categories based on its source: caused by factors such as noise, artifacts, missing features, or anatomical differences, and epistemic uncertainty stemming from limitations in model structure and training data [32]. However, most FMs output only a single prediction probability, making it difficult to effectively quantify uncertainty. As a result, under distribution shifts or in complex lesion scenarios, these models often produce confidence estimates that exceed their actual accuracy, thereby increasing the risk of misclassification and misdiagnosis.

In medical FMs, this issue is particularly obvious, with several challenges remaining. First, some models suffer from insufficient calibration, where the predicted confidence does not align with the actual accuracy. Second, different pretraining strategies lead to varying calibration performance. General pretraining models often exhibit higher uncertainty in medical tasks, while domain-specific or self-supervised pretraining can mitigate this problem but cannot fully solve it. In addition, existing calibration and post-processing methods can improve confidence estimation to some extent, such as label smoothing, but it is difficult to eliminate the inherent uncertainty differences across models and data domains [175]. Overall, uncertainty estimation remains a critical challenge for the clinical adoption of medical FMs.

To address these challenges, existing research has employed probabilistic prediction, Bayesian neural networks, Monte Carlo dropout, and deep ensembles to quantify and assess uncertainty [176], providing reliable confidence indicators for clinical decision-making. Meanwhile, selecting high-quality, domain-specific pretraining models has been shown to significantly reduce uncertainty. For instance, several self-supervised pretraining models for medical imaging, such as RETFound [30] and UNI [95], demonstrate superior calibration.

Looking forward, the general representation capabilities of FMs should be fully leveraged, integrating multi-source data including imaging, text, and EHRs to improve uncertainty estimation. Additionally, uncertainty can be modeled and jointly evaluated at different levels, including pixel-level, lesion-level, and patient-level, enhancing the reliability and safety of models in real-world clinical applications.

### Interpretability

Interpretability in medical FMs refers to the ability of models to reveal its decision-making process and predictive rationale when performing complex medical tasks, which is essential for achieving trustworthy intelligent healthcare. Only when clinicians and researchers can understand and validate the model’s reasoning logic can its outputs be considered reliable for clinical adoption. Moreover, interpretability helps reduce the risk of erroneous predictions arising from reliance on artifacts, scanning protocols, or other nonpathological features, thereby enhancing model robustness and safety. However, medical FMs are typically characterized by highly complex structures, where nonlinear interactions between input and predicted outputs obscure the transparency of the decision process and make it difficult to trace the sources of key features [7]. In addition, interpretability needs diverge. Researchers emphasize transparency in model mechanisms and feature representations, while clinicians place greater importance on the alignment between model reasoning, medical knowledge, and anatomical priors—creating a semantic gap between the two.

To address these challenges, current research on explainable AI focuses on developing multi-level and multi-modal interpretability frameworks [11]. On one hand, techniques such as layer-wise relevance propagation, Shapley values, and attention mechanisms are employed to quantify and visualize the contributions of input features to prediction outcomes, thereby enabling both quantitative assessment and intuitive presentation of feature importance. On the other hand, clinical practice emphasizes enhancing medical reliability by integrating knowledge graphs, anatomical priors, and verifiable clinical trials, while researchers focus on providing reproducible and traceable explanations of model internals to support methodological improvements and model refinement.

Looking ahead, it is essential to establish a unified evaluation framework for the interpretability of medical FMs, which not only addresses the consistency of interpretability methods across different models and tasks but also incorporates quantitative metrics to assess the medical validity, stability, and clinical utility of explanations. Meanwhile, with advances in causal reasoning and multi-modal FMs [139], the interpretability of medical FMs is expected to evolve from mere result presentation toward a fundamental understanding of the reasoning mechanisms underlying model training and inference, thereby advancing them into a new stage of being “understandable and verifiable”, and providing transparent and trustworthy intelligence for complex medical tasks.

### Generalizability of model architecture

The architectural generalizability of medical FMs, meaning their ability to robustly transfer and adapt across multi-task and multi-modal medical scenarios, is critical for enabling intelligent healthcare deployment [174]. Achieving this requires unified modeling of heterogeneous data such as medical images, text, and audio, along with support for cross-task and cross-domain knowledge transfer to bridge semantic gaps between modalities and improve performance on complex clinical tasks.

Current research generally focuses on scaling models and incorporating Transformer architectures, exploring strategies such as multi-branch encoders, shared decoders, and cross-modal attention fusion. Combined with self-supervised pretraining, adaptive optimization, and regularization techniques [57], these approaches aim to enhance representation and learning efficiency across heterogeneous tasks and data. However, clinical practice still faces challenges including limited rare disease samples, cross-institution data distribution shifts, and inconsistent task semantics.

To further enhance architecture generalization, future work should focus on modular and composable designs that support knowledge disentanglement and transfer, integrate federated learning with generative modeling to mitigate data shifts [13], and incorporate causal reasoning alongside vision-language collaboration to improve interpretability and semantic consistency. With the development of cross-modal fusion mechanisms and transfer learning, medical FMs are expected to transition from specific architecture adaptation to a universal architecture paradigm, driving intelligent medical systems toward greater generality and clinical applicability.

### Downstream task adaptation

Medical FMs possess strong general representation capabilities and can be adapted to various medical image interpretation tasks such as organ segmentation, lesion detection, and disease classification through fine-tuning [14]. However, full fine-tuning of large-scale models is computationally and memory intensive, making it difficult to deploy in resource-constrained clinical settings. In addition, medical data are costly to annotate, limited in quantity, and subject to long-tail distributions [172] and privacy constraints. Significant differences across tasks further hinder effective model transfer.

Therefore, achieving efficient and low-cost fine-tuning while maintaining performance comparable to full training has become a key challenge for deploying medical FMs. Current approaches address this by using parameter-efficient tuning methods such as parameter freezing and low-rank adaptation (LoRA) [58], enabling task adaptation with minimal parameter updates. In parallel, strategies like data augmentation and KD are employed to enhance model generalization in low-data settings, aiming to build accurate and efficient fine-tuning frameworks.

### Clinical translation and deployment considerations

The transition of FMs from research to real-world clinical settings hinges on their medical significance and usability [16]: (a) Clinical demand-driven: Models must address actual clinical pain points (e.g., lesion detection, image–pathology correlation, and follow-up risk assessment) rather than merely improving algorithmic metrics. (b) Multi-center/multi-modal validation: Stability, generalizability, and fairness must be validated across devices, hospitals, and diverse population data to avoid single-center bias. (c) Real-world benefit evaluation: Beyond traditional metrics, translational value such as reduced physician workload, improved diagnostic consistency, or shortened diagnosis time should be quantified. For instance, PanDerm [98] proposed a multi-modal dermatology FM designed to achieve cross-domain advanced visual skills and integrate information from multiple imaging modalities. The study included generalization validation on 28 benchmark datasets addressing clinical needs such as skin cancer screening, risk stratification, differential diagnosis of common and rare skin diseases, lesion segmentation, longitudinal monitoring, as well as metastasis prediction and prognosis. Furthermore, 3 reader studies were conducted to quantify the real-world benefits PanDerm offers to physicians, demonstrating its potential clinical utility.

Clinical deployment refers to the engineering implementation phase where models are operationalized within medical institutions, focusing on technical and system integration. The specific workflow typically involves the following core steps: First, the model is containerized and deployed on local hospital servers or private cloud platforms. Next, standardized interface protocols are used to establish data connectivity and task coordination with hospital information systems. Subsequently, upon receiving medical imaging data, the model automatically performs inference and returns results (e.g., risk scores and segmentation masks) to hospital terminals for direct viewing and interactive use by clinicians. The entire process ensures the efficiency of model reasoning, the security and privacy of data, and the readability of clinical results.

### Computational efficiency

Computational efficiency and model lightweight are key challenges for the practical deployment of medical FMs in clinical settings [1]. Although these models demonstrate strong representational and task transfer capabilities, their development, training, and deployment often incur substantial resource costs. Medical imaging data, characterized by high resolution, 3D structure, and multi-modal complexity, significantly increase memory and computational demands.

This issue is especially pronounced in Transformer-based architectures, which typically involve long training times and high inference costs. While lightweight techniques such as KD and model pruning have achieved notable success in general vision tasks [93], their transferability to medical applications remains limited due to the higher accuracy and robustness requirements. Moreover, to ensure privacy protection, medical FMs often rely on federated learning frameworks, which require encrypted communication and distributed computation, further increasing resource consumption.

To develop efficient and practical medical FMs, future research can focus on several key directions. First, implementing dynamic computation mechanisms based on lesion regions may enable on-demand allocation of computational resources. Second, designing lightweight backbone networks can help balance performance and speed [93]. Third, optimizing parameter sharing strategies under federated learning, such as gradient sparsification and mixed-precision training, may reduce resource consumption [13]. With the continued advancement of deep learning in the medical domain, medical FMs are expected to achieve clinically deployable solutions that maintain diagnostic accuracy while improving efficiency and reducing computational demands.

### Generative models

To address the scarcity of medical data, generative models such as diffusion models [35] and generative adversarial networks [36] are widely used to synthesize high-quality medical images. However, medical image generation still faces challenges including poor anatomical and pathological consistency, sample homogenization caused by small datasets and long-tailed distributions, as well as privacy concerns, which limit its clinical application.

Subsequent research efforts could focus on incorporating anatomical knowledge graphs and spatial structural priors, using graph neural networks to model organ topology constraints [177]. Concurrently, developing federated generative frameworks alongside identity disentanglement mechanisms will enhance privacy protection [13]. Furthermore, medical image evaluation should balance perceptual consistency with diagnostic relevance, as conventional metrics such as mean squared error (MSE), PSNR, and SSIM inadequately reflect the nuanced visual quality of medical images. There is a critical need for novel evaluation metrics that integrate semantic understanding and subjective perceptual factors [178].

### Reinforcement learning

Reinforcement learning (RL) is a learning paradigm that optimizes strategies through environment interaction and reward feedback, and has recently been applied to medical image interpretation [179]. Compared to traditional supervised learning, RL is more suitable for complex medical tasks involving sequential decision-making and delayed feedback. However, its application in medical FMs faces several challenges. First, RL design is highly complex, and fine-tuning of the state space, action selection, and reward functions may cause training instability or failure to converge. Second, model performance is extremely sensitive to hyperparameter settings, and tuning relies heavily on experience without automated mechanisms. Furthermore, the lack of standardized RL frameworks and publicly available implementations in medical imaging hinders reproducibility and method dissemination. High-dimensional and complex medical image data further exacerbate issues such as high training costs, susceptibility to local optima, and slow convergence.

Despite these challenges, RL holds great potential for medical FMs. Future research may focus on the following areas: (a) hierarchical reinforcement learning (HRL), which decomposes complex tasks into subtasks and is suitable for modeling high-dimensional data such as 3D and 4D medical images [180]; (b) multi-task and transfer RL, enabling agents to transfer knowledge across related tasks to reduce training costs and improve generalization [181]; and (c) active RL combined with clinical feedback to enable rapid adaptation to clinical needs under limited data conditions [182]. In summary, although RL research in medical FMs is still in its early stages, its advantages in policy optimization and decision-making position it as a promising approach for future clinical applications.

### In-context learning

In-context learning (ICL) aims to assist in understanding new tasks by providing additional contextual information during inference and directly applying this understanding to predict outcomes for new inputs [53]. Unlike traditional transfer learning or meta-learning, it requires no additional training steps or updates to model weights. Instead, it relies on the knowledge and reasoning capabilities acquired during pretraining. InMeMo [183] enhances context through a learnable prompt mechanism and explores its potential for cross-task generalization and zero-shot inference. However, current methods still face significant challenges. On one hand, the effectiveness of visual ICL is highly dependent on the quality of prompts. On the other hand, variations in prompt integration and ordering can activate different levels of knowledge within the visual model, resulting in inconsistent outcomes and performance fluctuations [184]. Therefore, efficiently designing and optimizing prompt strategies to improve contextual information utilization remains a pressing issue.

Future research can focus on 2 aspects: first, improving the effectiveness of contextual prompts by evaluating the semantic similarity between candidate examples and the query image to optimize support set selection [136]; second, enhancing the accuracy and stability of inference results by generating diverse predictions through different arrangements of image subregions, designing causality-aware modules to remove suboptimal outputs via counterfactual reasoning, or introducing RL-based adaptive search mechanisms for dynamic prompt optimization [185].

### Knowledge graph

MKG is a structured framework that represents medical entities such as diseases, drugs, symptoms, and clinical indicators, along with their semantic relationships. It has been widely applied in medical image analysis and medical question answering [186]. Despite its potential, the application of MKG in real-world medical scenarios faces several challenges. Most existing MKG are built from limited structured or unstructured sources, resulting in incomplete coverage and potential semantic bias, which limits their generalizability in complex clinical settings. In addition, medical knowledge is inherently heterogeneous and multi-granular, involving diverse entity types and complex semantic relations. Traditional graph embedding methods struggle to balance semantic expressiveness and computational efficiency. Recent work such as LMKG [187] proposes automatic extraction of knowledge triples from multi-source heterogeneous medical texts and introduces a hierarchical entity alignment mechanism to enable fine-grained knowledge integration and unified management. This approach enhances the representational capacity of MKG.

Future research can advance in the following directions. First, integrating LLMs such as GPT-4 [3] can enhance semantic understanding of medical texts, thereby improving the accuracy and completeness of knowledge extraction [188]. Second, incorporating RL can support continuous optimization of knowledge validation, enhancing the accuracy and reliability of MKG. Third, the development of multi-modal knowledge graphs can support efficient integration of heterogeneous medical data and enable deeper modeling of complex semantic.

## Conclusion

FMs learn general representations through pretraining on large-scale, multi-source data and have demonstrated strong cross-task and cross-modal transfer capabilities. This paper provides a comprehensive review of the development of medical FMs in the field of medical image interpretation, with a focus on medical imaging data, evaluation metrics, universal FMs, and the proposed IPIU medical FM platform. It also surveys representative applications in medical image analysis and discusses key challenges and recent advances.

Although medical FMs have shown promising results across various scenarios, their practical deployment still faces multiple challenges, including data heterogeneity, limited interpretability, privacy concerns, and high computational demands. Looking ahead, future research may explore the potential of emerging techniques such as generative modeling, ICL, and RL while emphasizing the integration of medical knowledge, privacy-preserving mechanisms, and ethical considerations. Based on these perspectives, this paper outlines the key limitations of current approaches and highlights future directions to support the continued advancement and clinical adoption of medical FMs.

## Data Availability

No data were used for the research described in the article.

## References

[1] Awais M, Naseer M, Khan S, Anwer RM, Cholakkal H, Shah M, Yang MH, Khan FS. Foundation models defining a new era in vision: A survey and outlook. IEEE Trans Pattern Anal Mach Intell. 2025;47(4):2245–2264. 40030979 10.1109/TPAMI.2024.3506283

[2] Bommasani R, Hudson DA, Adeli E, Altman R, Arora S, von Arx S, Bernstein MS, Bohg J, Bosselut A, Brunskill E, et al. On the opportunities and risks of foundation models. arXiv. 2021. 10.48550/arXiv.2108.07258

[3] Kalyan KS. A survey of GPT-3 family large language models including ChatGPT and GPT-4. Nat Lang Process J. 2024;6: Article 100048.

[4] Wang Z, Wu Z, Agarwal D, Sun J. Medclip: Contrastive learning from unpaired medical images and text. In: *Proceedings of the Conference on Empirical Methods in Natural Language Processing*. 2022. p. 3876. 10.18653/v1/2022.emnlp-main.256 PMC11323634 39144675

[5] Wang C, Zhao J, Jiao L, Li L, Liu F, Yang S. When large language models meet evolutionary algorithms: Potential enhancements and challenges. Research. 2025;8:0646. 40151321 10.34133/research.0646 PMC11948732

[6] Binz M, Akata E, Bethge M, Brändle F, Callaway F, Coda-Forno J, Dayan P, Demircan C, Eckstein MK, Éltető N, et al. A foundation model to predict and capture human cognition. Nature. 2025;644:1002–1009. 40604288 10.1038/s41586-025-09215-4 PMC12390832

[7] Khan W, Leem S, See KB, Wong JK, Zhang S, Fang R. A comprehensive survey of foundation models in medicine. IEEE Rev Biomed Eng. 2025. 10.1109/RBME.2025.3531360 40031197

[8] Isensee F, Jaeger PF, Kohl SA, Petersen J, Maier-Hein KH. nnU-Net: A self-configuring method for deep learning-based biomedical image segmentation. Nat Methods. 2021;18(2):203–211. 33288961 10.1038/s41592-020-01008-z

[9] Singhal K, Azizi S, Tu T, Mahdavi SS, Wei J, Chung HW, Scales N, Tanwani A, Cole-Lewis H, Pfohl S, et al. Large language models encode clinical knowledge. Nature. 2023;620(7972):172–180. 37438534 10.1038/s41586-023-06291-2 PMC10396962

[10] Kumar D, Pratap B, Boora N, Kumar R, Sah NK. A comparative study of medical imaging modalities. Int J Radiol Sci. 2021;3(1):9–16.

[11] Hassija V, Chamola V, Mahapatra A, Singal A, Goel D, Huang K, Scardapane S, Spinelli I, Mahmud M, Hussain A. Interpreting black-box models: A review on explainable artificial intelligence. Cogn Comput. 2024;16(1):45–74.

[12] Jin R, Xu Z, Zhong Y, Yao Q, Qi D, Zhou SK, Li X. Fairmedfm: Fairness benchmarking for medical imaging foundation models. Adv Neural Inf Proces Syst. 2024;37:111318–111357.

[13] Li S, Miao D, Wu Q, Hong C, D’Agostino D, Li X, Ning Y, Shang Y, Wang Z, Liu M, et al. Federated learning in healthcare: A benchmark comparison of engineering and statistical approaches for structured data analysis. Health Data Sci. 2024;4:0196. 39635226 10.34133/hds.0196 PMC11615161

[14] VanBerlo B, Hoey J, Wong A. A survey of the impact of self-supervised pretraining for diagnostic tasks in medical X-ray, CT, MRI, and ultrasound. BMC Med Imaging. 2024;24(1):79. 38580932 10.1186/s12880-024-01253-0 PMC10998380

[15] He Y, Huang F, Jiang X, Nie Y, Wang M, Wang J, Chen H. Foundation model for advancing healthcare: Challenges, opportunities and future directions. IEEE Rev Biomed Eng. 2024;18:172–191. 10.1109/RBME.2024.3496744 39531565

[16] Moor M, Banerjee O, Abad ZS, Krumholz HM, Leskovec J, Topol EJ, Rajpurkar P. Foundation models for generalist medical artificial intelligence. Nature. 2023;616(7956):259–265. 37045921 10.1038/s41586-023-05881-4

[17] Zhang S, Metaxas D. On the challenges and perspectives of foundation models for medical image analysis. Med Image Anal. 2024;91: Article 102996. 37857067 10.1016/j.media.2023.102996

[18] Liu C, Jin Y, Guan Z, Li T, Qin Y, Qian B, Jiang Z, Wu Y, Wang X, Zheng YF, et al. Visual–language foundation models in medicine. Vis Comput. 2025;41(4):2953–2972.

[19] Ryu JS, Kang H, Chu Y, Yang S. Vision-language foundation models for medical imaging: A review of current practices and innovations. Biomed Eng Lett. 2025;15:809–830. 40917147 10.1007/s13534-025-00484-6 PMC12411343

[20] Savadjiev P, Chong J, Dohan A, Vakalopoulou M, Reinhold C, Paragios N, Gallix B. Demystification of AI-driven medical image interpretation: Past, present and future. Eur Radiol. 2019;29(3):1616–1624. 30105410 10.1007/s00330-018-5674-x

[21] Tian L, Greer H, Kwitt R, Vialard FX, San José Estépar R, Bouix S, Rushmore R, Niethammer M. unigradicon: A foundation model for medical image registration. In:International Conference on Medical Image Computing and Computer-Assisted Intervention. Springer; 2024. p. 749–760. 10.1007/978-3-031-72069-7_70 PMC13242914 42261488

[22] Yang Z, Wei T, Liang Y, Yuan X, Gao R, Xia Y, Zhou J, Zhang Y, Yu Z. A foundation model for generalizable cancer diagnosis and survival prediction from histopathological images. Nat Commun. 2025;16(1):2366. 40064883 10.1038/s41467-025-57587-y PMC11894166

[23] Shobayo O, Saatchi R. Developments in deep learning artificial neural network techniques for medical image analysis and interpretation. Diagnostics. 2025;15(9):1072. 40361891 10.3390/diagnostics15091072 PMC12071792

[24] Jiao L, Zhao J, Wang C, Liu X, Liu F, Li L, Shang R, Li Y, Ma W, Yang S. Nature-inspired intelligent computing: A comprehensive survey. Research. 2024;7:0442. 39156658 10.34133/research.0442 PMC11327401

[25] Liu C, Dong Y, Xiang W, Yang X, Su H, Zhu J, Chen Y, He Y, Xue H, Zheng S. A comprehensive study on robustness of image classification models: Benchmarking and rethinking. Int J Comput Vis. 2025;133:567–589.

[26] Gu P, Zhao Z, Wang H, Peng Y, Zhang Y, Sapkota N, Wang C, Chen DZ. Boosting medical image classification with segmentation foundation model. In: *2024 IEEE International Symposium on Biomedical Imaging (ISBI)*. IEEE; 2024. p. 1–5.

[27] Ma J, He Y, Li F, Han L, You C, Wang B. Segment anything in medical images. Nat Commun. 2024;15(1):654. 38253604 10.1038/s41467-024-44824-z PMC10803759

[28] Wan Z, Gao Y, Pang W, Ding D. VOILA: Complexity-aware universal segmentation of CT images by voxel interacting with language. Proc AAAI Conf Artif Intell. 2025;39(7):7482–7490.

[29] Liu J, Zhang Y, Chen JN, Xiao J, Lu Y, A Landman B, Yuan Y, Yuille A, Tang Y, Zhou Z. Clip-driven universal model for organ segmentation and tumor detection. In: *Proceedings of the IEEE/CVF International Conference on Computer Vision*. IEEE; 2023. p. 21152–21164. 10.1109/iccv51070.2023.01934 PMC13332747 42403563

[30] Zhou Y, Chia MA, Wagner SK, Ayhan MS, Williamson DJ, Struyven RR, Liu T, Xu M, Lozano MG, Woodward-Court P, et al. A foundation model for generalizable disease detection from retinal images. Nature. 2023;622(7981):156–163. 37704728 10.1038/s41586-023-06555-x PMC10550819

[31] Sun Y, Wang L, Li G, Lin W, Wang L. A foundation model for enhancing magnetic resonance images and downstream segmentation, registration and diagnostic tasks. Nat Biomed Eng. 2025;9(4):521–538. 39638876 10.1038/s41551-024-01283-7 PMC12360180

[32] Chen J, Liu Y, Wei S, Bian Z, Subramanian S, Carass A, Prince JL, Du Y. A survey on deep learning in medical image registration: New technologies, uncertainty, evaluation metrics, and beyond. Med Image Anal. 2024;100: Article 103385. 39612808 10.1016/j.media.2024.103385 PMC11730935

[33] Zhao H, Liu Z, Tang J, Gao B, Qin Q, Li J, Zhou Y, Yao P, Xi Y, Lin Y, et al. Energy-efficient high-fidelity image reconstruction with memristor arrays for medical diagnosis. Nat Commun. 2023;14(1):2276. 37081008 10.1038/s41467-023-38021-7 PMC10119144

[34] Wang J, Wang K, Yu Y, Lu Y, Xiao W, Sun Z, Liu F, Zou Z, Gao Y, Yang L, et al. Self-improving generative foundation model for synthetic medical image generation and clinical applications. Nat Med. 2025;31(2):609–617. 39663467 10.1038/s41591-024-03359-y

[35] Pan S, Abouei E, Wynne J, Chang CW, Wang T, Qiu RL, Li Y, Peng J, Roper J, Patel P, et al. Synthetic CT generation from MRI using 3D transformerbased denoising diffusion model. Med Phys. 2024;51(4):2538–2548. 38011588 10.1002/mp.16847 PMC10994752

[36] Chen Y, Lin H, Zhang W, Chen W, Zhou Z, Heidari AA, Chen H, Xu G. ICycle-GAN: Improved cycle generative adversarial networks for liver medical image generation. Biomed Signal Process Control. 2024;92: Article 106100.

[37] Wang X, Jiang Y, Yang S, Wang F, Zhang X, Wang W, Chen Y, Wu X, Xiang J, Li Y, et al. Foundation model for predicting prognosis and adjuvant therapy benefit from digital pathology in GI cancers. J Clin Oncol. 2025;43(32):3468–3481. 40168636 10.1200/JCO-24-01501

[38] Pai S, Bontempi D, Prudente V, Hadzic I, Sokač M, Chaunzwa TL, Bernatz S, Hosny A, Mak RH, Birkbak NJ, et al. Foundation models for quantitative biomarker discovery in cancer imaging. medRxiv. 2023. 10.1101/2023.09.04.23294952 PMC10957482 38523679

[39] Peng J, Zhou S, Yang L, Song Y, Zhang M, Zhou K, Xie F, Lin M, Zhang R, Chen T. Continually evolved multimodal foundation models for cancer prognosis. arXiv. 2025. 10.48550/arXiv.2501.18170

[40] Azad B, Azad R, Eskandari S, Bozorgpour A, Kazerouni A, Rekik I, Merhof D. Foundational models in medical imaging: A comprehensive survey and future vision. arXiv. 2023. 10.48550/arXiv.2310.18689

[41] Ou X, Chen X, Xu X, Xie L, Chen X, Hong Z, Bai H, Liu X, Chen Q, Li L, et al. Recent development in x-ray imaging technology: Future and challenges. Research. 2021;2021: Article 9892152. 35028585 10.34133/2021/9892152 PMC8724686

[42] Jiao J, Zhou J, Li X, Xia M, Huang Y, Huang L, Wang N, Zhang X, Zhou S, Wang Y, et al. Usfm: A universal ultrasound foundation model generalized to tasks and organs towards label efficient image analysis. Med Image Anal. 2024;96: Article 103202. 38788326 10.1016/j.media.2024.103202

[43] Nawaz K, Zanib A, Shabir I, Li J, Wang Y, Mahmood T, Rehman A. Skin cancer detection using dermoscopic images with convolutional neural network. Sci Rep. 2025;15(1):7252. 40021731 10.1038/s41598-025-91446-6 PMC11871080

[44] Dayarathna S, Islam KT, Uribe S, Yang G, Hayat M, Chen Z. Deep learning based synthesis of MRI, CT and PET: Review and analysis. Med Image Anal. 2024;92: Article 103046. 38052145 10.1016/j.media.2023.103046

[45] Hartsock I, Rasool G. Vision-language models for medical report generation and visual question answering: A review. Front Artif Intell. 2024;7:1430984. 39628839 10.3389/frai.2024.1430984 PMC11611889

[46] Maier-Hein L, Reinke A, Godau P, Tizabi MD, Buettner F, Christodoulou E, Glocker B, Isensee F, Kleesiek J, Kozubek M, et al. Metrics reloaded: Recommendations for image analysis validation. Nat Methods. 2024;21(2):195–212. 38347141 10.1038/s41592-023-02151-z PMC11182665

[47] Foody GM. Challenges in the real world use of classification accuracy metrics: From recall and precision to the Matthews correlation coefficient. PLOS ONE. 2023;18(10): Article e0291908. 37792898 10.1371/journal.pone.0291908 PMC10550141

[48] Rainio O, Teuho J, Klén R. Evaluation metrics and statistical tests for machine learning. Sci Rep. 2024;14(1):6086. 38480847 10.1038/s41598-024-56706-x PMC10937649

[49] Taha AA, Hanbury A. Metrics for evaluating 3D medical image segmentation: Analysis, selection, and tool. BMC Med Imaging. 2015;15(1): Article 29. 10.1186/s12880-015-0068-x PMC4533825 26263899

[50] Corbetta E, Bocklitz T. Multi-marker similarity enables reduced-reference and interpretable image quality assessment in optical microscopy. Research. 2025;8: Article 0783. 40689360 10.34133/research.0783 PMC12271742

[51] de Sousa RN, Oliveira SAF. Evaluating image synthesis: A modest review of techniques and metrics. In: *Conference on Graphics, Patterns and Images (SIBGRAPI)*. SBC; 2024. p. 82–87.

[52] Zhu K, Zheng Y, Chan KCG. Weighted brier score—An overall summary measure for risk prediction models with clinical utility consideration. Stat Biosci. 2025. 10.1007/s12561-025-09505-5 PMC12523994 41103457

[53] Butoi VI, Ortiz JJG, Ma T, Sabuncu MR, Guttag J, Dalca AV. Universeg: Universal medical image segmentation. In: *Proceedings of the IEEE/CVF International Conference on Computer Vision*. IEEE; 2023. p. 21438–21451.

[54] Liu J, Yang H, Zhou HY, Yu L, Liang Y, Yu Y, Zhang S, Zheng H, Wang S. Swin-UMamba†: Adapting Mamba-based vision foundation models for medical image segmentation. IEEE Trans Med Imaging. 2024;44(10):3898–3908. 10.1109/TMI.2024.3508698 40030346

[55] Zhuang J, Wu L, Wang Q, Fei P, Vardhanabhuti V, Luo L, Chen H. Mim: Mask in mask self-supervised pre-training for 3d medical image analysis. IEEE Trans Med Imaging. 2025;44(9):3727–3740. 40279226 10.1109/TMI.2025.3564382

[56] Gan Z, Li L, Li C, Wang L, Liu Z, Gao J. Vision-language pre-training: Basics, recent advances, and future trends. Found Trends Comput Graph Vis. 2022;14(3–4):163–352.

[57] Rani V, Kumar M, Gupta A, Sachdeva M, Mittal A, Kumar K. Self-supervised learning for medical image analysis: A comprehensive review. Evol Syst. 2024;15(4):1607–1633.

[58] Liu K, Price B, Kuen J, Fan Y, Wei Z, Figueroa L, Geras K, Fernandez-Granda C. Uncertainty-aware fine-tuning of segmentation foundation models. Adv Neural Inf Proces Syst. 2024;37:53317–53389.

[59] Zhang J, Huang J, Jin S, Lu S. Vision-language models for vision tasks: A survey. IEEE Trans Pattern Anal Mach Intell. 2024;46(8):5625–5644. 38408000 10.1109/TPAMI.2024.3369699

[60] Huang Z, Wang H, Deng Z, Ye J, Su Y, Sun H, He J, Gu Y, Gu L, Zhang S, et al. Stu-net: Scalable and transferable medical image segmentation models empowered by large-scale supervised pre-training. arXiv. 2023. 10.48550/arXiv.2304.06716

[61] Zhai X, Kolesnikov A, Houlsby N, Beyer L. Scaling vision transformers. In: *Proceedings of the IEEE/CVF Conference on Computer Vision and Pattern Recognition*. IEEE; 2022. p. 12104–12113.

[62] Du Y, Bai F, Huang T, Zhao B. Segvol: Universal and interactive volumetric medical image segmentation. Adv Neural Inf Proces Syst. 2024;37:110746–110783.

[63] Wu L, Zhuang J, Chen H. Voco: A simple-yet-effective volume contrastive learning framework for 3d medical image analysis. In: *Proceedings of the IEEE/CVF Conference on Computer Vision and Pattern Recognition*. IEEE; 2024. p. 22873–22882.

[64] Sowrirajan H, Yang J, Ng AY, Rajpurkar P. Moco pretraining improves representation and transferability of chest x-ray models. In: *Medical Imaging with Deep Learning*. PMLR; 2021. p. 728–744.

[65] Wang Z, Liu C, Zhang S, Dou Q. Foundation model for endoscopy video analysis via largescale self-supervised pre-train. In:International Conference on Medical Image Computing and Computer-Assisted Intervention. Springer; 2023. p. 101–111.

[66] He K, Chen X, Xie S, Li Y, Dollár P, Girshick R. Masked autoencoders are scalable vision learners. In: *Proceedings of the IEEE/CVF Conference on Computer Vision and Pattern Recognition*. IEEE; 2022. p. 16000–16009.

[67] Gupta A, Osman I, Shehata MS, Braun WJ, Feldman RE. MedMAE: A self-supervised backbone for medical imaging tasks. Computation. 2025;13(4):88.

[68] Liu H, Wei D, Lu D, Sun J, Wang L, Zheng Y. M3AE: Multimodal representation learning for brain tumor segmentation with missing modalities. Proc AAAI Conf Artif Intell. 2023;37(2):1657–1665.

[69] Chen Z, Agarwal D, Aggarwal K, Safta W, Balan MM, Brown K. Masked image modeling advances 3d medical image analysis. In: *Proceedings of the IEEE/CVF Winter Conference on Applications of Computer Vision*. IEEE; 2023. p. 1970–1980.

[70] Cai Z, Lin L, He H, Cheng P, Tang X. Uni4eye++: A general masked image modeling multi-modal pre-training framework for ophthalmic image classification and segmentation. IEEE Trans Med Imaging. 2024;43(12):4412–4429. 10.1109/TMI.2024.3422102 38954581

[71] Zhou Z, Sodha V, Pang J, Gotway MB, Liang J. Models genesis. Med Image Anal. 2021;67: Article 101840. 33188996 10.1016/j.media.2020.101840 PMC7726094

[72] Wang J, Xie G, Huang Y, Lyu J, Zheng F, Zheng Y, Jin Y. FedMed-GAN: Federated domain translation on unsupervised cross-modality brain image synthesis. Neurocomputing. 2023;546: Article 126282.

[73] Zhang T, Wei D, Zhu M, Gu S, Zheng Y. Self-supervised learning for medical image data with anatomy-oriented imaging planes. Med Image Anal. 2024;94: Article 103151. 38527405 10.1016/j.media.2024.103151

[74] Tang Y, Yang D, Li W, Roth HR, Landman B, Xu D, Nath V, Hatamizadeh A. Self-supervised pre-training of swin transformers for 3d medical image analysis. In: *Proceedings of the IEEE/CVF Conference on Computer Vision and Pattern Recognition*. IEEE; 2022. p. 20730–20740.

[75] Wang G, Wu J, Luo X, Liu X, Li K, Zhang S. Mis-fm: 3d medical image segmentation using foundation models pretrained on a large-scale unannotated dataset. arXiv. 2023. 10.48550/arXiv.2306.16925

[76] Zhang K, Zhou R, Adhikarla E, Yan Z, Liu Y, Yu J, Liu Z, Chen X, Davison BD, Ren H, et al. A generalist vision–language foundation model for diverse biomedical tasks. Nat Med. 2024;30(11):3129–3141. 39112796 10.1038/s41591-024-03185-2 PMC12581140

[77] Chen Z, Du Y, Hu J, Liu Y, Li G, Wan X, Chang TH. Mapping medical image-text to a joint space via masked modeling. Med Image Anal. 2024;91: Article 103018. 37976867 10.1016/j.media.2023.103018

[78] Zhou HY, Lian C, Wang L, Yu Y. Advancing radiograph representation learning with masked record modeling. arXiv. 2023. 10.48550/arXiv.2301.13155

[79] Xie Y, Gu L, Harada T, Zhang J, Xia Y, Wu Q. Rethinking masked image modelling for medical image representation. Med Image Anal. 2024;98: Article 103304. 39173412 10.1016/j.media.2024.103304

[80] Huang SC, Shen L, Lungren MP, Yeung S. Gloria: A multimodal global-local representation learning framework for label-efficient medical image recognition. In: *Proceedings of the IEEE/CVF International Conference on Computer Vision*. IEEE; 2021. p. 3942–3951.

[81] Zhang Y, Jiang H, Miura Y, Manning CD, Langlotz CP. Contrastive learning of medical visual representations from paired images and text. In: *Machine Learning for Healthcare Conference*. PMLR; 2022. p. 2–25.

[82] Zhang X, Wu C, Zhang Y, Xie W, Wang Y. Knowledge-enhanced visual-language pretraining on chest radiology images. Nat Commun. 2023;14(1):4542. 37507376 10.1038/s41467-023-40260-7 PMC10382552

[83] Liu C, Cheng S, Chen C, Qiao M, Zhang W, Shah A, Bai W, Arcucci R. M-flag: Medical vision-language pre-training with frozen language models and latent space geometry optimization. In:International Conference on Medical Image Computing and Computer-Assisted Intervention. Springer; 2023. p. 637–647.

[84] Shu C, Zhu Y, Tang X, Xiao J, Chen Y, Li X, Zhang Q, Lu Z. Miter: Medical image–text joint adaptive pretraining with multi-level contrastive learning. Expert Syst Appl. 2024;238: Article 121526.

[85] Liu B, Lu Z, Wang Y. Towards medical vision-language contrastive pre-training via study-oriented semantic exploration. In: *Proceedings of the 32nd ACM International Conference on Multimedia*. ACM; 2024. p. 4861–4870.

[86] Moon JH, Lee H, Shin W, Kim YH, Choi E. Multi-modal understanding and generation for medical images and text via vision-language pre-training. IEEE J Biomed Health Inform. 2022;26(12):6070–6080. 36121943 10.1109/JBHI.2022.3207502

[87] Wu C, Zhang X, Zhang Y, Wang Y, Xie W. Medklip: Medical knowledge enhanced language-image pre-training for x-ray diagnosis. In: *Proceedings of the IEEE/CVF International Conference on Computer Vision*. IEEE; 2023. p. 21372–21383.

[88] Huang W, Li C, Zhou HY, Yang H, Liu J, Liang Y, Zheng H, Zhang S, Wang S. Enhancing representation in radiography-reports foundation model: A granular alignment algorithm using masked contrastive learning. Nat Commun. 2024;15(1):7620. 39223122 10.1038/s41467-024-51749-0 PMC11369198

[89] Liu J, Zhou HY, Li C, Huang W, Yang H, Liang Y, Shi G, Zheng H, Wang S. Mlip: Medical language-image pre-training with masked local representation learning. In: *2024 IEEE International Symposium on Biomedical Imaging (ISBI)*. IEEE; 2024. p. 1–5.

[90] Wu R, Zhang C, Zhang J, Zhou Y, Zhou T, Fu H. MM-Retinal: Knowledge-enhanced foundational pretraining with fundus image-text expertise. In:International Conference on Medical Image Computing and Computer-Assisted Intervention. Springer; 2024. p. 722–732.

[91] Wu B, Xie Y, Zhang Z, Phan MH, Chen Q, Chen L, Wu Q. MMCLIP: Cross-modal attention masked modelling for medical language-image pre-training. arXiv. 2024. 10.48550/arXiv.2407.19546

[92] Pai S, Bontempi D, Hadzic I, Prudente V, Sokač M, Chaunzwa TL, Bernatz S, Hosny A, Mak RH, Birkbak NJ, et al. Foundation model for cancer imaging biomarkers. Nat Mach Intell. 2024;6(3):354–367. 38523679 10.1038/s42256-024-00807-9 PMC10957482

[93] Lu S, Chen Y, Chen Y, Li P, Sun J, Zheng C, Zou Y, Liang B, Li M, Jin Q, et al. General lightweight framework for vision foundation model supporting multi-task and multi-center medical image analysis. Nat Commun. 2025;16(1):2097. 40025028 10.1038/s41467-025-57427-z PMC11873151

[94] Islam NU, Ma D, Pang J, Velan SS, Gotway M, Liang J. Foundation X: Integrating classification, localization, and segmentation through lock-release pretraining strategy for chest x-ray analysis. In: *2025 IEEE/CVF Winter Conference on Applications of Computer Vision (WACV)*. IEEE; 2025. p. 3647–3656.

[95] Chen RJ, Ding T, Lu MY, Williamson DF, Jaume G, Song AH, Chen B, Zhang A, Shao D, Shaban M, et al. Towards a general-purpose foundation model for computational pathology. Nat Med. 2024;30(3):850–862. 38504018 10.1038/s41591-024-02857-3 PMC11403354

[96] Gao Z, Zhang G, Liang H, Liu J, Ma L, Wang T, Guo Y, Chen Y, Yan Z, Chen X, et al. A lung CT foundation model facilitating disease diagnosis and medical imaging. medRxiv. 2025. 10.1101/2025.01.13.25320295 PMC12764816 41339572

[97] He Y, Guo P, Tang Y, Myronenko A, Nath V, Xu Z, Yang D, Zhao C, Simon B, Belue M, et al. VISTA3D: A unified segmentation foundation model for 3D medical imaging. In: *Proceedings of the Computer Vision and Pattern Recognition Conference*. 2025. p. 20863–20873.

[98] Yan S, Yu Z, Primiero C, Vico-Alonso C, Wang Z, Yang L, Tschandl P, Hu M, Ju L, Tan G, et al. A multimodal vision foundation model for clinical dermatology. Nat Med. 2025;31:2691–2702. 40481209 10.1038/s41591-025-03747-y PMC12353815

[99] Lee J, Yoon W, Kim S, Kim D, Kim S, So CH, Kang J. BioBERT: A pre-trained biomedical language representation model for biomedical text mining. Bioinformatics. 2020;36(4):1234–1240. 31501885 10.1093/bioinformatics/btz682 PMC7703786

[100] Eslami S, Meinel C, De Melo G. Pubmedclip: How much does clip benefit visual question answering in the medical domain? In: *Findings of the Association for Computational Linguistics*. EACL; 2023. p. 1181–1193.

[101] Bannur S, Hyland S, Liu Q, Perez-Garcia F, Ilse M, Castro DC, Boecking B, Sharma H, Bouzid K, Thieme A, et al. Learning to exploit temporal structure for biomedical visionlanguage processing. In: *Proceedings of the IEEE/CVF Conference on Computer Vision and Pattern Recognition*. IEEE; 2023. p. 15016–15027.

[102] Chen Q, Hong Y. Medblip: Bootstrapping language-image pre-training from 3d medical images and texts. In: *Proceedings of the Asian Conference on Computer Vision*. 2024. p. 2404–2420.

[103] Nie Y, He S, Bie Y, Wang Y, Chen Z, Yang S, Cai Z, Wang H, Wang X, Luo L, et al. ConceptCLIP: Towards trustworthy medical AI via concept enhanced contrastive language-image pre-training. arXiv. 2025. 10.48550/arXiv.2501.15579

[104] Zhao Z, Zhang Y, Wu C, Zhang X, Zhang Y, Wang Y, Xie W. One model to rule them all: Towards universal segmentation for medical images with text prompts. arXiv. 2023. 10.48550/arXiv.2312.17183

[105] Lu MY, Chen B, Williamson DF, Chen RJ, Liang I, Ding T, Jaume G, Odintsov I, Le LP, Gerber G, et al. A visual-language foundation model for computational pathology. Nat Med. 2024;30(3):863–874. 38504017 10.1038/s41591-024-02856-4 PMC11384335

[106] Oh Y, Seifert R, Cao Y, Clement C, Ferdinandus J, Lapa C, Liebich A, Amon M, Enke J, Song S, et al. Developing a PET/CT foundation model for cross-modal anatomical and functional imaging. arXiv. 2025. 10.48550/arXiv.2503.02824

[107] Blankemeier L, Cohen JP, Kumar A, Van Veen D, Gardezi SJ, Paschali M, Chen Z, Delbrouck JB, Reis E, Truyts C, et al. Merlin: A vision language foundation model for 3d computed tomography. Research Square. 2024. 10.21203/rs.3.rs-4546309/v1

[108] Zhao T, Gu Y, Yang J, Usuyama N, Lee HH, Kiblawi S, Naumann T, Gao J, Crabtree A, Abel J, et al. A foundation model for joint segmentation, detection and recognition of biomedical objects across nine modalities. Nat Methods. 2025;22(1):166–176. 39558098 10.1038/s41592-024-02499-w

[109] Xiang J, Wang X, Zhang X, Xi Y, Eweje F, Chen Y, Li Y, Bergstrom C, Gopaulchan M, Kim T, et al. A vision–language foundation model for precision oncology. Nature. 2025;638(8051):769–778. 39779851 10.1038/s41586-024-08378-w PMC12295649

[110] Shentu J, Al Moubayed N. CXR-IRGen: An integrated vision and language model for the generation of clinically accurate chest X-ray image-report pairs. In: *Proceedings of the IEEE/CVF Winter Conference on Applications of Computer Vision*. IEEE; 2024. p. 5212–5221.

[111] Bluethgen C, Chambon P, Delbrouck JB, Van Der Sluijs R, Połacin M, Zambrano Chaves JM, Abraham TM, Purohit S, Langlotz CP, Chaudhari AS. A vision–language foundation model for the generation of realistic chest x-ray images. Nat Biomed Eng. 2025;9(4):494–506. 39187663 10.1038/s41551-024-01246-y PMC11861387

[112] Wu C, Zhang X, Zhang Y, Hui H, Wang Y, Xie W. Towards generalist foundation model for radiology by leveraging web-scale 2d&3d medical data. Nat Commun. 2025;16(1):7866. 40849424 10.1038/s41467-025-62385-7 PMC12375113

[113] Guo F, Guan R, Li Y, Liu Q, Wang X, Yang C, Wang J. Foundation models in bioinformatics. Natl Sci Rev. 2025;12(4): Article nwaf028. 40078374 10.1093/nsr/nwaf028 PMC11900445

[114] Wornow M, Xu Y, Thapa R, Patel B, Steinberg E, Fleming S, Pfeffer MA, Fries J, Shah NH. The shaky foundations of large language models and foundation models for electronic health records. npj Digit Med. 2023;6(1):135. 37516790 10.1038/s41746-023-00879-8 PMC10387101

[115] Sivarajkumar S, Zhang H, Ji Y, Bilalpur M, Wu X, Li C, Kwak MG, Visweswaran S, Wang Y. Generative foundation model for structured and unstructured electronic health records. arXiv. 2025. 10.48550/arXiv.2508.16054 PMC13269122 42312196

[116] Fallahpour A, Alinoori M, Ye W, Cao X, Afkanpour A, Krishnan A. Ehrmamba: Towards generalizable and scalable foundation models for electronic health records. arXiv. 2024. 10.48550/arXiv.2405.14567

[117] Zhu W, Tang H, Zhang H, Rajamohan HR, Huang SL, Ma X, Chaudhari A, Madaan D, Almahmoud E, Chopra S, et al. Predicting risk of Alzheimer’s diseases and related dementias with AI foundation model on electronic health records. medRxiv. 2024. 10.1101/2024.04.26.24306180

[118] Hou W, Wang J, Lin Q, Wang X, Huang L. Improving clinical foundation models with multi-modal learning and domain adaptation for chronic disease prediction. IEEE J Biomed Health Inform. 2025. 10.1109/JBHI.2025.3595140 40758490

[119] Liu F, Zhou H, Wang K, Yu Y, Gao Y, Sun Z, Liu S, Sun S, Zou Z, Li Z, et al. MetaGP: A generative foundation model integrating electronic health records and multimodal imaging for addressing unmet clinical needs. Cell Rep Med. 2025;6(4): Article 102056. 40187356 10.1016/j.xcrm.2025.102056 PMC12047458

[120] Rim B, Sung NJ, Min S, Hong M. Deep learning in physiological signal data: A survey. *Sensors*. 2020;20(4):969. 10.3390/s20040969 PMC7071412 32054042

[121] Jiang WB, Fu X, Ding Y, Guan C. Towards robust multimodal physiological foundation models: Handling arbitrary missing modalities. arXiv. 2025. 10.48550/arXiv.2504.19596

[122] McKeen K, Masood S, Toma A, Rubin B, Wang B. Ecg-fm: An open electrocardiogram foundation model. arXiv. 2024. 10.48550/arXiv.2408.05178 PMC12530324 41113504

[123] Phukan OC, Behera SR, Akhtar MM, Buduru AB, Sharma R. Beyond speech and more: Investigating the emergent ability of speech foundation models for classifying physiological time-series signals. arXiv. 2024. 10.48550/arXiv.2410.12645

[124] Wu C, Wang H, Zhang X, Zhang C, Bu J. Efficient personalized adaptation for physiological signal foundation model. In: *Forty-Second International Conference on Machine Learning*.

[125] Dalla-Torre H, Gonzalez L, Mendoza-Revilla J, Lopez Carranza N, Grzywaczewski AH, Oteri F, Dallago C, Trop E, de Almeida BP, Sirelkhatim H, et al. Nucleotide transformer: Building and evaluating robust foundation models for human genomics. Nat Methods. 2025;22(2):287–297. 39609566 10.1038/s41592-024-02523-z PMC11810778

[126] Hao M, Gong J, Zeng X, Liu C, Guo Y, Cheng X, Wang T, Ma J, Zhang X, Song L. Large-scale foundation model on single-cell transcriptomics. Nat Methods. 2024;21(8):1481–1491. 38844628 10.1038/s41592-024-02305-7

[127] Cui H, Wang C, Maan H, Pang K, Luo F, Duan N, Wang B. scGPT: Toward building a foundation model for single-cell multi-omics using generative AI. Nat Methods. 2024;21(8):1470–1480. 38409223 10.1038/s41592-024-02201-0

[128] Kaplan J, McCandlish S, Henighan T, Brown TB, Chess B, Child R, Gray S, Radford A, Wu J, Amodei D. Scaling laws for neural language models. arXiv. 2020. 10.48550/arXiv.2001.08361

[129] Zhang S, Liu Q, Usuyama N, Wong C, Naumann T, Poon H. Exploring scaling laws for EHR foundation models. arXiv. 2025. 10.48550/arXiv.2505.22964

[130] Yang W, Zhang H, Tan W, Sun Y, Yan B. A self-supervised paradigm for data efficient medical foundation model pre-training: V-information optimization framework. arXiv. 2024. 10.48550/arXiv.2408.07107

[131] Cardoso MJ, Li W, Brown R, Ma N, Kerfoot E, Wang Y, Murrey B, Myronenko A, Zhao C, Yang D, et al. Monai: An open-source framework for deep learning in healthcare. arXiv. 2022. 10.48550/arXiv.2211.02701

[132] Hatamizadeh A, Nath V, Tang Y, Yang D, Roth HR, Xu D. Swin unetr: Swin transformers for semantic segmentation of brain tumors in mri images. In:International MICCAI Brainlesion Workshop. Springer; 2021. p. 272–284.

[133] Xing Z, Ye T, Yang Y, Liu G, Zhu L. Segmamba: Long-range sequential modeling mamba for 3d medical image segmentation. arXiv. 2024. https://arxiv.org/abs/2401.13560. 10.1109/TMI.2025.3589797 40679879

[134] Chen T, Ding C, Zhu L, Xu T, Wang Y, Ji D, Zang Y, Li Z. xLSTM-UNet can be an effective backbone for 2D & 3D biomedical image segmentation better than its Mamba counterparts. In: *2024 IEEE EMBS International Conference on Biomedical and Health Informatics (BHI)*. IEEE; 2024. p. 1–8.

[135] Wang H, Guo S, Ye J, Deng Z, Cheng J, Li T, Chen J, Su Y, Huang Z, Shen Y, et al. SAM-Med3D: A vision foundation model for general-purpose segmentation on volumetric medical images. IEEE Trans Neural Networks Learn Syst. 2025;36(10):17599–17612. 10.1109/TNNLS.2025.3586694 40742874

[136] Gao J, Lao Q, Kang Q, Liu P, Du C, Li K, Zhang L. Boosting your context by dual similarity checkup for in-context learning medical image segmentation. IEEE Trans Med Imaging. 2024;44(1):310–319. 10.1109/TMI.2024.3440311 39115986

[137] Lewis P, Perez E, Piktus A, Petroni F, Karpukhin V, Goyal N, Küttler H, Lewis M, Yih WT, Rocktäschel T, et al. Retrieval-augmented generation for knowledge-intensive nlp tasks. Adv Neural Inf Proces Syst. 2020;33:9459–9474.

[138] Xia P, Zhu K, Li H, Wang T, Shi W, Wang S, Zhang L, Zou J, Yao H. MMed-RAG: Versatile multimodal RAG system for medical vision language models. arXiv. 2024. 10.48550/arXiv.2410.13085

[139] Jiao L, Wang Y, Liu X, Li L, Liu F, Ma W, Guo Y, Chen P, Yang S, Hou B. Causal inference meets deep learning: A comprehensive survey. Research. 2024;7:0467. 39257419 10.34133/research.0467 PMC11384545

[140] Yang Z, Wang J, Ye X, Tang Y, Chen K, Zhao H, Torr PH. Language-aware vision transformer for referring segmentation. IEEE Trans Pattern Anal Mach Intell. 2024;47(7):5238–5255. 10.1109/TPAMI.2024.3468640 39321010

[141] Wu J, Wang Z, Hong M, Ji W, Fu H, Xu Y, Xu M, Jin Y. Medical sam adapter: Adapting segment anything model for medical image segmentation. Med Image Anal. 2025;102: Article 103547. 40121809 10.1016/j.media.2025.103547

[142] Li H, Liu H, Hu D, Wang J, Oguz I. Promise: Prompt-driven 3d medical image segmentation using pretrained image foundation models. In: *2024 IEEE International Symposium on Biomedical Imaging (ISBI)*. IEEE; 2024. p. 1–5. 10.1109/isbi56570.2024.10635207 PMC12128788 40458423

[143] Zhang X, Ou N, Basaran BD, Visentin M, Qiao M, Gu R, Matthews PM, Liu Y, Ye C, Bai W. A foundation model for lesion segmentation on brain mri with mixture of modality experts. IEEE Trans Med Imaging. 2025;44(6):2594–2604. 40031588 10.1109/TMI.2025.3540809

[144] Wang X, Zhao J, Marostica E, Yuan W, Jin J, Zhang J, Li R, Tang H, Wang K, Li Y, et al. A pathology foundation model for cancer diagnosis and prognosis prediction. Nature. 2024;634(8035):970–978. 39232164 10.1038/s41586-024-07894-z PMC12186853

[145] Xu L, Ni Z, Sun H, Li H, Zhang S. A foundation model for generalizable disease diagnosis in chest X-ray images. arXiv. 2024. 10.48550/arXiv.2410.08861

[146] Vaid A, Jiang J, Sawant A, Lerakis S, Argulian E, Ahuja Y, Lampert J, Charney A, Greenspan H, Narula J, et al. A foundational vision transformer improves diagnostic performance for electrocardiograms. npj Digit Med. 2023;6(1):108. 37280346 10.1038/s41746-023-00840-9 PMC10242218

[147] Ding X, Chu Y, Pi R, Wang H, Li X. HiA: Towards Chinese multimodal LLMs for comparative high-resolution joint diagnosis. In:International Conference on Medical Image Computing and Computer-Assisted Intervention. Springer; 2024. p. 575–586.

[148] Du J, Guo J, Zhang W, Yang S, Liu H, Li H, Wang N. Ret-clip: A retinal image foundation model pre-trained with clinical diagnostic reports. In:International Conference on Medical Image Computing and Computer-Assisted Intervention. Springer; 2024. p. 709–719.

[149] Tanno R, Barrett DG, Sellergren A, Ghaisas S, Dathathri S, See A, Welbl J, Lau C, Tu T, Azizi S, et al. Collaboration between clinicians and vision– Language models in radiology report generation. Nat Med. 2025;31(2):599–608. 39511432 10.1038/s41591-024-03302-1 PMC11835717

[150] Jin H, Che H, Lin Y, Chen H. Promptmrg: Diagnosis-driven prompts for medical report generation. Proc AAAI Conf Artif Intell. 2024;38(3):2607–2615.

[151] Zhang X, Shi Y, Ji J, Zheng C, Qu L. MEPNet: Medical entity-balanced prompting network for brain CT report generation. Proc AAAI Conf Artif Intell. 2025;39(24):25940–25948.

[152] Zhao Z, Wang S, Gu J, Zhu Y, Mei L, Zhuang Z, Cui Z, Wang Q, Shen D. Chatcad+: Towards a universal and reliable interactive cad using llms. IEEE Trans Med Imaging. 2024;43(11):3755–3766. 38717880 10.1109/TMI.2024.3398350

[153] Liu A, Guo Y, Yong J, Xu F. Multi-grained radiology report generation with sentencelevel image-language contrastive learning. IEEE Trans Med Imaging. 2024;43(7):2657–2669. 38437149 10.1109/TMI.2024.3372638

[154] Gu T, Liu D, Li Z, Cai W. Complex organ mask guided radiology report generation. In: *Proceedings of the IEEE/CVF Winter Conference on Applications of Computer Vision*. IEEE; 2024. p. 7995–8004.

[155] Zhan C, Peng P, Wang H, Wang G, Lin Y, Chen T, Wang H. UnICLAM: Contrastive representation learning with adversarial masking for unified and interpretable medical vision question answering. Med Image Anal. 2025;101: Article 103464. 39847954 10.1016/j.media.2025.103464

[156] Liu Y, Chen B, Wang S, Lu G, Zhang Z. Deep fuzzy multi-teacher distillation network for medical visual question answering. IEEE Trans Fuzzy Syst. 2024;32(10):5413–5427.

[157] Moor M, Huang Q, Wu S, Yasunaga M, Dalmia Y, Leskovec J, Zakka C, Reis EP, Rajpurkar P. Med-flamingo: A multimodal medical few-shot learner. In: *Machine Learning for Health (ML4H)*. PMLR; 2023. p. 353–367.

[158] Thawakar OC, Shaker AM, Mullappilly SS, Cholakkal H, Anwer RM, Khan S, Laaksonen J, Khan F. XrayGPT: Chest radiographs summarization using large medical vision-language models. In: *Proceedings of the 23rd Workshop on Biomedical Natural Language Processing*. 2024. p. 440–448.

[159] Li C, Wong C, Zhang S, Usuyama N, Liu H, Yang J, Naumann T, Poon H, Gao J. Llava-med: Training a large language-and-vision assistant for biomedicine in one day. Adv Neural Inf Proces Syst. 2023;36:28541–28564.

[160] Chen J, Wang X, Ji K, Gao A, Jiang F, Chen S, Zhang H, Song D, Xie W, Kong C, et al. Huatuogpt-ii, one-stage training for medical adaption of llms. arXiv. 2023. 10.48550/arXiv.2311.09774

[161] Liu S, Li K, Zhao M, Tian Y, Li B, Zhou S, Li H, Yang F. M^3^-Med: A benchmark for multi-lingual, multi-modal, and multihop reasoning in medical instructional video understanding. arXiv. 2025. 10.48550/arXiv.2507.04289

[162] Huang Z, Bianchi F, Yuksekgonul M, Montine TJ, Zou J. A visual–language foundation model for pathology image analysis using medical twitter. Nat Med. 2023;29(9):2307–2316. 37592105 10.1038/s41591-023-02504-3

[163] Gong X, Koduru BH, Zhai Y, Liu S, Xi N, Tang X, Zhang Y, Lhakpa T, Tian Y, Sun Y, et al. EndoAssistant: A large-scale vision-language dataset for endoscopic surgery understanding from open-source videos.

[164] Montana-Brown N, Saeed SU, Abdulaal A, Dowrick T, Kilic Y, Wilkinson S, Gao J, Mashar M, He C, Stavropoulou A, et al. Saramis: Simulation assets for robotic assisted and minimally invasive surgery. Adv Neural Inf Proces Syst. 2023;36:26121–26134.

[165] Zeng T, Loza Galindo G, Hu J, Valdastri P, Jones D. Realistic surgical image dataset generation based on 3d gaussian splatting. In: *International Conference on Medical Image Computing and Computer-Assisted Intervention*. Springer; 2024. p. 510–519.

[166] Kondo S. Zeal: Surgical skill assessment with zero-shot tool inference using unified foundation model. arXiv. 2024. 10.48550/arXiv.2407.02738

[167] Cui B, Islam M, Bai L, Ren H. Surgical-dino: Adapter learning of foundation models for depth estimation in endoscopic surgery. Int J Comput Assist Radiol Surg. 2024;19(6):1013–1020. 38459402 10.1007/s11548-024-03083-5 PMC11178563

[168] Chen T, Yuan K, Srivastav V, Navab N, Padoy N. Text-driven adaptation of foundation models for few-shot surgical workflow analysis. Int J Comput Assist Radiol Surg. 2025;20(6):1175–1183. 40244318 10.1007/s11548-025-03341-0 PMC12167271

[169] Schmidgall S, Kim JW, Kuntz A, Ghazi AE, Krieger A. General-purpose foundation models for increased autonomy in robot-assisted surgery. Nat Mach Intell. 2024;6(11):1275–1283.

[170] Wang G, Yang G, Du Z, Fan L, Li X. ClinicalGPT: Large language models finetuned with diverse medical data and comprehensive evaluation. arXiv. 2023. 10.48550/arXiv.2306.09968

[171] Xia P, Zhu K, Li H, Zhu H, Li Y, Li G, Zhang L, Yao H. Rule: Reliable multimodal rag for factuality in medical vision language models. arXiv. 2024. 10.48550/arXiv.2407.05131

[172] Wu Z, Guo K, Luo E, Wang T, Wang S, Yang Y, Zhu X, Ding R. Medical long-tailed learning for imbalanced data: Bibliometric analysis. Comput Methods Prog Biomed. 2024;247: Article 108106. 10.1016/j.cmpb.2024.108106 38452661

[173] Zheng Q, Zhao W, Wu C, Zhang X, Dai L, Guan H, Li Y, Zhang Y, Wang Y, Xie W. Large-scale long-tailed disease diagnosis on radiology images. Nat Commun. 2024;15(1):10147. 39578456 10.1038/s41467-024-54424-6 PMC11584732

[174] Yang Y, Zhang H, Gichoya JW, Katabi D, Ghassemi M. The limits of fair medical imaging AI in real-world generalization. Nat Med. 2024;30(10):2838–2848. 38942996 10.1038/s41591-024-03113-4 PMC11485237

[175] Huang H, Razavian N. Uncertainty of vision medical foundation models. In: *ICLR Workshop: Quantify Uncertainty and Hallucination in Foundation Models: The Next Frontier in Reliable AI*.

[176] Lambert B, Forbes F, Doyle S, Dehaene H, Dojat M. Trustworthy clinical AI solutions: A unified review of uncertainty quantification in deep learning models for medical image analysis. Artif Intell Med. 2024;150: Article 102830. 38553168 10.1016/j.artmed.2024.102830

[177] Hammernik K, Küstner T, Yaman B, Huang Z, Rueckert D, Knoll F, Akçakaya M. Physics-driven deep learning for computational magnetic resonance imaging: Combining physics and machine learning for improved medical imaging. IEEE Signal Process Mag. 2023;40(1):98–114. 37304755 10.1109/msp.2022.3215288 PMC10249732

[178] Al Najjar Y. Comparative analysis of image quality assessment metrics: MSE, PSNR, SSIM and FSIM. Int J Sci Res. 2024;13(3):110–114.

[179] Elmekki H, Islam S, Alagha A, Sami H, Spilkin A, Zakeri E, Zanuttini AM, Bentahar J, Kadem L, Xie WF, et al. Comprehensive review of reinforcement learning for medical ultrasound imaging. Artif Intell Rev. 2025;58(9): Article :284. 40567264 10.1007/s10462-025-11268-w PMC12185671

[180] Zhang J, Cheng M, Cheng Q, Shen X, Wan Y, Zhu J, Liu M. Hierarchical medical image report adversarial generation with hybrid discriminator. Artif Intell Med. 2024;151: Article 102846. 38547777 10.1016/j.artmed.2024.102846

[181] Li C, Dong S, Yang S, Hu Y, Ding T, Li W, Gao Y. Multi-task multi-agent reinforcement learning with interaction and task representations. IEEE Trans Neural Networks Learn Syst. 2025;36(7):13431–13445. 10.1109/TNNLS.2024.3475216 39475745

[182] Patel U, Patel V. Active learning-based hyperspectral image classification: A reinforcement learning approach. J Supercomput. 2024;80(2):2461–2486.

[183] Zhang J, Wang B, Li L, Nakashima Y, Nagahara H. Instruct me more! random prompting for visual in-context learning. In: *Proceedings of the IEEE/CVF Winter Conference on Applications of Computer Vision*. IEEE; 2024. p. 2597–2606.

[184] Sun Y, Chen Q, Wang J, Wang J, Li Z. Exploring effective factors for improving visual in-context learning. IEEE Trans Image Process. 2025;34:2147–2160. 40168207 10.1109/TIP.2025.3554410

[185] Suo W, Lai L, Sun M, Zhang H, Wang P, Zhang Y. Rethinking and improving visual prompt selection for in-context learning segmentation. In:European Conference on Computer Vision. Springer; 2024. p. 18–35.

[186] Zuo K, Jiang Y, Mo F, Lio P. Kg4diagnosis: A hierarchical multi-agent llm framework with knowledge graph enhancement for medical diagnosis. In: *AAAI Bridge Program on AI for Medicine and Healthcare*. PMLR; 2025. p. 195–204.

[187] Yang P, Wang H, Huang Y, Yang S, Zhang Y, Huang L, Zhang Y, Wang G, Yang S, He L, et al. LMKG: A large-scale and multi-source medical knowledge graph for intelligent medicine applications. Knowl-Based Syst. 2024;284: Article 111323.

[188] Cui H, Lu J, Wang S, Xu R, Ma W, Yu S, Yu Y, Kan X, Fu T, Ling C, et al. A survey on knowledge graphs for healthcare: Resources, application progress, and promise. In: *ICML 3rd Workshop on Interpretable Machine Learning in Healthcare (IMLH)*. 2023.

[189] Jayasumana S, Ramalingam S, Veit A, Glasner D, Chakrabarti A, Kumar S. Rethinking fid: Towards a better evaluation metric for image generation. In: *Proceedings of the IEEE/CVF Conference on Computer Vision and Pattern Recognition*. IEEE; 2024. p. 9307–9315.

[190] Duffy BA, Zhang W, Tang H, Zhao L, Law M, Toga AW, Kim H. Retrospective correction of motion artifact affected structural MRI images using deep learning of simulated motion. In: *Medical Imaging with Deep Learning*. 2018.

[191] Cox DR. The regression analysis of binary sequences. J R Stat Soc Ser B Stat Methodol. 1958;20(2):215–232.

[192] Chen T, Guestrin C. Xgboost: A scalable tree boosting system. In: *Proceedings of the 22nd ACM SIGKDD International Conference on Knowledge Discovery and Data Mining*. ACM; 2016. p. 785–794.

[193] Cortes C, Vapnik V. Support-vector networks. *Mach Learn*. 1995;20(3):273–297.

[194] Cover T, Hart P. Nearest neighbor pattern classification. IEEE Trans Inf Theory. 1967;13(1):21–27.

[195] Ke G, Meng Q, Finley T, Wang T, Chen W, Ma W, Ye Q, Liu TY. Lightgbm: A highly efficient gradient boosting decision tree. Adv Neural Inf Proces Syst. 2017;30.

[196] Friedman JH. Greedy function approximation: A gradient boosting machine. Ann Stat. 2001;29(5):1189–1232.

[197] Lewis DD. Naive (Bayes) at forty: The independence assumption in information retrieval. In:European Conference on Machine Learning. Springer; 1998. p. 4–15.

[198] Chen J, Liao K, Wan Y, Chen DZ, Wu J. Danets: Deep abstract networks for tabular data classification and regression. Proc AAAI Conf Artif Intell. 2022;36(4):3930–3938.

[199] Huang X, Khetan A, Cvitkovic M, Karnin Z. Tabtransformer: Tabular data modeling using contextual embeddings. arXiv. 2020. 10.48550/arXiv.2012.06678

[200] Arik SO, Pfister T. Tabnet: Attentive interpretable tabular learning. Proc AAAI Conf Artif Intell. 2021;35(8):6679–6687.

[201] Gorishniy Y, Rubachev I, Khrulkov V, Babenko A. Revisiting deep learning models for tabular data. Adv Neural Inf Proces Syst. 2021;34:18932–18943.

[202] Joseph M, Raj H. GANDALF: Gated adaptive network for deep automated learning of features. arXiv. 2022. 10.48550/arXiv.2207.08548

[203] Myronenko A. 3D MRI brain tumor segmentation using autoencoder regularization. In: *Brainlesion: Glioma, Multiple Sclerosis, Stroke and Traumatic Brain Injuries: 4th International Workshop, BrainLes 2018, Held in Conjunction with MICCAI 2018, Granada, Spain, September 16, 2018, Revised Selected Papers, Part II 4*. Springer; 2019. p. 311–320.

[204] Lee HH, Bao S, Huo Y, Landman BA. 3d ux-net: A large kernel volumetric convnet modernizing hierarchical transformer for medical image segmentation. arXiv. 2022. 10.48550/arXiv.2209.15076 PMC13597054 42775365

[205] Roy S, Koehler G, Ulrich C, Baumgartner M, Petersen J, Isensee F, Jaeger PF, Maier-Hein KH. Mednext: Transformer-driven scaling of convnets for medical image segmentation. In:International Conference on Medical Image Computing and Computer-Assisted Intervention. Springer; 2023. p. 405–415.

[206] Hatamizadeh A, Tang Y, Nath V, Yang D, Myronenko A, Landman B, Roth HR, Xu D. Unetr: Transformers for 3d medical image segmentation. In: *Proceedings of the IEEE/CVF Winter Conference on Applications of Computer Vision*. IEEE; 2022. p. 574–584.

[207] Yu X, Yang Q, Zhou Y, Cai LY, Gao R, Lee HH, Li T, Bao S, Xu Z, Lasko TA, et al. Unest: Local spatial representation learning with hierarchical transformer for efficient medical segmentation. Med Image Anal. 2023;90: Article 102939. 37725868 10.1016/j.media.2023.102939 PMC11229077

[208] Li R, Xu L, Xie K, Song J, Ma X, Chang L, Yan Q. Dht-net: Dynamic hierarchical transformer network for liver and tumor segmentation. IEEE J Biomed Health Inform. 2023;27(7):3443–3454. 37079414 10.1109/JBHI.2023.3268218

[209] Yang J, Jiao L, Shang R, Liu X, Li R, Xu L. Ept-net: Edge perception transformer for 3d medical image segmentation. IEEE Trans Med Imaging. 2023;42(11):3229–3243. 37216246 10.1109/TMI.2023.3278461

[210] Liao W, Zhu Y, Wang X, Pan C, Wang Y, Ma L. Lightm-unet: Mamba assists in lightweight unet for medical image segmentation. arXiv. 2024. 10.48550/arXiv.2403.05246

[211] Ma J, Li F, Wang B. U-mamba: Enhancing long-range dependency for biomedical image segmentation. arXiv. 2024. 10.48550/arXiv.2401.04722

[212] Grattafiori A, Dubey A, Jauhri A, Pandey A, Kadian A, Al-Dahle A, Letman A, Mathur A, Schelten A, Vaughan A, et al. The llama 3 herd of models. arXiv. 2024. 10.48550/arXiv.2407.21783

[213] Yang A, Li A, Yang B, Zhang B, Hui B, Zheng B, Yu B, Gao C, Huang C, Lv C, et al. Qwen3 technical report. arXiv. 2025. 10.48550/arXiv.2505.09388

[214] Chen J, Cai Z, Ji K, Wang X, Liu W, Wang R, Hou J, Wang B. Huatuogpt-o1, towards medical complex reasoning with llms. arXiv. 2024. 10.48550/arXiv.2412.18925

[215] Guo D, Yang D, Zhang H, Song J, Zhang R, Xu R, Zhu Q, Ma S, Wang P, Bi X, et al. Deepseek-r1: Incentivizing reasoning capability in llms via reinforcement learning. arXiv. 2025. 10.48550/arXiv.2501.12948 PMC12443585 40962978

[216] Wu J, Deng W, Li X, Liu S, Mi T, Peng Y, Xu Z, Liu Y, Cho H, Choi CI, et al. Medreason: Eliciting factual medical reasoning steps in llms via knowledge graphs. arXiv. 2025. 10.48550/arXiv.2504.00993

