# Supplementary material for: Foundation Models Meet Medical Image Interpretation
Source: Research (Wash D C). 2026 Feb 27;9:1024. doi: 10.34133/research.1024 (PMC12946388; doi:10.34133/research.1024)
Supplement: Supplementary 1 — Tables S1 to S16 [file research.1024.f1.pdf]

Foundation Models Meet Medical Image Interpretation

Supplement Database

Table S1: Summary of 2D image datasets widely used in medical foundation models.

Task	Anatomical Region	Imaging method	Dataset
Classification	Whole body	Pathology	NAFLD
	Head and Neck	Pathology	OSCC
	Thorax	CT	COVID-CT, SARS-COV-2 Ct-Scan, Chest CT-Scan images
		X-Ray	COVID-19 CHEST X-RAY, COVIDGR, MIAS, CoronaHack
		Ultrasound	BUSI
		Pathology	BreaKHis, MIDOG++, LungHist700
	Abdomen	Pathology	Gleason 2019, NuCLS, PANDA, RenalCell
		Endoscope	Colonoscopic, LIMUC, The Nerthus Dataset, CP-CHILD
	Eyes	OCT	Retinal OCT-C8, OCT 2017
		Fundus Images	RFMiD 2.0, MuReD, REFUGE, ToxoFundus
Segmentation	Head and Neck	Ultrasound	TN-SCUI 2020, DDTI, TG3K, TN3K
	Thorax	Ultrasound	Breast Ultrasound Dataset B, BUSI
	Abdomen	Endoscope	Kvasir-SEG, CVC-ClinicDB, CholecSeg8k, EAD 2019
		Pathology	EBHI-Seg, SegPANDA200, CRAG
	Multi-organs	Multi-modality	IMIS-Benchmark
Detection	Abdomen	Endoscope	LDPolypVideo, LIMUC
Registration	Thorax	Pathology	ANHIR
Reconstruction	Abdomen	Endoscope	EndoSLAM, EndoMappe, C3VD
Med-VQA	Multi-regions	Multi-modality	PMC-OA, PMC-VQA, VQA-RAD, VQA-Med, SLAKE, PathVQA, MIMIC-Diff-VQA, Gemex
Report Generation	Thorax	Multi-modality	MIMIC-CXR, CheXpert, MIMIC-NLE, CXR-PRO, MS-CXR, IU X-ray
	Multi-regions	Multi-modality	ROCOv2

Table S2: Summary of 3D image datasets widely used in medical foundation models.

Task	Anatomical Region	Imaging method	Dataset
Classification	Multi-regions	Multi-modality	RP3D-DiagDS 🔗
Detection	Thorax	CT	LUNA16 🔗
	Skeleton	CT	RibFrac 2020 🔗
		MR	lumbosacral spine MRI 🔗
Segmentation	Whole body	CT	TotalSegmentator v1.0 🔗, CT-ORG , AutoPET 🔗
		MR	TotalSegmentator MRI 🔗
	Head and Neck	CT	SegRap2023 🔗, HaN-Seg 🔗, PDDCA 🔗, HECKTOR 2022 🔗
	Brain	CT	INSTANCE 2022 🔗
		MR	FeTA 2022 🔗, iSeg 🔗, BraTS21🔗, ISLES22 🔗, ATLAS v2.0 🔗, WMH 🔗
	Thorax	CT	ATM22 🔗, Parse 2022 🔗, SegTHOR 🔗, PleThora 🔗, FUMPE 🔗
		MR	ACDC 🔗, LAScarQS 2022 🔗, MyoPS 2020 🔗, CMRxMotion 🔗
		Ultrasound	MVSeg-3DTEE 2023 🔗, TDSC-ABUS2023 🔗
	Abdomen	CT	FLARE 2022 🔗, WORD 🔗, AbdomenCT-1K 🔗, 3D-IRCADB 🔗, LiTS 🔗, KiTS23 🔗, BTCV 🔗, MSD 🔗, PanSegData 🔗, MOOD2022 🔗
		MR	AMOS 2022 🔗, ATLAS 🔗, SPPIN 🔗, CHAOS 🔗, PROMISE12 🔗
	Skeleton	CT	VerSe 🔗, CTSpine1K 🔗, CTPelvic1K 🔗
		MR	SPIDER 🔗
	Vessel	CTA	SEG.A. 🔗, KiPA22 🔗, ImageCAS 🔗
Registration	Brain	MR	L2R-OASIS 🔗
Med-VQA	Multi-regions	Multi-modality	3D-RAD 🔗
Report Generation	Multi-regions	Multi-modality	RP3D-Caption 🔗

Note: 🔗 indicates a hyperlink to the corresponding original paper or official dataset source.

Table S3: Summary of extended multi-modality datasets widely used in medical foundation models.

Task	Dataset	Imaging method	Data Information
Structured EHRs	MIMIC-II 🔗	2001-2008 EHRs	25,328 ICU admissions with laboratory data, interventions, vital signs, clinical notes, and waveform recordings.
	MIMIC-III 🔗	2001-2012 EHRs	53,423 adult patients admitted to the BIDMC ICU from June 2001 to October 2012, 7,870 critically ill neonates admitted from 2001 to 2008.
	MIMIC-IV 🔗	2008-2019 EHRs	Clinical data on more than 190,000 patients and 450,000 hospitalizations admitted to BIDMC, including Hospital module(demographics, measurement, and related information), ICU module, and Notes module.
	eICU Dataset 🔗	2014-2015 EHRs	Multi-center intensive care dataset comprising 200,859 admissions of approximately 140,000 unique patients.
Physiological Signals	PhysioNet 2021 🔗	ECG	131,149 12-lead ECG records from nine databases.
	MIMIC-IV-ECG 🔗	ECG	Approximately 800,000 diagnostic 12-lead ECGs from nearly 160,000 unique patients, each 10 seconds long and sampled at 500 Hz.
	WESAD 🔗	Physiological signals, Motion signals	Comprising physiological and motion signals from 15 subjects using chest (700 Hz) and wrist (4–64 Hz) sensors for stress and affect detection.
	Sleep-EDF 🔗	EEG, EOG, chin EMG	197 whole-night PolySomnoGraphic sleep recordings, containing EEG, EOG, chin EMG, and event markers.
	DREAMER 🔗	EEG, ECG	Comprising EEG and ECG signals recorded from 23 participants during emotion elicitation via audiovisual stimuli.
	MIT-BIH arrhythmia dataset 🔗	ECG	Including over 4,000 dynamic 24-hour ECG recordings from 47 subjects, totaling 109,500 heartbeats, with 30% abnormal beats.
	SEED-VII 🔗	EEG, EOG, ECG	A multi-modal emotion dataset comprising EEG, EOG, and ECG signals from 20 subjects recorded at 1000 Hz.
	TUEG 🔗	EEG, EOG, ECG, EMG	Contains 26,846 clinical EEG recordings with occasional EOG, ECG, and EMG signals, sampled at 250–1024 Hz.
	DEAP 🔗	EEG, EOG, EMG	EEG, EOG, and EMG signals from 32 participants watching 40 one-minute music video clips, sampled at 512 Hz.
	GX 🔗	EEG, ECG, EOG, tES	A multi-modal human-subject dataset combining EEG, physiological signals (ECG, EOG), and tES data from 783 trials across 62 sessions.
	SPH 🔗	ECG	A large-scale multi-label 12-lead ECG dataset from 24,666 patients with 25,770 records.
Bioinformatics	hECA 🔗	scRNA-seq	A unified human cell atlas (hECA) integrating 1,093,299 cells from 116 studies across 38 organs and 146 cell types.
	Baron dataset 🔗	scRNA-seq	A single-cell transcriptomic dataset of over 12,000 pancreatic cells from humans and mice.
	The 1000G dataset 🔗	Whole-Genome Sequencing	A whole-genome sequencing(WGS) dataset of 3,202 human samples from 26 populations, including 602 trios.
	DeepSEA 🔗	DNA sequence	Comprising 2.4 million sequences of 1,000 nucleotides each, annotated with 919 chromatin features.

Note: 🔗 indicates a hyperlink to the corresponding original paper or official dataset source.

Table S4: Summary of representative pretraining models in medical foundation models.

	Category	Method	Public	Param	Vision Encoder	Datasets	Adaptation
Vision Pretraining Model	Supervised Learning	STU-Net [1]	[Arxiv'23]	14M-1.4B	nnU-Net	TotalSegmentator	FPFT
		MedSAM [2]	[Nature Communications'24]	-	ViT	1,570,263 image-mask pairs	ZST
		SegVol [3]	[NeurIPS'24]	180M	3D ViT/CLIP	25 Datasets, 96k CTs	ZST
	Contrastive Learning	MoCo-CXR [4]	[PMLR'21]	-	ResNet18, DenseNet121	CheXpert	FPFT
		Endo-FM [5]	[MICCAI'23]	121M	Video Transformer	33K Video Clips	PEFT
		VoCo [6]	[CVPR'24]	62.2M	3D UNet, Swin-UNETR	1.6k CT Scans	FPFT
	Generative Learning	Models Genesis [7]	[MIA'21]	16.32M	3D U-Net	LUNA16, ChestX-ray14	FPFT
		M^{3}AE [8]	[AAAI'23]	-	3D U-Net	BraTS2018, BraTS21	FPFT
		MIM-Med3D [9]	[WACV'23]	-	ViT,SwinTransformer,VAN	BTCV, BraTS21, TCIA-COVID19	FPFT
		FedMed-GAN [10]	[Neurocomputing'23]	-	GAN	IXI, BraTS21	FPFT
		Uni4Eye++ [11]	[TMI'24]	-	ViT	mmOphth-v2	FPFT
		Mim [12]	[TMI'25]	-	Swin Transformers	10,502 CT Scans	FPFT
		MedMAE [13]	[MDPI'25]	-	ViT	LUMID 2 Million+ Images	FPFT
	Predictive Learning	Zhang et al. [14]	[MIA'24]	-	U-Net, AlexNet	CMR, Knee MRI	FPFT
	Hybrid Learning	Swin UNETR [15]	[MICCAI'21]	62.2M	Swin Transformer	BraTS21	FPFT
		MIS-FM [16]	[arXiv'23]	-	CNN+Transformer	110k Unannotated 3D CT	FPFT

	Category	Method	Public	Text Encoder	Vision Encoder	Datasets	Adaptation
Vision-language Pretraining Model	Mask Modeling	MRM [17]	[ICLR'23]	Transformer	ViT	MIMIC-CXR	FPFT
		MedIM [18]	[MIA'24]	BioClinicalBERT	ViT	MIMIC-CXR-JPG	FPFT
	Contrastive Learning	GLoRIA [19]	[ICCV'21]	BioClinicalBERT	ResNet-50	CheXpert, RSNA Pneumonia, SIIM Pneumothorax	ZST
		MedCLIP [20]	[EMNLP'22]	BioClinicalBERT	Swin Transformer	CheXpert, MIMIC-CXR, COVID, RSNA Pneumonia	ZST
		ConVIRT [21]	[PMLR'22]	BERT	ResNet50	MIMIC-CXR, 48k Musculoskeletal Image-text Pairs	ZST
		KAD [22]	[Nature Communications'23]	PubMedBERT	ResNet-50, ViT	MIMIC-CXR	ZST
		M-FLAG [23]	[MICCAI'23]	CXR-BERT	ResNet50	MIMIC-CXR	FPFT
		MITER [24]	[ESWA'23]	ClinicalBERT	ViT	900k Unlabeled Radiographs	FPFT
		Liu et al. [25]	[ACM MM'24]	BioClinicalBERT	ResNet-50	MIMIC-CXR	ZST
	Hybrid VLP	MedViLL [26]	[JBHI'22]	BERT	ResNet-50	MIMIC-CXR	FPFT
		MedKLIP [27]	[ICCV'23]	ClinicalBERT	ResNet-50	MIMIC-CXR v2	ZST
		MaCo [28]	[Nature Communications'24]	BERT	ViT	MIMIC-CXR v2	ZST
		MLIP [29]	[ISBI'24]	BioClinicalBert	ViT-B	MIMIC-CXR	ZST
		MM-Retinal [30]	[MICCAI'24]	BioClinicalBert	ResNet50	MM-Retinal, 4.3K image-text pairs	ZST
		MMCLIP [31]	[arXiv'24]	Transformer	ViT	MIMIC-CXR, PadChest	ZST

Public: Indicates methods reported in publicly accessible research papers and preprints.

Param: Number of trainable parameters.

Adaptation: *ZST:* Zero-shot transfer; *PEFT:* Parameter-efficient fine-tuning; *FPFT:* Full-parameter fine-tuning.

Note: 🔗 indicates a hyperlink to the corresponding original paper or or official dataset download page.

Table S5: Summary of representative medical vision foundation models.

Category	Method	Public	Param	Vision Encoder	Data Information	Task
Universal VFMs	Pai et al. [32]	[Nature machine intelligence'24]	-	3D ResNet50	11,467 Radiographic Lesions	Classification,Prognosis Prediction
	VFMGL [33]	[Nature Communications'25]	11M	ResNet18, U-Net	EC dataset*, CAMELYON17 🔗, TNBC 🔗, I2CVB 🔗, PROMISE12 🔗, MoNuSAC2018 🔗, MoNuSAC2020 🔗, NCI-ISBI13 🔗	Classification,Segmentation
	Foundation X [34]	[WACV'25]	-	Swin Transformer	11 Chest X-Ray Datasets	Classification,Localization,Segmentation
Modality-universal VFMs	UNI [35]	[Nature Medicine'24]	-	ViT	over 100,000 Diagnostic H&E-stained WSIs	Classification,Quantitative Evaluation
	Usfm [36]	[MIA'24]	-	ViT-B	3M-US Database, 2187k+ US Images	Classification,Segmentation,Image Enhancement
	LCTfound [37]	[medrxiv'25]	200M	U-Net+ViT	105,184 Lung CT Scans	Segmentation,Diagnosis,Prognosis Prediction, Generation,Reconstruction,Image Enhancement, 3D Modeling,Treatment Response Prediction
Organ/Task-universal VFMs	UniverSeg [38]	[ICCV'23]	1.18M	U-Net	MegaMedical	Segmentation
	SAMAug-C [39]	[ISBI'24]	-	SAM	ISIC 2017 🔗, Vitiligo 🔗, ExtCRC 🔗	Segmentation
	UniGradICON [40]	[MICCAI'24]	-	3D U-Net	COPDGenev 🔗, OAI 🔗, HCP 🔗, L2R-Abdomen 🔗	Registration
	VISTA3D [41]	[CVPR'25]	-	SegResNet	11454 CT scans	Segmentation
	RETFound [42]	[NPJ digital medicine'23]	-	ViT	904,170 CFPs and 736,442 OCT scans	Detection
	PanDerm [43]	[Nature Medicine'25]	-	ViT-Large	2,149,706 Unlabeled Skin Images	Segmentation,Prognosis Prediction,Diagnosis, Screening,Risk Assessment,Change Detection, ...

Note: * indicates that the dataset is private. 🔗 indicates a hyperlink to the corresponding original paper or official dataset download page.

Table S6: Summary of representative medical vision-language foundation models.

Category	Method	Public	Vision Encoder	Text Encoder	Data Information	Task
Encoder-only	PubMedCLIP [44]	[EACL'23]	ViT,ResNet-50	CLIP	VQA-RAD 🔗, SLAKE 🔗	Med-VQA
	BioViL-T [45]	[CVPR'23]	CNN–Transformer	BERT	MIMIC-CXR v2 🔗, MS-CXR-T 🔗	Progression Classification,Phrase Grounding, Medical Report Generation
	MedBLIP [46]	[ACCV'24]	ViT-G/14	FLAN-T5,BioGPT,BioMedLM	30,000 3D Image Scans	Classification,Med-VQA
	ConceptCLIP [47]	[arXiv'25]	ViT	PubMedBERT	MedConcept-23M	Diagnosis,Medical Report Generation,Med-VQA, Text-to-image Retrieval,Image-to-text Retrieval
Encoder-Decoder	CLIP-Driven [48]	[ICCV'23]	-	CLIP	3,410 CT Scans	Segmentation,Detection
	SAT [49]	[arXiv'23]	3D U-Net	BERT	22K 3D Medical Image Scans	Segmentation
	RoentGen [50]	[Nature Biomedical Engineering'24]	U-Net	CLIP ViT-L/14	MIMIC-CXR 🔗, CheXpert 🔗, VinDr-CXR 🔗	Generation
	Merlin [51]	[Research Square'24]	ResNet152	Clinical Longformer	1.5w+ CT Scans,180w+ EHRs Codes, 600w+ Report Tokens	Classification,Segmentation,Cross-modal Retrieval, Prognosis Prediction,Radiology Report Generation
	CRX-IRGen [52]	[WACV'24]	CLIP	CLIP	MIMIC-CXR 🔗	Generation
	CONCH [53]	[Nature Medicine'24]	ViT	-	1.17M Image-caption Pairs	Classification,Segmentation,Captioning, Text-to-image Retrieval,Image-to-text Retrieval
	BiomedGPT [54]	[Nature Medicine'24]	ViT	BERT	MIMIC-CXR 🔗, IU X-ray 🔗, PathVQA 🔗, VQA-RAD 🔗, PMC-OA 🔗	Classification,Radiology Report Generation, Radiology VQA,Radiology Report Summarizes
	MUSK [55]	[Nature'25]	ViT	Transformer	TCGA , QUILT-1M 🔗, PathAsst 🔗	Image-to-text Retrieval,Text-to-image Retrieval, Med-VQA,Classification,Molecular Biomarker Prediction
	BiomedParse [56]	[Nature Methods'25]	Focal21, SAM-ViT	PubMedBERT,Transformer	BiomedParseData	Segmentation,Detection,Recognition
	FratMAE [57]	[arXiv'25]	ViT	BERT	AutoPET III 🔗	Segmentation,Ann Arbor Staging
	VOILA [58]	[AAAI'25]	-	CLIP	Totalsegmentator v2 🔗, WORD 🔗, AMOS 🔗, BTCV 🔗, AbdomenCT-1K 🔗, LiTS 🔗, Pancreas-CT 🔗	Segmentation
	RadFM [59]	[Nature Communication'25]	ViT	MedLLaMA-13B	MedMD, 13 million 2D Images and 615k 3D Scans	Diagnosis,Med-VQA, Medical Report Generation

Note: 🔗 indicates a hyperlink to the corresponding original paper or official dataset download page.

Table S7: Summary of representative medical extended multi-modal foundation models.

Category	Method	Encoder	Data Information	Task
Structured EHRs	EHRMamba [60]	Mamba	MIMIC-IV 🔗	Mortality,Length of Stay,Readmission,Hypertension, Fluid Disorders,Lipoid Metabolism Disorders Prediction
	Zhu et al. [61]	Transformer	1,288,333 patients with 587 million medical concept tokens from NYU Langone Health EHRs	the AD/ADRD/MCI risks in 1 year and 5 year
	GDP [62]	Transformer	MIMIC-IV 🔗	Heart failure (HF), Type 2 diabetes mellitus (T2DM), 30-day Readmission Prediction,Discharge Summaries Generation
	MetaGP [63]	Transformer	8 million EHRs, biomedical literature, medical textbooks	Rare Disease Diagnosis, Emergency Conditions Identification, Medical Report Generation
	MsHeCare [64]	Transformer	eICU Dataset 🔗, Changde Dataset*	Chronic Disease Predict (Multi-label Classification)
Physiological Signals	ECG-FM [65]	Transformer	PhysioNet 2021 🔗, MIMIC-IV-ECG 🔗	UHN-ECG Interpretation,MIMIC-IV-ECG Machine Reads,UHN-ECG Reduced LVEF
	Phukan et al. [66]	Transformer	WESAD 🔗	Physiological signal (ECG, EMG, EDA) Classification and Stress Recognition
	PhysioPFM [67]	Transformer	Sleep-EDF dataset 🔗, DREAMER 🔗, MIT-BIH arrhythmia dataset 🔗, FOG dataset 🔗	Sleep-state Detection,Emotion Detection, Arrhythmia diagnosis,Freezing of Gaits Detection
	PhysioOmni [68]	Transformer	TUEG 🔗, DEAP 🔗, Sleep-EDF dataset 🔗, CAP 🔗, GX 🔗	Emotion Recognition,Sleep Stage Classification, Motor Prediction,Mental Workload Detection
Bioinformatics	scFoundation [69]	Transformer	over 50 million human scRNA-seq data	Gene Expression Enhancement,Tissue Drug Response Prediction, Single-cell Perturbation Prediction,Single-cell Drug Response Classification, Cell Type Annotation,Gene Module Inference
	scGPT [70]	Transformer	CELLxGENE🔗	ScRNA-seq Integration with Batch Correction,Cell Type Annotation, Multi-omic Integration,Perturbation Prediction,Gene Regulatory Network Inference
	Nucleotide Transformer [71]	Transformer	3,202 human genomes, 850 genomes from diverse species	Predicting Varied Molecular Phenotypes,Promoter Tasks, Histone Modification and Enhancer Tasks

Note: * indicates that the dataset is private. 🔗 indicates a hyperlink to the corresponding original paper or official dataset download page.

Table S8: Summary of Whole-Body Dataset within the IPIU Large-Scale Medical Imaging Dataset.

Dimensions	Modality	Dataset	Task	Targets	Data Size	File Format	Public
3D	CT	Total Segmentator v1.0	Organ Segmentation	104	1204	nii.gz	✓
		Total Segmentator v2.0	Organ Segmentation	117	1228	nii.gz	✓
		CT-ORG	Organ Segmentation	6	140	nii.gz	✓
	PET-CT	AutoPETI(2022)	Lesion Segmentation	1	1164	nii.gz	✓
		AutoPETII(2023)	Lesion Segmentation	1	1214	nii.gz	✓
		AutoPETIII(2024)	Lesion Segmentation	1	1614	nii.gz	✓
	MRI	TotalSegmentator MRI	Organ Segmentation	56	298	nii.gz	✓

Table S9: Summary of Brain Dataset within the IPIU Large-Scale Medical Imaging Dataset.

Dimensions	Modality	Dataset	Task	Targets	Data Size	File Format	Public
3D	CT	INSTANCE 2022	Lesion Segmentation	1	200	nii.gz	✓
	MRI	FeTA 2022	Organ Segmentation	7	280	nii.gz	✓
		iSeg	Organ Segmentation	3	23	nii.gz	✓
		cSeg 2022	Organ Segmentation	3	13	nii.gz	✓
		CAS2023	Organ Segmentation	1	100	nii.gz	✓
		BraTS21	Lesion Segmentation	3	2040	nii.gz	✓
		BraTS2023-SSA	Lesion Segmentation	3	105	nii.gz	✓
		BraTS2023-MET	Lesion Segmentation	3	328	nii.gz	✓
		BraTS2023-MEN	Lesion Segmentation	3	1650	nii.gz	✓
		BraTS2023-PED	Lesion Segmentation	3	228	nii.gz	✓
		BraTS-TCGA-LGG	Lesion Segmentation	3	65	nii.gz	✓
		BraTS-TCGA-GBM	Lesion Segmentation	3	102	nii.gz	✓
		MSD Brain(Task01)	Lesion Segmentation	3	750	nii.gz	✓
		MSD Hippocampus(Task04)	Organ Segmentation	1	394	nii.gz	✓
		ISLES22	Lesion Segmentation	1	400	nii.gz	✓
		ATLAS v2.0	Lesion Segmentation	1	1271	nii.gz	✓
		WMH	Lesion Segmentation	1	170	nii.gz	✓
		L2R-OASIS	Segmentation/Registration	35	416	nii.gz	✓

Table S10: Summary of Eyes Dataset within the IPIU Large-Scale Medical Imaging Dataset.

Dimensions	Modality	Dataset	Task	Targets	Data Size	File Format	Public
2D	OCTA	OCTA-500	Organ Segmentation	1	300	png	✓
		ROSE	Organ Segmentation	1	229	png, tif	✓
	Fundus Images	RFMiD 2.0	Classification	45	3200	png	✓
		JSIEC	Classification	39	1000	jpg	✓
		DRIVE	Organ Segmentation	1	40	tif, gif	✓
		PALM19	Organ Segmentation	1	1200	png	✓
		REFUGE	Classification/Segmentation	2	1200	png	✓
		Retina	Classification	4	601	png	✓

Table S11: Summary of Thorax Dataset within the IPIU Large-Scale Medical Imaging Dataset.

**Dimensions**	**Modality**	**Dataset**	**Task**	**Targets**	**Data Size**	**File Format**	**Public**
2D	Pathology	BreaKHis	Classification	2	7909	png	✓
		LungHist700	Classification	7	691	jpg	✓
		WSSS4LUAD	Lesion Segmentation	3	10211	png	✓
		ANHIR	Registration	-	481	png, jpg	✓
	CT	COVID_CT_COVID-CT	Classification	2	746	png, jpg	✓
		SARS-COV-2 Ct-Scan	Classification	2	2482	png	✓
		Chest CT-Scan images	Classification	4	1000	png, jpg	✓
		COVID-19-CT SCAN IMAGES	Classification	2	1400	png, jpg, jpeg	✓
	X-Ray	COVID-19 CHEST X-RAY	Classification	3	3886	png	✓
		Chest X-ray PD Dataset	Classification	3	4575	jpg, png	✓
		COVIDGR	Classification	2	852	jpg	✓
		MIAS	Classification	7	322	png	✓
		CoronaHack	Classification/Segmentation	7	5910	png	✓
		SZ-CXR	Classification/Segmentation	1	566	png	✓
		ChestX-Det10	Instance Detection	10	3543	png	✓
	Ultrasound	BUSI	Classification/Segmentation	3	780	png	✓
		Breast Ultrasound Dataset B	Lesion Segmentation	1	163	png	✓
3D	CT	LUNA16	Detection/Segmentation	1	888	mhd	✓
		ATM22	Organ Segmentation	1	500	nii.gz	✓
		MSD Lung Tumours(Task06)	Lesion Segmentation	1	96	nii.gz	✓
		Parse 2022	Organ Segmentation	1	200	nii.gz	✓
		StructSeg 2019 Task3	Organ Segmentation	6	50	nii.gz	✓
		StructSeg 2019 Task4	Lesion Segmentation	1	50	nii.gz	✓
		SegTHOR	Organ Segmentation	4	60	nii.gz	✓
		FUMPE	Lesion Segmentation	1	35	nii.gz	✓
		LNDb	Lesion Segmentation	1	294	nii.gz	✓
		LNQ 2023	Lesion Segmentation	1	413	nrrd	✓
		PleThora	Organ Segmentation	2	402	dicom, nii.gz	✓
	MRI	ACDC	Organ Segmentation	3	150	nii.gz	✓
		MSD Cardiac(Task02)	Organ Segmentation	1	30	nii.gz	✓
		LAScarQS 2022	Organ Segmentation	2	194	nii.gz	✓
		MyoPS 2020	Organ Segmentation	5	45	nii.gz	✓
		MM-WHS	Organ Segmentation	7	120	nii.gz	✓
		CMRxMotion	Organ Segmentation	3	360	nii.gz	✓
	Ultrasound	MVSeg-3DTEE 2023	Organ Segmentation	2	175	nii.gz	✓
		TDSC-ABUS2023	Lesion Segmentation	1	200	nrrd	✓

Table S12: Summary of Abdomen Dataset within the IPIU Large-Scale Medical Imaging Dataset.

Dimensions	Modality	Dataset	Task	Targets	Data Size	File Format	Public
2D	Pathology	Gleason 2019	Classification	6	311	jpg	✓
		PANDA	Classification	5	331	jpg	✓
		EBHI-Seg	Lesion Segmentation	6	4456	png	✓
		CRAG	Lesion Segmentation	1	213	png	✓
		AGGC	Lesion Segmentation	5	203	tif	✓
	Endoscope	Colonoscopic	Lesion Detection	2	76*2	mp4	✓
		CP-CHILD	Classification	2	9500	jpg	✓
		Kvasir-SEG	Lesion Segmentation	1	1160	jpg	✓
		CVC-ClinicDB	Lesion Segmentation	1	612	tif	✓
		EAD 2019	Organ Segmentation	7	2991	jpg, tif	✓
		PSVFMs	Organ Segmentation	1	483	png	✓
		CholecSeg8k	Organ Segmentation	13	8080	png	✓
		C3VD	Reconstruction	22	10015	png, tiff, txt	✓
3D	CT	FLARE 2021	Organ Segmentation	4	511	nii.gz	✓
		FLARE 2022	Organ Segmentation	12	2300	nii.gz	✓
		FLARE 2023	Organ Segmentation	14	4500	nii.gz	✓
		WORD	Organ Segmentation	16	150	nii.gz	✓
		AbdomenCT-1K	Organ Segmentation	4	1112	nii.gz	✓
		3D-IRCADB	Organ Segmentation	40	22	dicom, vtk	✓
		LiTS	Lesion Segmentation	2	201	nii.gz	✓
		KiTS19	Lesion Segmentation	2	300	nii.gz	✓
		KiTS21	Lesion Segmentation	3	400	nii.gz	✓
		KiTS23	Lesion Segmentation	3	599	nii.gz	✓
		BTCV	Organ Segmentation	13	50	nii.gz	✓
		BTCV Cervix	Organ Segmentation	4	50	nii.gz	✓
		CHAOS	Organ Segmentation	4	40	dicom	✓
		MSD Liver(Task03)	Lesion Segmentation	2	210	nii.gz	✓
		MSD Prostate(Task05)	Organ Segmentation	1	48	nii.gz	✓
		MSD Pancreas Tumour(Task07)	Lesion Segmentation	2	420	nii.gz	✓
		MSD Spleen(Task09)	Organ Segmentation	1	61	nii.gz	✓
		MSD Colon Cancer(Task10)	Lesion Segmentation	1	190	nii.gz	✓
		SLIVER07	Organ Segmentation	1	30	mhd	✓
		QUBIQ2021-3D CT	Lesion Segmentation	2	90	nii.gz	✓
	CTA	KiPA22	Organ Segmentation	4	130	nii.gz	✓
	MRI	AMOS 2022	Organ Segmentation	15	600	nii.gz	✓
		ATLAS	Lesion Segmentation	2	90	nii.gz	✓
		SPPIN	Lesion Segmentation	1	111	nii.gz	✓
		CHAOS	Organ Segmentation	4	40	dicom	✓
		PROMISE12	Organ Segmentation	1	50	mhd	✓
		PI-CAI	Lesion Segmentation	1	1500	mha	✓
		LLD-MMRI2023	Detection	7	394	nii.gz	✓
		PanSegData	Organ Segmentation	1	767	nii.gz	✓

Table S13: Summary of Lesion Dataset within the IPIU Large-Scale Medical Imaging Dataset.

**Dimensions**	**Modality**	**Dataset**	**Task**	**Targets**	**Data Size**	**File Format**	**Public**
2D	Pathology	WSSS4LUAD	Lesion Segmentation	3	10211	png	✓
		EBHI-Seg	Lesion Segmentation	6	4456	png	✓
		CRAG	Lesion Segmentation	1	213	png	✓
		AGGC	Lesion Segmentation	5	203	tif	✓
	Ultrasound	TN-SCUI2020	Lesion Segmentation	1	4554	png	✓
		DDTI	Lesion Segmentation	1	637	png	✓
		TG3K	Lesion Segmentation	1	3585	jpg	✓
		TN3K	Lesion Segmentation	1	3493	jpg	✓
		Breast Ultrasound Dataset B	Lesion Segmentation	1	163	png	✓
	Endoscope	Colonoscopic	Lesion Detection	2	76*2	mp4	✓
		Kvasir-SEG	Lesion Segmentation	1	1160	jpg	✓
		CVC-ClinicDB	Lesion Segmentation	1	612	tif	✓
3D	PET-CT	AutoPETI(2022)	Lesion Segmentation	1	1164	nii.gz	✓
		AutoPETII(2023)	Lesion Segmentation	1	1214	nii.gz	✓
		AutoPETIII(2024)	Lesion Segmentation	1	1614	nii.gz	✓
		HECKTOR 2022	Lesion Segmentation	2	882	nii.gz	✓
	CT	INSTANCE 2022	Lesion Segmentation	1	200	nii.gz	✓
		SegRap2023	Organ/Lesion Segmentation	45/2	200	nii.gz	✓
		MSD Lung Tumours(Task06)	Lesion Segmentation	1	96	nii.gz	✓
		StructSeg 2019 Task4	Lesion Segmentation	1	50	nii.gz	✓
		FUMPE	Lesion Segmentation	1	35	nii.gz	✓
		LNDb	Lesion Segmentation	1	294	nii.gz	✓
		LNQ 2023	Lesion Segmentation	1	413	nrrd	✓
		LiTS	Lesion Segmentation	2	201	nii.gz	✓
		KiTS19	Lesion Segmentation	2	300	nii.gz	✓
		KiTS21	Lesion Segmentation	3	400	nii.gz	✓
		KiTS23	Lesion Segmentation	3	599	nii.gz	✓
		MSD Liver(Task03)	Lesion Segmentation	2	210	nii.gz	✓
		MSD Pancreas Tumour(Task07)	Lesion Segmentation	2	420	nii.gz	✓
		MSD Colon Cancer(Task10)	Lesion Segmentation	1	190	nii.gz	✓
		QUBIQ2021-3D CT	Lesion Segmentation	2	90	nii.gz	✓
		MSD Hepatic Vessel(Task08)	Lesion Segmentation	2	443	nii.gz	✓
	MRI	BraTS21	Lesion Segmentation	3	2040	nii.gz	✓
		BraTS2023-SSA	Lesion Segmentation	3	105	nii.gz	✓
		BraTS2023-MET	Lesion Segmentation	3	328	nii.gz	✓
		BraTS2023-MEN	Lesion Segmentation	3	1650	nii.gz	✓
		BraTS2023-PED	Lesion Segmentation	3	228	nii.gz	✓
		BraTS-TCGA-LGG	Lesion Segmentation	3	65	nii.gz	✓
		BraTS-TCGA-GBM	Lesion Segmentation	3	102	nii.gz	✓
		MSD Brain(Task01)	Lesion Segmentation	3	750	nii.gz	✓
		ISLES22	Lesion Segmentation	1	400	nii.gz	✓
		ATLAS v2.0	Lesion Segmentation	1	1271	nii.gz	✓
		WMH	Lesion Segmentation	1	170	nii.gz	✓
		ATLAS	Lesion Segmentation	2	90	nii.gz	✓
		SPPIN	Lesion Segmentation	1	111	nii.gz	✓
		PI-CAI	Lesion Segmentation	1	1500	mha	✓
	Ultrasound	TDSC-ABUS2023	Lesion Segmentation	1	200	nrrd	✓

Note: Given the distinct characteristics of lesion-related tasks in terms of target scale and diagnostic relevance compared to conventional anatomical structure analysis, we separately summarize them to more specifically highlight their significance in the development of medical foundation models.

Table S14: Summary of Head and Neck Dataset within the IPIU Large-Scale Medical Imaging Dataset.

Dimensions	Modality	Dataset	Task	Targets	Data Size	File Format	Public
2D	Pathology	OSCC	Classification	2	1224	jpg	✓
	OCT	OCTMNIST	Classification	4	109309	png	✓
	Ultrasound	TN-SCUI2020	Lesion Segmentation	1	4554	png	✓
		DDTI	Lesion Segmentation	1	637	png	✓
		TG3K	Lesion Segmentation	1	3585	jpg	✓
		TN3K	Lesion Segmentation	1	3493	jpg	✓
3D	CT	NasalSeg	Classification	6	130	nrrd	✓
		SegRap2023	Organ/Lesion Segmentation	45/2	200	nii.gz	✓
		PDDCA	Organ Segmentation	9	48	nrrd	✓
	PET-CT	HECKTOR 2022	Lesion Segmentation	2	882	nii.gz	✓
	CT / MRI	HaN-Seg	Organ Segmentation	30	42	nrrd	✓
	IOS	ToothFairy	Organ Segmentation	1	443	npy	✓
		Teeth3DS	Teeth Segmentation	32	1800	obj, json	✓

Table S15: Summary of Vessel Dataset within the IPIU Large-Scale Medical Imaging Dataset.

Dimensions	Modality	Dataset	Task	Targets	Data Size	File Format	Public
3D	CTA	SEG.A.	Organ Segmentation	1	56	nrrd	✓
		ImageCAS	Organ Segmentation	1	1000	nii.gz	✓
	MR	COSMOS2022	Organ Segmentation	1	75	dicom	✓
	CT	VESSEL12	Organ Segmentation	1	20	tar.bz2	✓
		MSD Hepatic Vessel(Task08)	Lesion Segmentation	2	443	nii.gz	✓

Table S16: Summary of Skeleton Dataset within the IPIU Large-Scale Medical Imaging Dataset.

Dimensions	Modality	Dataset	Task	Targets	Data Size	File Format	Public
3D	CT	RibFrac 2020	Detection	2	1224	jpg	✓
		VerSe19	Organ Segmentation	26	160	nii.gz	✓
		VerSe20	Organ Segmentation	26	319	nii.gz	✓
		CTSpine1K	Organ Segmentation	25	1005	nii.gz	✓
		CTPelvic1K	Organ Segmentation	4	1184	nii.gz	✓
		MICCAI2024 PENGWIN	Organ Segmentation	3	100	mha	✓
	MRI	SPIDER	Organ Segmentation	19	544	mha	✓
		IVDM3Seg	Organ Segmentation	1	16	nii.gz	✓

Note: ✓ denotes the availability of a directly accessible official link or download link.

References

1. Huang Z, Wang H, Deng Z, et al. Stu-net: Scalable and transferable medical image segmentation models empowered by large-scale supervised pre-training. arXiv preprint arXiv:2304.06716 2023.
2. Ma J, He Y, Li F, Han L, You C, and Wang B. Segment anything in medical images. Nature Communications 2024;15:654.
3. Du Y, Bai F, Huang T, and Zhao B. Segvol: Universal and interactive volumetric medical image segmentation. Advances in Neural Information Processing Systems 2024;37:110746–83.
4. Sowrirajan H, Yang J, Ng AY, and Rajpurkar P. Moco pretraining improves representation and transferability of chest x-ray models. In: *Medical Imaging with Deep Learning*. PMLR. 2021:728–44.
5. Wang Z, Liu C, Zhang S, and Dou Q. Foundation model for endoscopy video analysis via large-scale self-supervised pre-train. In: *International Conference on Medical Image Computing and Computer-Assisted Intervention*. Springer. 2023:101–11.
6. Wu L, Zhuang J, and Chen H. Voco: A simple-yet-effective volume contrastive learning framework for 3d medical image analysis. In: *Proceedings of the IEEE/CVF Conference on Computer Vision and Pattern Recognition*. 2024:22873–82.
7. Zhou Z, Sodha V, Pang J, Gotway MB, and Liang J. Models genesis. Medical image analysis 2021;67:101840.
8. Liu H, Wei D, Lu D, Sun J, Wang L, and Zheng Y. M3AE: Multimodal representation learning for brain tumor segmentation with missing modalities. In: *Proceedings of the AAAI conference on artificial intelligence*. Vol. 37. 2. 2023:1657–65.
9. Chen Z, Agarwal D, Aggarwal K, Safta W, Balan MM, and Brown K. Masked image modeling advances 3d medical image analysis. In: *Proceedings of the IEEE/CVF Winter Conference on Applications of Computer Vision*. 2023:1970–80.
10. Wang J, Xie G, Huang Y, et al. FedMed-GAN: Federated domain translation on unsupervised cross-modality brain image synthesis. Neurocomputing 2023;546:126282.
11. Cai Z, Lin L, He H, Cheng P, and Tang X. Uni4eye++: A general masked image modeling multimodal pre-training framework for ophthalmic image classification and segmentation. IEEE Transactions on Medical Imaging 2024.
12. Zhuang J, Wu L, Wang Q, et al. Mim: Mask in mask self-supervised pre-training for 3d medical image analysis. IEEE Transactions on Medical Imaging 2025.
13. Gupta A, Osman I, Shehata MS, Braun WJ, and Feldman RE. MedMAE: A Self-Supervised Backbone for Medical Imaging Tasks. Computation 2025;13:88.
14. Zhang T, Wei D, Zhu M, Gu S, and Zheng Y. Self-supervised learning for medical image data with anatomy-oriented imaging planes. Medical Image Analysis 2024;94:103151.

15. Tang Y, Yang D, Li W, et al. Self-supervised pre-training of swin transformers for 3d medical image analysis. In: *Proceedings of the IEEE/CVF conference on computer vision and pattern recognition*. 2022:20730–40.

16. Wang G, Wu J, Luo X, Liu X, Li K, and Zhang S. Mis-fm: 3d medical image segmentation using foundation models pretrained on a large-scale unannotated dataset. arXiv preprint arXiv:2306.16925 2023.

17. Zhou HY, Lian C, Wang L, and Yu Y. Advancing radiograph representation learning with masked record modeling. arXiv preprint arXiv:2301.13155 2023.

18. Xie Y, Gu L, Harada T, Zhang J, Xia Y, and Wu Q. Rethinking masked image modelling for medical image representation. Medical Image Analysis 2024;98:103304.

19. Huang SC, Shen L, Lungren MP, and Yeung S. Gloria: A multimodal global-local representation learning framework for label-efficient medical image recognition. In: *Proceedings of the IEEE/CVF international conference on computer vision*. 2021:3942–51.

20. Wang Z, Wu Z, Agarwal D, and Sun J. Medclip: Contrastive learning from unpaired medical images and text. In: *Proceedings of the Conference on Empirical Methods in Natural Language Processing. Conference on Empirical Methods in Natural Language Processing*. Vol. 2022. 2022:3876.

21. Zhang Y, Jiang H, Miura Y, Manning CD, and Langlotz CP. Contrastive learning of medical visual representations from paired images and text. In: *Machine learning for healthcare conference*. PMLR. 2022:2–25.

22. Zhang X, Wu C, Zhang Y, Xie W, and Wang Y. Knowledge-enhanced visual-language pretraining on chest radiology images. Nature Communications 2023;14:4542.

23. Liu C, Cheng S, Chen C, et al. M-flag: Medical vision-language pre-training with frozen language models and latent space geometry optimization. In: *International Conference on Medical Image Computing and Computer-Assisted Intervention*. Springer. 2023:637–47.

24. Shu C, Zhu Y, Tang X, et al. Miter: Medical image–text joint adaptive pretraining with multilevel contrastive learning. Expert Systems with Applications 2024;238:121526.

25. Liu B, Lu Z, and Wang Y. Towards Medical Vision-Language Contrastive Pre-training via Study-Oriented Semantic Exploration. In: *Proceedings of the 32nd ACM International Conference on Multimedia*. 2024:4861–70.

26. Moon JH, Lee H, Shin W, Kim YH, and Choi E. Multi-modal understanding and generation for medical images and text via vision-language pre-training. IEEE Journal of Biomedical and Health Informatics 2022;26:6070–80.

27. Wu C, Zhang X, Zhang Y, Wang Y, and Xie W. Medklip: Medical knowledge enhanced language-image pre-training for x-ray diagnosis. In: *Proceedings of the IEEE/CVF International Conference on Computer Vision*. 2023:21372–83.

28. Huang W, Li C, Zhou HY, et al. Enhancing representation in radiography-reports foundation model: A granular alignment algorithm using masked contrastive learning. Nature Communications 2024;15:7620.

29. Liu J, Zhou HY, Li C, et al. Mlip: medical language-image pre-training with masked local representation learning. In: *2024 IEEE International Symposium on Biomedical Imaging (ISBI)*. IEEE. 2024:1–5.

30. Wu R, Zhang C, Zhang J, Zhou Y, Zhou T, and Fu H. MM-Retinal: Knowledge-Enhanced Foundational Pretraining with Fundus Image-Text Expertise. In: *International Conference on Medical Image Computing and Computer-Assisted Intervention*. Springer. 2024:722–32.

31. Wu B, Xie Y, Zhang Z, et al. MMCLIP: Cross-modal Attention Masked Modelling for Medical Language-Image Pre-Training. arXiv preprint arXiv:2407.19546 2024.

32. Pai S, Bontempi D, Hadzic I, et al. Foundation model for cancer imaging biomarkers. Nature machine intelligence 2024;6:354–67.

33. Lu S, Chen Y, Chen Y, et al. General lightweight framework for vision foundation model supporting multi-task and multi-center medical image analysis. Nature Communications 2025;16:2097.

34. Islam NU, Ma D, Pang J, Velan SS, Gotway M, and Liang J. Foundation X: Integrating Classification, Localization, and Segmentation Through Lock-Release Pretraining Strategy for Chest X-Ray Analysis. In: *2025 IEEE/CVF Winter Conference on Applications of Computer Vision (WACV)*. IEEE. 2025:3647–56.

35. Chen RJ, Ding T, Lu MY, et al. Towards a general-purpose foundation model for computational pathology. Nature medicine 2024;30:850–62.

36. Jiao J, Zhou J, Li X, et al. Usfm: A universal ultrasound foundation model generalized to tasks and organs towards label efficient image analysis. Medical Image Analysis 2024;96:103202.

37. Gao Z, Zhang G, Liang H, et al. A Lung CT Foundation Model Facilitating Disease Diagnosis and Medical Imaging. medRxiv 2025:2025–1.

38. Butoi VI, Ortiz JJG, Ma T, Sabuncu MR, Guttag J, and Dalca AV. Universeg: Universal medical image segmentation. In: *Proceedings of the IEEE/CVF International Conference on Computer Vision*. 2023:21438–51.

39. Gu P, Zhao Z, Wang H, et al. Boosting medical image classification with segmentation foundation model. In: *2024 IEEE International Symposium on Biomedical Imaging (ISBI)*. IEEE. 2024:1–5.

40. Tian L, Greer H, Kwitt R, et al. unigradicon: A foundation model for medical image registration. In: *International Conference on Medical Image Computing and Computer-Assisted Intervention*. Springer. 2024:749–60.

41. He Y, Guo P, Tang Y, et al. VISTA3D: A unified segmentation foundation model for 3D medical imaging. In: *Proceedings of the Computer Vision and Pattern Recognition Conference*. 2025:20863–73.

42. Zhou Y, Chia MA, Wagner SK, et al. A foundation model for generalizable disease detection from retinal images. Nature 2023;622:156–63.

43. Yan S, Yu Z, Primiero C, et al. A multimodal vision foundation model for clinical dermatology. Nature Medicine 2025:1–12.

44. Eslami S, Meinel C, and De Melo G. Pubmedclip: How much does clip benefit visual question answering in the medical domain? In: *Findings of the Association for Computational Linguistics: EACL 2023*. 2023:1181–93.

45. Bannur S, Hyland S, Liu Q, et al. Learning to exploit temporal structure for biomedical vision-language processing. In: *Proceedings of the IEEE/CVF Conference on Computer Vision and Pattern Recognition*. 2023:15016–27.

46. Chen Q and Hong Y. Medblip: Bootstrapping language-image pre-training from 3d medical images and texts. In: *Proceedings of the Asian Conference on Computer Vision*. 2024:2404–20.

47. Nie Y, He S, Bie Y, et al. ConceptCLIP: Towards Trustworthy Medical AI via Concept-Enhanced Contrastive Langauge-Image Pre-training. arXiv preprint arXiv:2501.15579 2025.

48. Liu J, Zhang Y, Chen JN, et al. Clip-driven universal model for organ segmentation and tumor detection. In: *Proceedings of the IEEE/CVF international conference on computer vision*. 2023:21152–64.

49. Zhao Z, Zhang Y, Wu C, et al. One model to rule them all: Towards universal segmentation for medical images with text prompts. arXiv preprint arXiv:2312.17183 2023.

50. Bluethgen C, Chambon P, Delbrouck JB, et al. A vision–language foundation model for the generation of realistic chest x-ray images. Nature Biomedical Engineering 2024:1–13.

51. Blankemeier L, Cohen JP, Kumar A, et al. Merlin: A vision language foundation model for 3d computed tomography. Research Square 2024:rs–3.

52. Shentu J and Al Moubayed N. CXR-IRGen: an integrated vision and language model for the generation of clinically accurate chest X-ray image-report pairs. In: *Proceedings of the IEEE/CVF Winter Conference on Applications of Computer Vision*. 2024:5212–21.

53. Lu MY, Chen B, Williamson DF, et al. A visual-language foundation model for computational pathology. Nature Medicine 2024;30:863–74.

54. Zhang K, Zhou R, Adhikarla E, et al. A generalist vision–language foundation model for diverse biomedical tasks. Nature Medicine 2024:1–13.

55. Xiang J, Wang X, Zhang X, et al. A vision–language foundation model for precision oncology. Nature 2025;638:769–78.

56. Zhao T, Gu Y, Yang J, et al. A foundation model for joint segmentation, detection and recognition of biomedical objects across nine modalities. Nature methods 2025;22:166–76.

57. Oh Y, Seifert R, Cao Y, et al. Developing a PET/CT Foundation Model for Cross-Modal Anatomical and Functional Imaging. arXiv preprint arXiv:2503.02824 2025.

58. Wan Z, Gao Y, Pang W, and Ding D. VOILA: Complexity-Aware Universal Segmentation of CT images by Voxel Interacting with Language. In: *Proceedings of the AAAI Conference on Artificial Intelligence*. Vol. 39. 7. 2025:7482–90.

59. Wu C, Zhang X, Zhang Y, Hui H, Wang Y, and Xie W. Towards generalist foundation model for radiology by leveraging web-scale 2d&3d medical data. Nature Communications 2025;16:7866.

60. Fallahpour A, Alinoori M, Ye W, Cao X, Afkanpour A, and Krishnan A. Ehrmamba: Towards generalizable and scalable foundation models for electronic health records. arXiv preprint arXiv:2405.14567 2024.

61. Zhu W, Tang H, Zhang H, et al. Predicting risk of Alzheimer's diseases and related dementias with AI foundation model on electronic health records. medRxiv 2024.

62. Sivarajkumar S, Zhang H, Ji Y, et al. Generative Foundation Model for Structured and Unstructured Electronic Health Records. arXiv preprint arXiv:2508.16054 2025.

63. Liu F, Zhou H, Wang K, et al. MetaGP: A generative foundation model integrating electronic health records and multimodal imaging for addressing unmet clinical needs. Cell Reports Medicine 2025;6.

64. Hou W, Wang J, Lin Q, Wang X, and Huang L. Improving Clinical Foundation Models with Multi-modal Learning and Domain Adaptation for Chronic Disease Prediction. IEEE Journal of Biomedical and Health Informatics 2025.

65. McKeen K, Masood S, Toma A, Rubin B, and Wang B. Ecg-fm: An open electrocardiogram foundation model. arXiv preprint arXiv:2408.05178 2024.

66. Phukan OC, Behera SR, Akhtar MM, Buduru AB, Sharma R, et al. Beyond Speech and More: Investigating the Emergent Ability of Speech Foundation Models for Classifying Physiological Time-Series Signals. arXiv preprint arXiv:2410.12645 2024.

67. Wu C, Wang H, Zhang X, Zhang C, and Bu J. Efficient Personalized Adaptation for Physiological Signal Foundation Model. In: *Forty-second International Conference on Machine Learning*.

68. Jiang WB, Fu X, Ding Y, and Guan C. Towards Robust Multimodal Physiological Foundation Models: Handling Arbitrary Missing Modalities. arXiv preprint arXiv:2504.19596 2025.

69. Hao M, Gong J, Zeng X, et al. Large-scale foundation model on single-cell transcriptomics. Nature methods 2024;21:1481–91.

70. Cui H, Wang C, Maan H, et al. scGPT: toward building a foundation model for single-cell multi-omics using generative AI. Nature methods 2024;21:1470–80.

71. Dalla-Torre H, Gonzalez L, Mendoza-Revilla J, et al. Nucleotide Transformer: building and evaluating robust foundation models for human genomics. Nature Methods 2025;22:287–97.
